# Ty3 Retrotransposon Hijacks Mating Yeast RNA Processing Bodies to Infect New Genomes

**DOI:** 10.1371/journal.pgen.1005528

**Published:** 2015-09-30

**Authors:** Virginia Bilanchone, Kristina Clemens, Robyn Kaake, Anthony R. Dawson, Dina Matheos, Kunio Nagashima, Parth Sitlani, Kurt Patterson, Ivan Chang, Lan Huang, Suzanne Sandmeyer

**Affiliations:** 1 Department of Biological Chemistry, University of California, Irvine, Irvine, California, United States of America; 2 Department of Physiology and Biophysics, University of California, Irvine, Irvine, California, United States of America; 3 Electron Microscope Laboratory, NCI-Frederick, SAIC-Frederick, Inc., Frederick National Laboratory for Cancer Research, Frederick, Maryland, United States of America; 4 Institute for Genomics and Bioinformatics, University of California, Irvine, Irvine, California, United States of America; Ohio State University, UNITED STATES

## Abstract

Retrotransposition of the budding yeast long terminal repeat retrotransposon Ty3 is activated during mating. In this study, proteins that associate with Ty3 Gag3 capsid protein during virus-like particle (VLP) assembly were identified by mass spectrometry and screened for roles in mating-stimulated retrotransposition. Components of RNA processing bodies including DEAD box helicases Dhh1/DDX6 and Ded1/DDX3, Sm-like protein Lsm1, decapping protein Dcp2, and 5’ to 3’ exonuclease Xrn1 were among the proteins identified. These proteins associated with Ty3 proteins and RNA, and were required for formation of Ty3 VLP retrosome assembly factories and for retrotransposition. Specifically, Dhh1/DDX6 was required for normal levels of Ty3 genomic RNA, and Lsm1 and Xrn1 were required for association of Ty3 protein and RNA into retrosomes. This role for components of RNA processing bodies in promoting VLP assembly and retrotransposition during mating in a yeast that lacks RNA interference, contrasts with roles proposed for orthologous components in animal germ cell ribonucleoprotein granules in turnover and epigenetic suppression of retrotransposon RNAs.

## Author Summary

Cells undergoing changes in gene expression programs such as nutritional deprivation and other stresses exhibit formation of ribonucleoprotein (RNP) complexes. In *Saccharomyces cerevisiae*, the majority of investigations to date involve analysis of P-body (PB) and stress-granule RNP formation following nutritional stress. Few studies have investigated RNP formation induced by the mating-MAP-kinase pathway. In this study, we examined how this process influences genome stability via control of retrotransposon activation. During the mating response, expression of the retrotransposon Ty3 is induced and Ty3 virus-like particles form in RNP clusters called retrosomes. We show that mating retrosomes contain PB components that are essential for Ty3 expression, re-localization of Ty3 RNA and protein from polysomes into foci, and retrotransposition. In animal germ cell lineages, PB components are found in perinuclear complexes with RNA interference suppressors of retrotransposition. We speculate that when RNA interference is relaxed and retrotransposition is observed, some members of these complexes play positive roles as we observe in budding yeast.

## Introduction

RNA processing bodies (PB) are ribonucleoprotein (RNP) granules that contain proteins associated with cytoplasmic deadenylation, decapping and 5’ to 3’ degradation of RNAs in eukaryotic cells [reviewed in [1–5]]. In previous work we showed that artificial overexpression of the long-terminal-repeat retrotransposon Ty3 in the budding yeast, *Saccharomyces cerevisiae*, causes formation of foci of Ty3 RNA and protein and virus-like particles (VLPs) and that components of PB are required for retrotransposition [6, 7]. Formation of these foci is disrupted by the artificial overexpression of Ty3 RNA and Gag3 assembly mutants [8–12]. These and other data support the interpretation that these foci represent VLP assembly factories or retrosomes [8, 13]. Ty3 is naturally induced and retrotransposes in mating cells [14, 15], and mating cells form foci containing PB components [16]. The present investigation was undertaken to identify proteins that associate with Ty3 capsid proteins during VLP assembly, to determine whether Ty3 expression causes the formation of RNP granules reported in mating cells, and to determine the roles of these in mating cell retrotransposition.

RNP formation is associated with cells undergoing changes in gene expression programs including cells undergoing nutritional deprivation and other stresses [17–20]. Yeast PB contain components of deadenylation-dependent degradation [1]. These include CCR4-Not1-Pop2 deadenylation complex proteins; translational repressor and decapping enhancer DEAD box helicase Dhh1/DDX6 [21]; Ded1, DEAD box helicase [22]; translational repressor, decapping enhancer, and scaffolding factor Pat1; scaffolding factor Edc3 [23]; decapping proteins Dcp1 and Dcp2; and 5’ to 3’ exonuclease Xrn1 [24]. Although it was originally thought that yeast RNPs that contained deadenylation dependent degradation factors were PB dedicated to RNA turnover, a more complex view is emerging. In addition to concentrating factors associated with RNA degradation, RNPs contain RNAs that can resume translation [25, 26] and RNPs dominated by PB components can sponsor formation of stress granules (SG) the function of which is to sequester non-translating RNAs [19, 27–29]. SG and PB contain overlapping components with SG generally lacking deadenylation and decapping factors and containing poly(A) RNA binding proteins, translation initiation factors and small ribosomal proteins in addition to non-translating RNAs [1, 30].

The finding that overexpressed Ty3 proteins and RNA concentrate with PB factors was surprising not only because it suggested that Ty3 poly(A) RNA was somehow resistant to degradation, but because in animal cells PB components include proteins implicated in retroelement restriction [31–33]. These include APOBEC cytidine deaminases [34–37]; DEAD box helicase MOV10 [38, 39]; and some fraction of Argonaute/PIWI components of RNAi [40–42]. Despite the absence of RNA interference (RNAi) in budding yeast, Dicer and Argonaute components introduced from related yeasts, suppress Ty1 retrotransposition [43].

The association between Ty3 overexpression and formation of RNPs in growing cells was especially interesting because the regulation of formation of PB and their differentiation from SG is incompletely understood. In yeast, Edc3 and Pat1 are important for assembly of PB with Edc3 considered to play a scaffolding role and Pat1 interacting with several other components [44–46]. Proteins with P/Q/N rich or prion-like domains are present in PB and at least some of these, including yeast Lsm4 subunit of the Lsm1-7 also foster PB formation [45, 47]. In addition to these structural interactions, RNPs are dynamic in response to conditions signaled through modifications of protein and RNA components. In animal cells O-linked glycan modification of proteins enhances inclusion in PB [48], but this modification has not been reported in budding yeast. YTH and Tudor [49] domain proteins are found in PB, resulting in enrichment of RNAs containing M6A [50] or proteins, for example Sm domain proteins or PIWI proteins, containing di-methyl arginine [51], respectively. CAMP-dependent PKA phosphorylation of PB component Pat1 inhibits its association with other PB components, and reversal leads to PB, but not SG formation [52]. In addition, the mitogen-activated protein (MAP) kinase signaling pathway is implicated in signal transduction from the environment to cellular components of PB. For example, phosphorylation of Dcp2 by Ste20 kinase, an upstream kinase in MAP-kinase signaling, is required for Dcp2 accumulation in PB [53].

Two major classes of long terminal repeat (LTR) retrotransposons, Ty1/Copia and Ty3/Gypsy, are expressed in *S*. *cerevisiae* during periods when expression programs are changing in response to environmental signals. Surprisingly, genetic screens for host factors discovered that PB components enhanced retrotransposition of both classes of elements [7, 54]. Ty3, the subject of this investigation, is 5.4 kb in length, including LTRs of 340 bp. In the *S*. *cerevisiae* reference strain BY4741 there are two endogenous full-length Ty3 elements and thirty-eight additional Ty3 LTRs, most likely formed by recombination between the LTRs of full-length elements (reviewed in [8]). In animal cells retrotransposons are de-repressed and retrotranspose during transient de-methylation in germ cell lineages [33, 40]. In a somewhat parallel cycle, Ty3 proliferates into new genetic backgrounds by retrotransposing in haploid mating cells [14, 15]. Exposure of mating type *MAT* ***a*** and *MAT*α cells to pheromone from the opposite mating type stimulates the mating MAP- kinase pathway [reviewed in [55]]. This pathway initiates changes to enable haploid cells of the two types to form diploid zygotes by inter-cellular fusion. Among these changes, cells arrest in G1 to synchronize cell cycles and induce expression of Ty3 fifty to eighty-fold [14, 56].

Ty3 5.2-kb genomic RNA (gRNA) encodes precursor proteins in two overlapping open reading frames, *GAG3* and *POL3* [57]. RNA is translated into the 290-aa structural precursor polyprotein, Gag3, containing capsid (CA) and nucleocapsid (NC) domains and into a 1547-aa Gag3-Pol3 precursor polyprotein. In addition to the Gag3 domain, this polyprotein contains catalytic protein domains representing protease (PR), reverse transcriptase (RT), and integrase (IN). Interactions between Gag3 domains facilitate formation of virus-like particles (VLPs) [58]. VLP formation is associated with activation of Ty3 PR, resulting in processing of polyprotein into mature domains [59]. After haploid cells mate and the cell cycle resumes, cDNA synthesis occurs [60]. The cDNA, together with a subset of VLP proteins including IN, is translocated through the nuclear pore complex and integrated at chromosomal targets. Expression of Ty3 from a high-copy- number plasmid under the *GAL1-10* promoter causes formation of clusters of assembling VLPs referred to as retrosomes [6].

The current study was undertaken to identify the proteins associated with Ty3 components during VLP formation, to determine how the RNP foci observed in mating cells [16] relate to PB and SG, whether Ty3 expression causes these mating cell RNPs to form, and to test the possible roles of these RNPs in Ty3 retrotransposition. Proteins that interact with Ty3 structural protein Gag3 were identified by mass spectrometry. Viable mutants lacking these were screened for differences from WT in pheromone-induced retrotransposition. We found that activation of the mating MAP-kinase pathway stimulated formation of foci that concentrated multiple PB, but not SG components and Ty3 RNA and protein. Expression of full-length Ty3 enhanced formation of these foci in one mutant background and formation was strongly correlated with retrotransposition. Individual PB components were found to be essential for Ty3 expression, formation of retrosomes, and re-localization of Ty3 RNA and protein from polysomes into foci.

## Results

### Pheromone induction causes PB proteins to associate with Gag3 foci

Genetic screens are a powerful way to identify host factors, but are limited with respect to identifying redundant and essential functions and informing as to whether interactions are direct or indirect. To circumvent these limitations, proteins that physically interact in a complex with Ty3 Gag3 were identified using a mass spectrometry (MS) approach. To increase the sensitivity of our analysis, the strain BY4741 (Open Biosystems) was cured of two particle types encoded by killer dsRNA, L-A and L-BC, to yield strain yVB1586 (S1 Text and S1 and S3 Tables). YVB1586 cells in logarithmic phase were induced to express Ty3 and hyper-active Ty3 variant, K15A (S3 Table) for 2 h. Cells expressing Ty3 were broken in a mechanical mixer in liquid N_2_ (Retsch, Inc.), and the solubilized fraction was separated by chromatography in parallel over control IgG and anti-VLP IgG matrices. Proteins were processed and subjected to liquid chromatography and in-line mass spectrometry (LC MS/MS) Peptides were analyzed by 1DLC-MS/MS using LTQ-Orbitrap XL MS (ThermoElectron) and data were searched and analyzed as described (Materials and Methods)[61]. This analysis identified a set of 154 proteins, of which 106 are not essential (S4 Table). Twenty-four percent of the proteins were designated by Gene Ontology Slim Process (http://www.yeastgenome.org/) as mRNA-binding proteins. This represented significant enrichment for RNA-related proteins over the 2.7% representation in the genome overall (S5 Table, S1 Fig).

To identify Gag3-interacting host factors that specifically restrict or enhance retrotransposition in mating cells, BY4741 (*MAT* **a**) or gene knockout collection derivative strains were treated with the pheromone, α-factor, to induce expression of Ty3 under the native promoter. Ty3 was carried on a plasmid and tagged with *his3AI* (Ty3-*his3AI*)[62]. Ty3 transcripts are spliced, but *HIS3* transcripts expressed in the opposite orientation are not. Therefore cells that have undergone retrotransposition can be directly identified by the His+ phenotype. Eight of these strains had increased transposition of more than twofold; transposition went down by more than two-fold in 30 strains. The screen identified fourteen genes encoding translation initiation factors, and components of PB and SG RNPs. The functions of these genes and the effect of deletion on retrotransposition are described in Table 1. Five strains lacking PB (Dcp2, Lsm1, Stm1) or PB and SG (Xrn1, Dhh1/DDX6) proteins and three strains lacking SG (Eap1, Pub1, and eIF4G1) proteins were decreased for Ty3 retrotransposition by greater than two-fold. This was not due to changes in the relative efficiency of splicing of the Ty3-*his3AI*-reporter in the mutant strains tested (S2 Fig). Six strains lacking PB (Edc3, Pat1, and Sbp1) and SG (Pbp1, Pbp4, and eIF4G2) proteins did not show significant differences from WT. Strains that failed to support WT transposition levels were analyzed further to understand their roles in Ty3 retrotransposition.

**Table 1:** Ty3 transposition results in identified strains.

Strain	Function	Human gene	Transpn x 10^-4^	*P* ^2^	Fold decr	Loc^3^
WT			5.41 ± 2.43		1.0	
WT unind			<0.01		>541	
*cdc33Δ*	Translation initiation factor	eIF4E	inviable			P,S
*dcp2 Δ* ^1^	Decapping enzyme	DCP2	2.2 ± 0.1	0.0010	2.5	P
*ded1Δ*	Translation, mRNA export	DDX3	inviable			P,S
*dhh1Δ*	Decapping enhancer; translation repressor	RCK/DDX6	<0.01		>541	P,S
*eap1Δ*	eIF4E-Binding Protein		0.27 ± 0.11	<0.0001	20.0	P,S
*edc3Δ* ^1^	Decapping enhancer	EDC3	5.48 ± 3.44	0.9472	1.0	P
*lsm1Δ*	Decapping enhancer	LSM1	1.50 ± 0.87	<0.0001	3.6	P
*lsm5Δ*	Decapping enhancer	LSM5	inviable			P
*pat1Δ*	Decapping enhancer; translation repressor	PATL1	3.75 ± 1.47	0.0518	1.4	P
*pbp1Δ*	Translation, polyadenylation regulator	ATXN2	5.31 ± 0.59	0.9367	1.0	P,S
*pbp4Δ*	PBP1 binding protein		4.92 ± 1.87	0.7334	0.9	S
*pub1Δ*	Poly (A)+ RNA-binding protein	TIA-1	1.64 ± 0.37	<0.0001	3.3	S
*rpg1Δ*	Translation initiation factor eIF3a	EIF3A	inviable			S
*sbp1Δ*	Translation repressor, binds eIF4G		4.24 ± 1.80	0.3523	1.3	P
*stm1Δ (YLR150W)*	Translation regulation		1.36 ± 0.20	<0.0001	4.0	
*tif4631Δ*	Translation initiation factor eIF4G1	eIF4G	1.60 ± 0.53	0.0029	3.4	P,S
*tif4632Δ*	Translation initiation factor eIF4G2	eIF4G	4.90 ± 0.18	0.7204	1.1	P,S
*xrn1Δ*	5 ‘ -3‘ Exonuclease	XRN1	1.43 ± 0.72	0.0067	3.8	P,S

Transposition results are mean ± SD.

^1^not identified in mass spectrometry experiment

^2^ *t* test assuming equal variances (two-tailed)

^3^Localization: P, P body; S, stress granule

### VLP morphogenesis occurs after pheromone treatment

**Fig 1:**
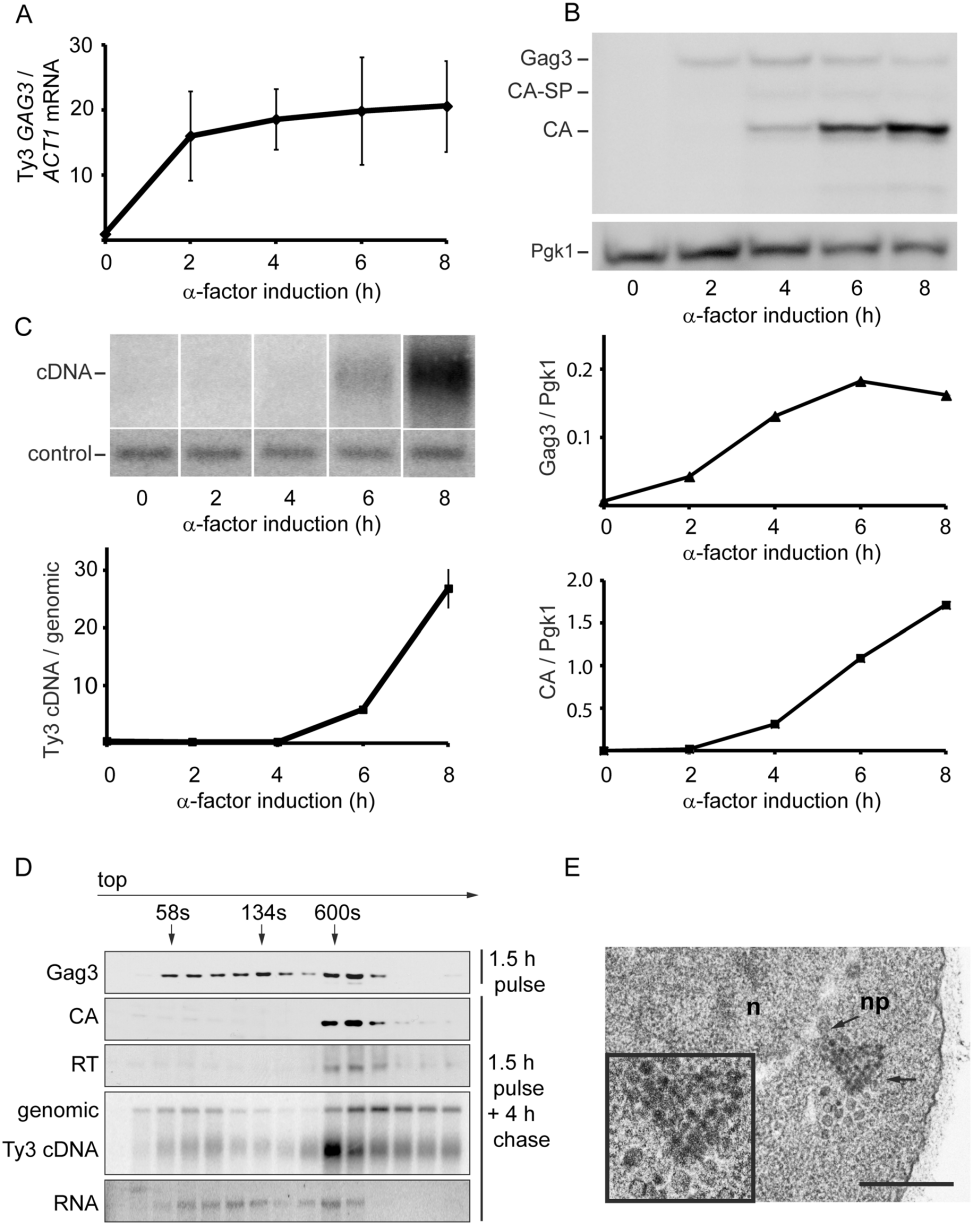
Time course of pheromone-induced production of Ty3 retrotransposition intermediates. **(A)** Gag3 RNA levels determined by RT-qPCR. **(B)** Quantification of Gag3 protein product levels and Gag3 processing determined by immunoblot analysis using α-CA antibody and α-Pgk1 antibody as control. **(C)** Ty3 cDNA levels determined by Southern blot analysis and endogenous element fragment as control. **(D)** Ty3 assembly intermediates identified in α-factor pulse-chase experiment by fractionation of cell extracts by velocity sedimentation. Cell culture was induced by α-factor for 1.5 h (top panel) or followed by a 4 h chase (bottom panels). Proteins were extracted from gradient fractions and analyzed for Ty3 proteins by immunoblot, DNA by Southern blot and RNA by northern blot. (S1 Text, Supporting Materials and Methods). **(E)** TEM image of Ty3 VLPs located adjacent to a nuclear pore after induction by α-factor for 6 h. n, nucleus; np, nuclear pore; arrow, position of three-fold enlarged image (inset). Scale bar = 500 nm.

To establish the timeframe of assembly of Ty3 VLPs and cDNA synthesis in cells undergoing pheromone induction, haploid yeast strain *MAT* **a** BY4741 (Table 1), that contains two endogenous Ty3 elements (yGRWTy3-1 and yILWTy3-1), was exposed to pheromone from *MAT*α cells (α-factor) for 2 h (Fig 1). Levels of the 5.2-kb Ty3 gRNA increase dramatically between 0 and 2 h of induction (Fig 1A) [14]. Gag3 polyprotein precursor was detected by 2 h and continued to accumulate up to the 8 h sampling. The Gag3-Pol3 fusion product of frameshifting is made at about one-twentieth the level of Gag [63] and was not detected under these experimental conditions. Gag3-Pol3 contains the PR domain. Upon VLP formation the Ty3 aspartyl protease, PR, is activated to convert Gag3 into CA, spacer and NC, and Gag3-Pol3 additionally into PR, RT, and IN species [59]. Appearance of these forms is consistent with substantial formation of VLPs between 4 and 6 h [9] (Fig 1B). Ty3 VLP maturation occurs in G1, but cDNA synthesis is delayed until after cells undergo fusion to form diploid zygotes and resume the cell cycle in S phase [60]. Between 6 and 8 h, haploid cells recover from pheromone arrest and cDNA synthesis is substantial (Fig 1C).

To understand the relative distribution of cytoplasmic Ty3 components between individual and multimerized forms, cell extracts were analyzed by sedimentation. Cells were pulsed with pheromone for 1.5 h and washed to allow recovery from pheromone arrest. Four hours after initiation of pheromone treatment, VLP formation was assessed by velocity gradient sedimentation. Western, northern, and Southern analysis of gradient fractions indicated that processed VLP components CA, RT, and cDNA concentrated within a few high-density fractions (Fig 1D). The majority of Ty3 RNA was not present in this fraction. The most likely explanation is incomplete packaging, since previous results indicate that after induction for 6 h, only 20–23.4% Ty3 RNA is packaged [9, 10]. The appearance of Ty3 cDNA near the top of the gradient suggests that mature VLPs are unstable under gradient conditions.

To visualize VLPs, BY4741 cells treated with pheromone for 6 h were examined by transmission electron microscopy (TEM). In induced cells (Fig 1E) VLPs accumulated in clusters that were not apparent in uninduced cells. Based on previous analysis of WT, PR and RT mutant VLPs [6, 12], these were a mixture of immature forms with less dense centers and mature forms with dense centers. Overall, mating-cell VLPs were more heterogeneous in morphology than VLPs from the *GAL1*-induced WT Ty3 element. The inactive YILTy3-1 contains a frameshift in the IN-coding domain [64] and this could contribute to the heterogeneity of mating-cell VLPs.

### PB proteins and Ty3 Gag3 localize in foci after pheromone treatment

**Fig 2:**
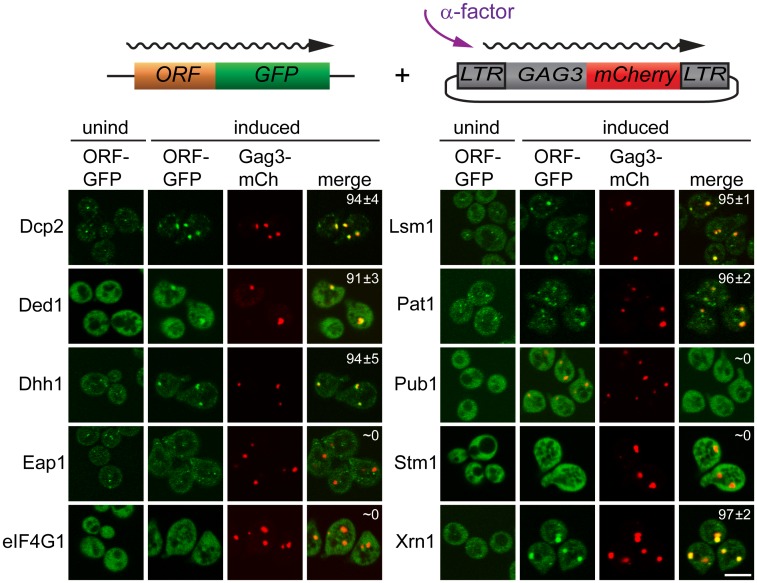
PB proteins localize with Ty3-Gag3 at 4 h of pheromone treatment. Live cells expressing PB and SG proteins under the native promoters fused to GFP and expressing Gag3-mCh (pVB3734) under the native Ty3 promoter were induced for Gag3-mCh expression by α-factor treatment for 4 h or left untreated and imaged by confocal microscopy as described (Materials and Methods, S1 Text, Supporting Materials and Methods). Insert: colocalization of GFP and mCh foci (% ± SD). Scale bar = 5 μm.

Having identified proteins that interact with Gag3 and are required for pheromone-induced retrotransposition, we asked whether they are present in pheromone-induced VLP clusters. Strains derived from BY4741 expressing Dcp2, DEAD box helicase Ded1/DDX3, DEAD box helicase Dhh1/DDX6, Edc3, Lsm1, Pat1; Eap1, eIF4G1/Tif4631, Pub1, and Xrn1 fused to green fluorescent protein (GFP) under their respective native promoters (Open Biosystems, Inc.) were transformed with low-copy- number Ty3 plasmid pVB3734 expressing Gag3 fused to mCherry (mCh). Cells in logarithmic phase were either treated with pheromone or left untreated, and were visualized after 4 h by confocal fluorescence microscopy (Fig 2). In the absence of induction, Gag3-mCh fluorescence was minimal and GFP reporters showed patterns varying from diffuse to punctate. Induced cells formed one or a few clearly distinct Ty3 Gag3-mCh foci. PB reporters Dcp2-, Ded1-, Dhh1/DDX6-, Lsm-, Pat1, and Xrn1-GFP fusions formed or enlarged foci that overlapped with these Ty3 foci. Stm1 has genetic interactions with PB factors, but has not been reported to localize to PB foci [65]. The pattern of Stm1-GFP was unafffected by Ty3 expression. Eap1-GFP SG protein reporter formed punctate foci, but these foci seemed qualitatively unaffected by pheromone induction. PB-SG reporter eIF4G1-GFP was robustly expressed and upon pheromone treatment did not form foci in pheromone induced cells. Under conditions in which Gag3-mCh foci formation occurred, SG protein Pub1-GFP was also strongly expressed, but foci were not detected in uninduced or induced cells. Overall, these results indicated that mating cells form foci containing multiple components of PB, but do not concentrate RNP proteins more typically associated with SG into these foci. In cells that formed these pheromone-dependent foci, PB-GFP reporters co-localized withTy3-mCh.

### PB factors required for retrotransposition are required for Gag3 foci

**Fig 3:**
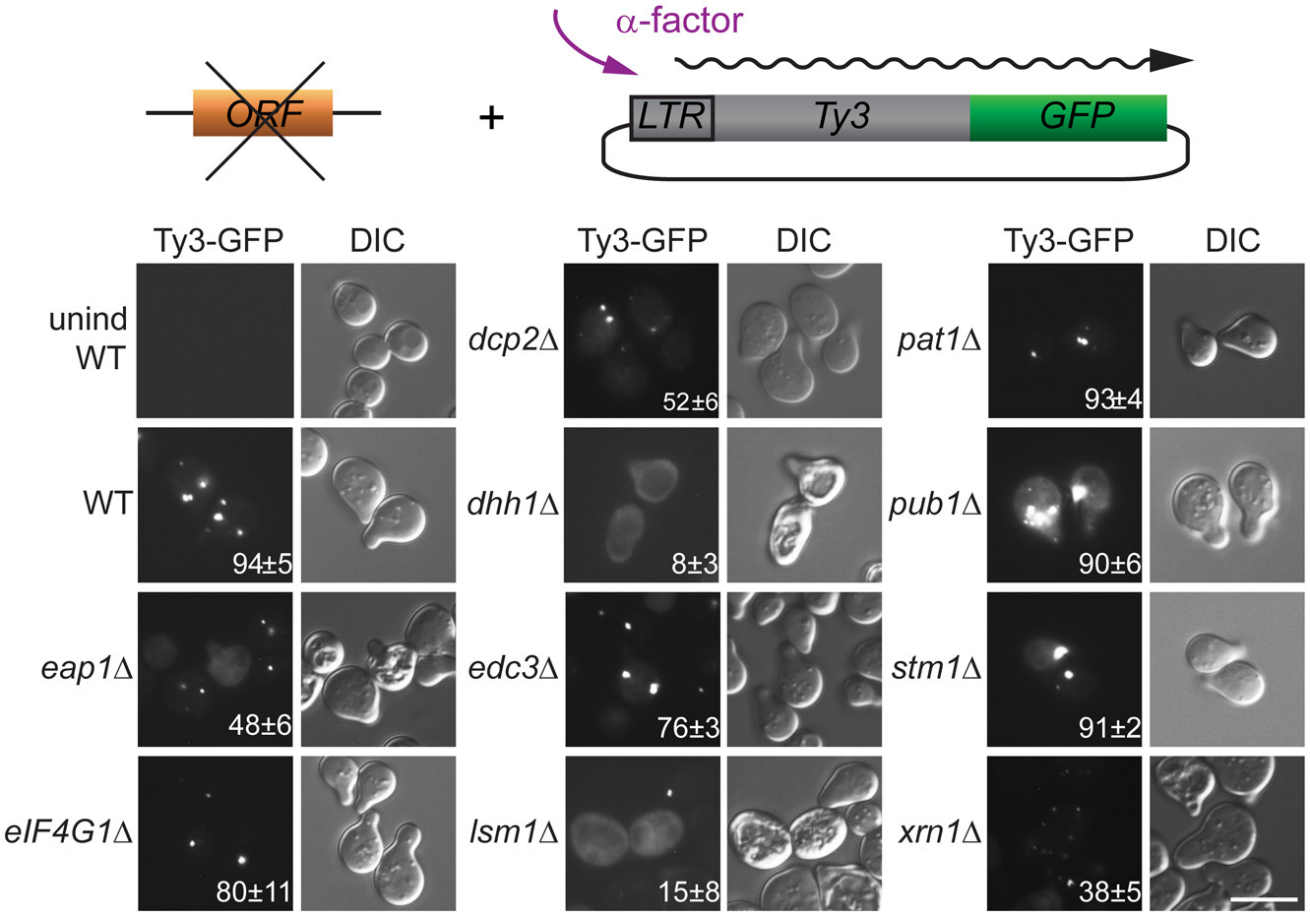
Ty3 Gag3 foci formation is disrupted in cells depleted for PB host factors. WT (BY4741) and derivatives deleted for individual ORFs and containing plasmid-borne Ty3-GFP in which GFP is fused to the end of the *POL3* ORF of native Ty3 (pTD3548), were induced with α-factor for 4 h or left untreated and imaged by widefield microscopy as described (Materials and Methods, S1 Text, Supporting Materials and Methods). Insert indicates % cells with GFP foci (mean ± SD). Scale bar = 5 μm.

The regulation of PB formation is not completely understood. However, PB component Edc3 is thought to provide a physical scaffold for PB formation in glucose deprived cells, and Pat1, that interacts with Edc3 and multiple additional PB components, contributes significantly to PB assembly [29, 44]. Deficiencies in either of these components reveal an additional contribution of a prion-like domain in Lsm4 [45] We previously reported that galactose-induced, high-copy-number Ty3 expression can drive visible PB formation in logarithmic phase cells [6]. Because evidence presented here and in previous studies of foci of mutant Gag3 suggests that Gag3 physically associates with PB proteins [12] and is multimeric [58, 66] we sought to understand whether Ty3 foci could form in the absence of PB structural proteins. A low-copy-number Ty3 reporter created by fusion of GFP in-frame to *POL3* (Ty3-GFP, pTD3548) was used to monitor Ty3 focus formation (Fig 3, S7 Table). We used deletion mutants to test effects of the loss of PB/SG components Eap1, Dhh1/DDX6, Dcp2, Edc3, Lsm1, Pat1, Pub1, eIF4G1, or Xrn1 and PB-related factor Stm1 for ability on Ty3 foci formation. By 4 h, the induced WT strain showed Ty3-GFP foci in approximately 94±5% of cells. In contrast, strains lacking proteins that both localized to foci and were required for retrotransposition showed significantly reduced percentages of cells with Ty3-GFP foci. In order of effect, these were: *dhh1Δ* (8±3%, p˂0.0001) < *lsm1Δ* (15±8%, p ˂0.0001) < *xrn1Δ* (38±5, p ˂0.0001) <*dcp2Δ* (52±6%, p ˂0.0001). The strain lacking Eap1, which was required for retrotransposition, but did not concentrate in foci, also showed reduced formation of Gag3 foci (48±6%, p ˂0.0001). Decreased percentages of cells with Ty3 foci might reflect reduced amounts of Ty3 protein, disruption of foci formation or a combination of these effects. These possibilities were investigated further as described below. Lack of Stm1 or Pub1, which were required for retrotransposition, but did not concentrate in foci, did not reduce the number of cells with Ty3 foci (*pub1Δ*, 90±6%, p = 0.5066; *stm1Δ*, 91±2%, p = 0.778). In the case of *pub1Δ*, foci appeared larger, although this was not quantified. Notably, lack of Edc3 and Pat1, proteins identified as contributing to assembly of glucose-deprivation PB in fermenting yeast [45] showed lesser effects on mating-cell PB formation (*edc3Δ* = 76±%, p = 0.0025 and *pat1Δ* = 93±4%, p = 0.857). This result is in line with the lack of effect of deletion of these proteins on Ty3 retrotransposition frequency (Table 1). Together these results reinforce the interpretation that mating cell Ty3-PB foci are more closely related to PB than SG, but differ significantly from glucose-deprivation PB in that their formation is independent of Edc3 and Pat1 PB assembly factors.

### Mating PB are enhanced by cell-cycle arrest and Ty3 expression

**Fig 4:**
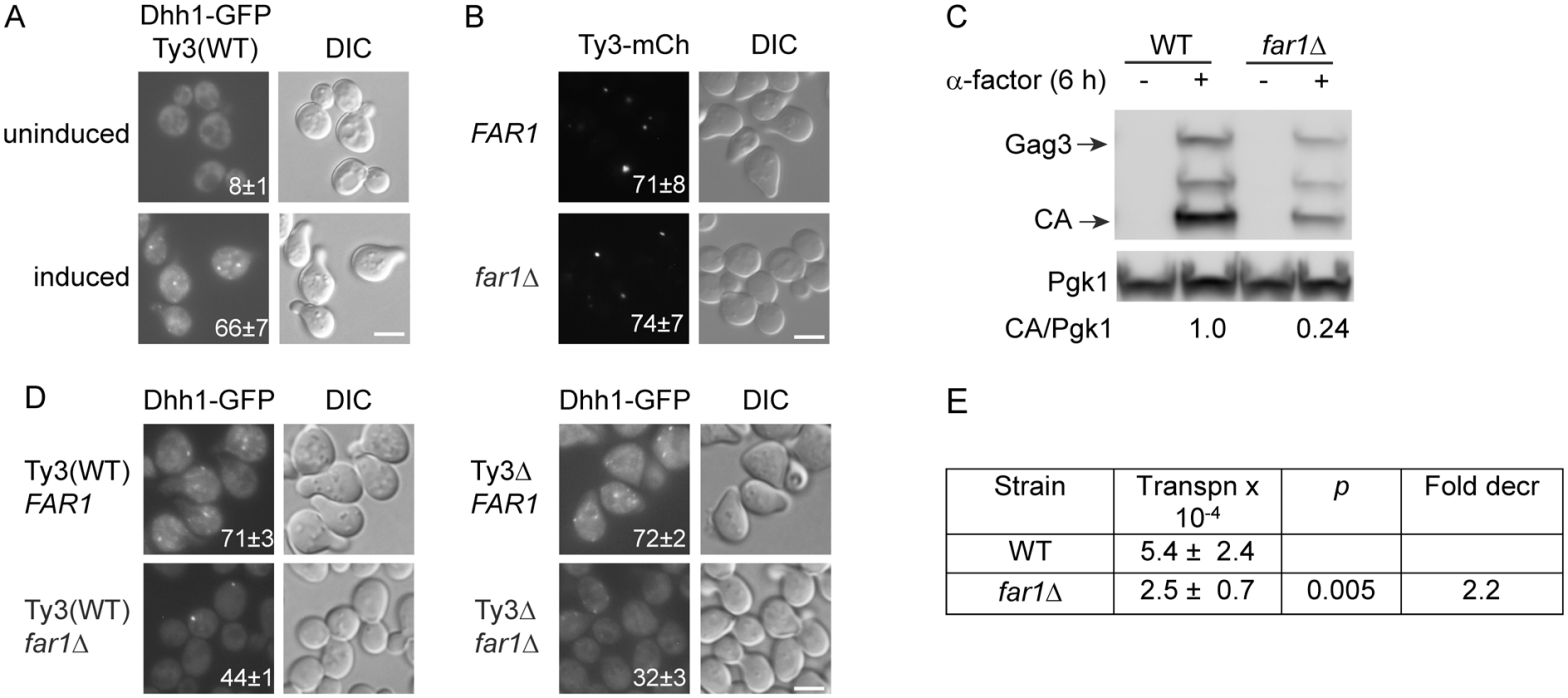
Ty3 expression enhances, but is not required for PB formation. Dhh1-GFP foci were evaluated in Ty3 WT and null cells treated with α-factor for 4 h or left untreated. Cells in **(A, B, C)** were imaged by widefield microscopy as described (Materials and Methods, S1 Text, Supporting Materials and Methods). Scale bar = 5 μm. **(A)** Pheromone treatment causes PB formation in Ty3(WT) cells. Insert indicates % cells with GFP foci (mean ± SD). **(B)** Ty3 foci are present in similar percentages of WT and *far1Δ* cells. Insert indicates % cells with Ty3-mCherry foci (mean ± SD). Cells contained Ty3-mCh-expressing plasmid under the native promoter (pTD3655). **(C)** Ty3 expression is reduced in *far1Δ* cells. Western blot analysis of Gag3 proteins products in WT and *far1Δ* cells, either uninduced or induced with α-factor. Ratio of CA/Pgk1 loading control is average of three independent cultures. **(D)** Pheromone induced Ty3 expression enhances PB formation in *far1Δ* cells. Insert as described in **(A)**. **(E)** Ty3 transposition is reduced in *far1Δ* cells. Pheromone-induced transposition was as described (Materials and Methods).

Evidence that mating cell PB contain Ty3 proteins and are resistant to effects of deletions of scaffolding proteins, together with previous results showing that Ty3 expression at high levels actually drives formation of PB [6], suggested that expression of endogenous retrotransposons contributes to formation of mating cell PB foci. Although many retrotransposons including Ty1 are present in high-copy number, full-length Ty3 is represented by only two full-length copies in the Ty3(WT) strain, BY4741 [67], enabling a direct test of its role in mating-cell PB formation. The two endogenous full-length Ty3 elements were deleted from BY4741, creating the Ty3Δ strain (yVB1672). PB formation in these strains was monitored by C-terminal fusion of the chromosomal locus of the PB component *DHH1* with GFP. The Ty3(WT) strain was treated with pheromone for 4 h or left untreated, and examined by fluorescence microscopy. As expected, induced cells showed 66% Dhh1-GFP foci compared to 8% in uninduced cells (Fig 4A).

G1 arrest during mating enables synchronous resumption in S phase after zygote formation [68]. To investigate the effect of pheromone-induced cell cycle arrest on PB formation, strains were constructed that are defective in cell cycle arrest, but are otherwise responsive to pheromone. Pheromone stimulation of the mating MAP-kinase pathway activates downstream MAP-kinase Fus3, that phosphorylates Far1, activating its inhibition of Cdc28-Cln, and culminating in G1 arrest. The *far1Δ* cells retain significant pheromone-mediated transcriptional induction, but fail to undergo efficient G1 arrest in preparation for mating. To assess the contribution of Far1-mediated cell cycle arrest on Ty3 focus formation, *FAR1* and *far1Δ* strains containing Ty3-mCherry (pTD3655) were induced with pheromone and Ty3 focus was measured (Fig 4B). No significant difference was observed between *FAR1* and *far1Δ* strains in the proportion of cells with Ty3-mCherry foci despite reduced Ty3 protein in *far1Δ* cells (Fig 4C).

To test whether Ty3 expression during pheromone-induced cell cycle arrest affected PB formation, Dhh1-GFP focus formation after pheromone induction was compared in Ty3(WT) and Ty3Δ strains with and without Far1. The results showed that PB formation as measured by Dhh1-GFP foci formation was indistinguishable between Ty3(WT) and Ty3Δ cells [Ty3(WT) = 71±3% versus Ty3Δ = 72±2%] showing that PB focus formation after pheromone treatment was not solely dependent on Ty3 expression. However, deletion of *FAR1* from Ty3(WT) cells resulted in reduced Dhh1-GFP foci formation (*FAR1* = 71±3% versus *far1Δ* = 44±1%), indicating that cell-cycle arrest contributes to PB formation. Interestingly, in *far1Δ* cells Dhh1-GFP foci formation was reduced significantly in the absence of Ty3 [Ty3(WT) *far1Δ* = 44±1% versus Ty3Δ *far1Δ* = 32±3%, p = 0.0015]. This result suggested that Ty3 expression although reduced in *far1Δ* cells nonetheless plays a positive role in PB formation, possibly more apparent when foci formation is attenuated (Fig 4D). Consistent with decreased expression of Ty3 in the *far1Δ* background, retrotransposition was decreased by about twofold (Fig 4E). Thus, MAP kinase-controlled cell-cycle arrest contributes to mating cell PB formation and in this genetic background, induction of Ty3 expression during mating enhances, but is not essential for mating PB formation.

### Ty3 expression effects on mating efficiency

**Fig 5:**
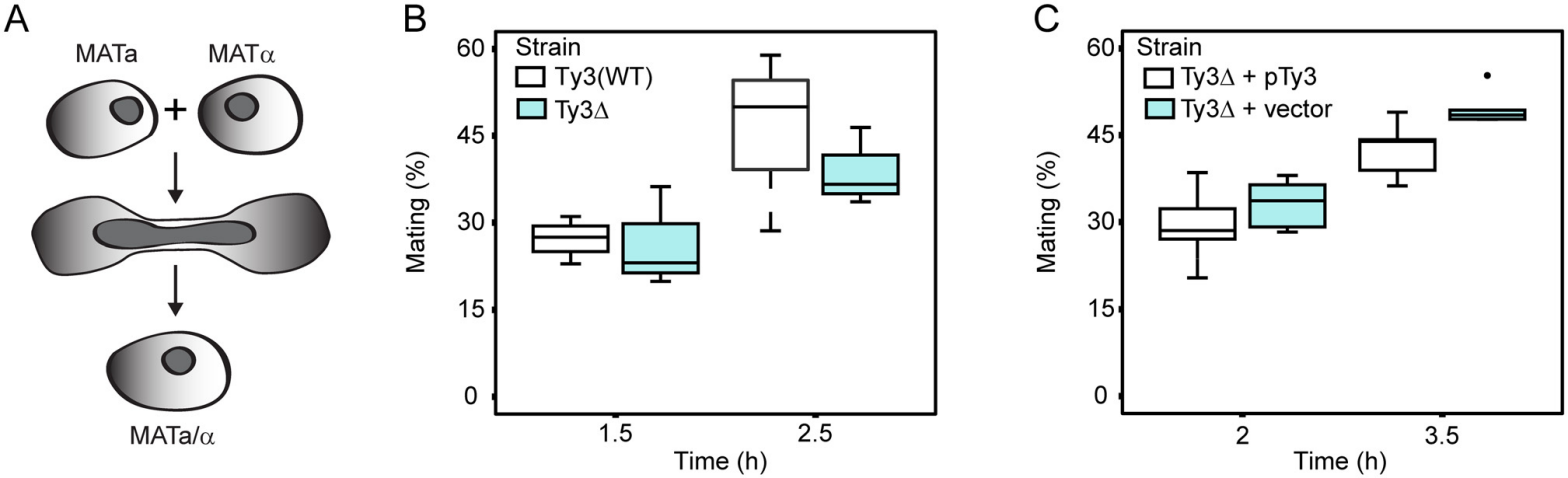
Ty3 expression effects on mating efficiency. Equal numbers of cells of each mating type either lacking Ty3 elements, or expressing Ty3 under the native promoter were mated for the time indicated. Mating efficiency (% mating) was determined as described Materials and Methods. **(A)** Yeast cells of two mating types fuse to form diploids. Mating efficiency in the presence and absence of endogenous Ty3 elements. **(B)** Mating efficiency of WT strains [BY4741 (*MAT* **a**) and BY4742 (*MATα*); two endogenous Ty3 elements] and Ty3Δ strains [yVB1913 (*MAT* **a**) and yDM1456 (*MATα*); BY4741 background with Ty3 elements deleted]. **(C)** Mating efficiency in the presence and absence of high-copy Ty3. Mating of Ty3Δ strains (yVB1672 (*MAT* **a**) and yVB1926 (*MATα*); BY4741 background with Ty3 elements deleted and replaced by *lox*P) transformed with either a high-copy-number plasmid containing Ty3 (pDM3194) or a plasmid control (pYES2.0).

Yeast PB segregate preferentially into daughter buds [69]. Furthermore, maternal RNA granules in metazoans, affect segregation of important RNAs in subsequent cell divisions. The observation that Ty3 contributes to PB formation suggested that mating might be influenced, either positively or negatively, in Ty3-populated strains. We therefore constructed derivatives of Ty3(WT) and Ty3Δ strains with appropriate nutritional markers to allow selection of diploid cells (see Materials and Methods). These strains were mixed and concentrated onto filters to promote mating for 1.5 or 2.5 h. Mated cells were spread onto double drop-out medium to select for diploid prototrophs with complementing nutritional markers. Mating efficiency at 1.5 h and 2.5 h was similar in Ty3(WT) and Ty3Δ strains (Fig 5A and 5B). To test whether a higher level of Ty3 expression produced an effect, the experiment was performed in the Ty3Δ background in the presence and absence of a high-copy-number plasmid from which Ty3 was expressed under the native promoter. In this context, Ty3 expression slightly reduced average mating efficiency at 3.5 h, but given the variation within the experiment this difference was not significant (Ty3Δ+pTy3 = 42.5±5.0% (mean ± SD) versus Ty3Δ+vector = 49.5±3.0%, p = 0.052) (Fig 5C).

### PB factors are required at multiple stages of VLP morphogenesis

**Fig 6:**
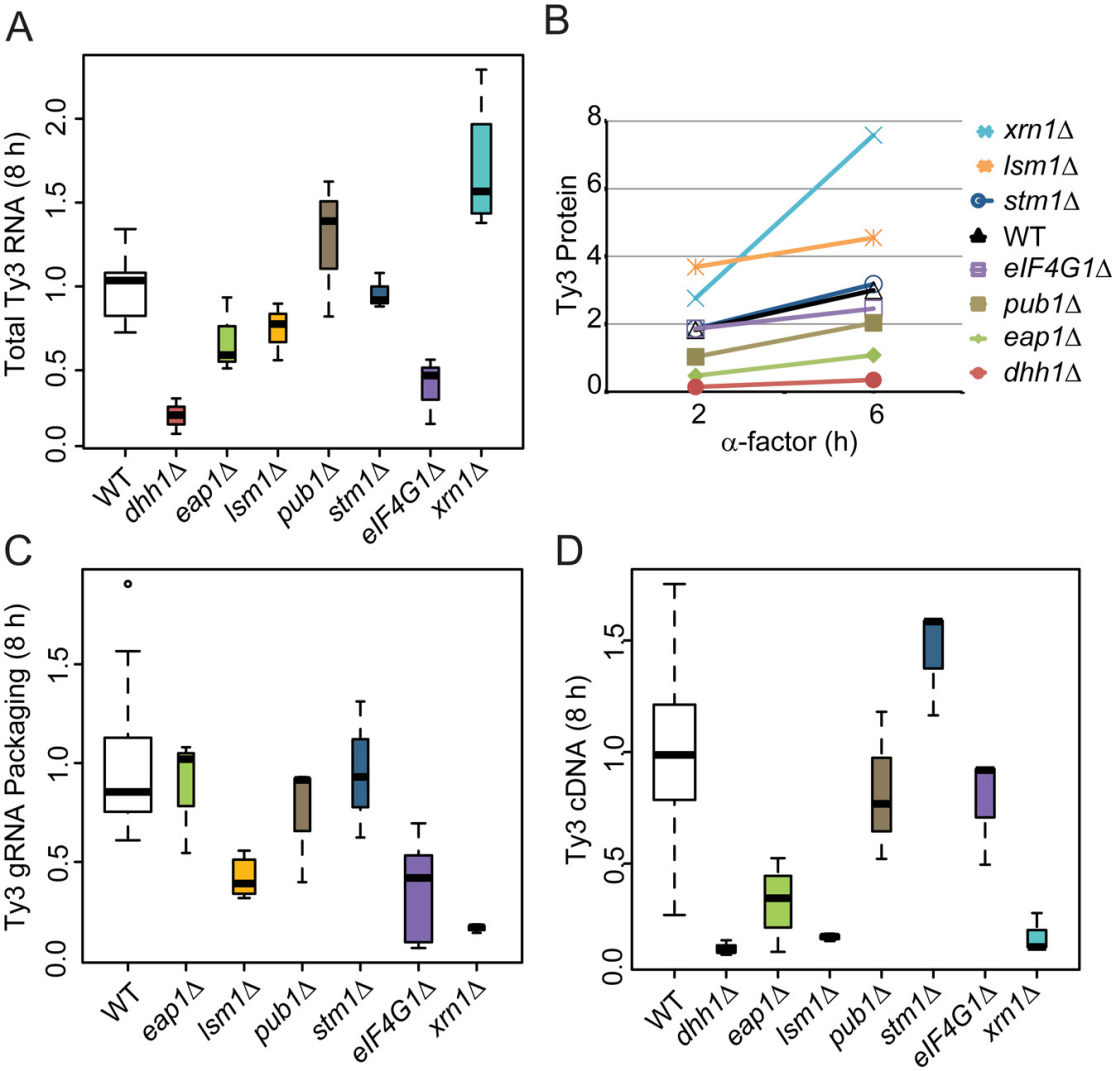
Strains deleted for PB host factors are defective at different stages. WT (BY4741) and deletion strains were induced with α-factor for 8 h **(A, C, and D)** and 0, 2 and 6 h **(B)**. Statistical significance was determined using the R statistical computing package (S1 Text, Supplemental Materials and Methods). **(A)** Total Ty3 RNA levels. RNA was isolated and quantified by northern blotting. WT Ty3 RNA signal was normalized to that of control *snR17a*. **(B)** Ty3 protein expression. Ty3 proteins were detected using α-CA. Pgk1 was detected with α-Pgk1 and used as a loading control. Data are representative of two or more independent inductions. Ty3 Gag3 protein levels were normalized to Pgk1 and averaged. **(C)** Ty3 RNA packaging. Native extracts were nuclease treated to degrade unpackaged RNA or left untreated. Ty3 RNA generated by *in vitro* transcription was added to the extract to monitor RNA digestion. Following treatment, RNA was purified and quantified by northern blotting. The percentage of Ty3 RNA resistant to degradation was calculated. **(D)** Ty3 cDNA synthesis. Cells were induced with α-factor for 8 h. DNA was isolated and digested with *Nhe*I and *Bam*HI restriction enzymes. Ty3 endogenous elements and cDNA were detected by Southern blot. Genomic *ARG4* was used as a loading control.

A significant subset of Ty3 Gag3-interacting factors affected Ty3 retrotransposition and assembly focus formation (Table 1). These were further investigated to understand the basis of their effects on retrotransposition. WT, *dhh1Δ*, *eap1Δ*, *lsm1Δ*, *pub1Δ*, *stm1Δ eIF4G1Δ* and *xrn1Δ* cells were induced with pheromone or left untreated and sampled at 2, 6 or 8 h after initiation of treatment. Samples representing each time point were examined for Ty3 RNA, protein, packaging, and cDNA retrotransposition intermediates. Full-length Ty3 and control *SNR17a* RNAs were measured by quantitative northern blot analysis of RNA extracted from cells induced for 8 h and normalized values were compared (Fig 6A and S3A Fig). *Dhh1Δ* and *eIF4G1Δ* cells had the lowest levels of Ty3 RNA; *eap1Δ* and *lsm1Δ* cells were modestly reduced; *stm1Δ* cells had no significant difference from WT; *pub1Δ* mutant strain was indeterminate, due to variability among independent samples; and *xrn1Δ* was significantly elevated for Ty3 RNA. These qualitative differences in Ty3 RNA levels among mutants that were reduced for Ty3 retrotransposition suggested that these factors might act at different points in the Ty3 replication cycle with Dhh1/DDX6 and eIF4G1 acting earliest, on the level of Ty3 RNA. Although the difference between *dhh1Δ* and WT was greatest at 8 h where WT expression was highest, even at 2 h, *dhh1Δ* strain was reduced in Ty3 RNA (S4A Fig).

Gag3 expression was measured by immunoblot analysis of Gag3 and CA-containing processing intermediates in extracts of cells collected at 0, 2 and 6 h. The Gag3 signal was normalized to loading control Pgk1 (Fig 6B and S3B Fig). Amounts of Gag3 were very low for *dhh1Δ* cells and reduced for *eap1Δ* cells. Eap1 is one of two eIF4E binding-proteins with a role in translation repression proposed to occur through competition with eIF4G, which binds to eIF4E during cap-dependent translation initiation [70]. Eap1 deletion would be predicted to positively affect both RNA and proteins levels, which is contrary to our observation. There was a significant decrease Ty3 RNA and protein that could account for the decrease in retrotransposition as the packaging assay indicated that RNA could be packaged with similar efficiency to WT. Low amounts of RNA correlated qualitatively with reduced Gag3. Levels of Ty3 Gag3 in *stm1Δ*, *pubΔ*, and *eIF4G1Δ* cells were not distinguishable from WT. Despite WT levels of RNA, amounts of Gag3 were modestly increased in *lsm1Δ* cells. Consistent with increased RNA levels, Gag3 levels were higher in the *xrn1Δ* cells than in WT yeast.

As Gag3 and Gag3-Pol3 assemble into VLPs with RNA, Ty3 PR is activated to process precursors into mature forms [59] and fails to process efficiently in assembly mutants [11, 12]. Although the extent of the phenotype was variable, the CA:Gag3 ratio was decreased in *xrn1Δ*, potentially indicating a deficiency either in assembly of RNA, Gag3 and Gag3-Pol3 into VLPs or in post-assembly processing. In addition to maturation of polyprotein precursors, increasing Ty3 gRNA resistance to nuclease digestion, as gRNA is packaged into VLPs serves as a measure of assembly [12]. In parallel with preceding experiments, cells were induced with pheromone for 8 h and native whole cell extracts were treated with nuclease or left untreated to evaluate RNA protection (Fig 6C and S3C Fig). *Lsm1Δ*, *eIF4G1Δ*, and *xrn1Δ* cells were significantly decreased for Ty3 RNA protection, indicating that RNA was not efficiently or correctly packaged.

Reverse transcription of gRNA into cDNA occurs subsequent to packaging, maturation of Ty3 RT, and recovery from G1 arrest [71]. CDNA was measured at 8 h by Southern blot analysis in which samples were normalized to a genomic fragment of *ARG4* (Fig 6D and S3D Fig). This assay showed that *dhh1Δ*, *eap1Δ*, *lsm1Δ and xrn1Δ* cells had reduced levels of Ty3 cDNA, consistent with reduced RNA and protein for mutants lacking Dhh1 and Eap1 but suggesting a later role for Lsm1 and Xrn1. Xrn1 and Lsm1 were implicated in restricting protein accumulation and promoting RNA protection. *Pub1Δ* cDNA levels were not significantly different than those of WT; *stm1Δ* cells had elevated cDNA, indicating that the defects in retrotransposition must occur at a later stage, such as nuclear entry or integration.

### Transition of Ty3 RNA from polysomes into PB is delayed in Xrn1-deficient cells

**Fig 7:**
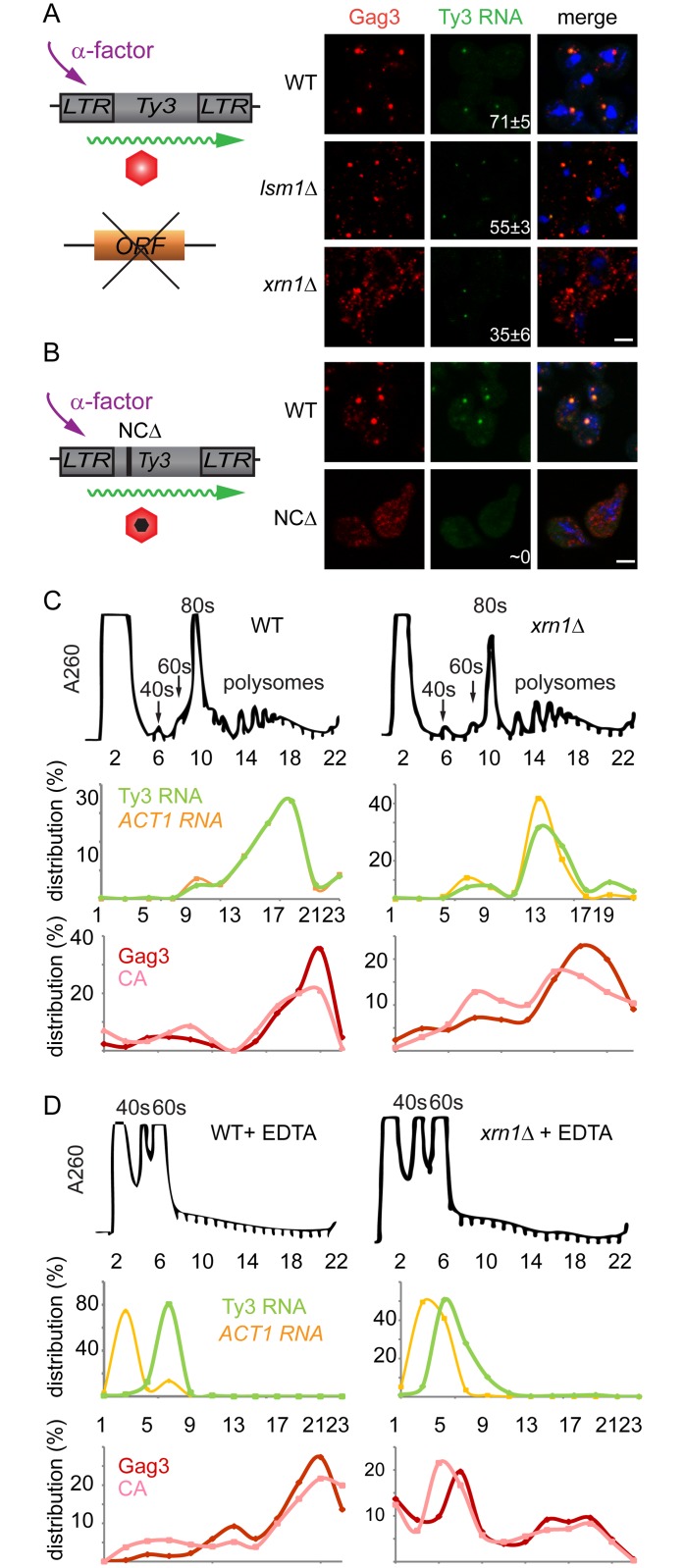
Xrn1 and Lsm1 are required for Gag3 foci formation in mating cells. **(A)** Localization of Ty3 Gag3 and RNA in WT (BY4741) and *lsm1Δ* or *xrn1Δ* strains. Cells were imaged by confocal microscopy after 4 h of α-factor induction. Ty3 Gag3 was detected by immunofluorescence using antibody against Ty3 VLPs and Ty3 RNA was detected by FISH using Ty3-specific oligonucleotides (S1 Text, Supporting Information Methods and Materials). Insert indicates % cells with RNA foci (mean ± SD). Scale bar = 2 μm. **(B)** Gag3-ΔNC fails to localize to PB foci. Ty3Δ (yVB1672) cells were transformed with plasmids expressing either WT Ty3 (pTD3685) or with Ty3 with deletion of the NC domain of Gag3 (NCΔ) (pPS3705) under the native promoter. Cells were imaged by confocal microscopy after 4 h of α-factor induction as described in **(A)**. Polysome analysis of WT (BY4741) or *xrn1Δ* cells induced with α-factor for 2 h. Cell lysates, either untreated **(C)** or treated **(D)** with EDTA, were analyzed by sucrose gradient sedimentation. The 40, 60 and 80s ribosomal subunits and polysomes were monitored with continuous A_260_ measurements. Total RNA was extracted from even number fractions and was analyzed by northern blot for Ty3 (green) and *ACT1* RNA (orange). Protein was extracted from odd number fractions and was analyzed by western blotting for Gag3 (red) and CA (pink). RNA and protein data is expressed as a percentage of the total in all fractions (distribution %). Data are representative of results from two independent experiments.

The *lsm1Δ* and *xrn1Δ* strains showed increased amounts of Gag3, but this contrasted with decreased processing in *xrn1Δ* cells and decreased packaging of Ty3 gRNA in both mutants. In addition, the Ty3-GFP reporter showed deficient focus formation in *lsm1Δ and xrn1Δ* cells (*lsm1Δ* = 15±8% and *xrn1Δ* = 38±5%) (Fig 3). These properties suggested a defect in transitioning the gRNA from translation into assembly phases or in assembly itself. Because of programmed frameshifting between *GAG3* and *POL3*, Gag3 is about 20-fold more abundant than Gag3-Pol3 [63]. To increase the sensitivity of analysis, cells were induced with pheromone for Ty3 expression for 8 h and Gag3 was analyzed by immunofluorescence using an anti-VLP antibody that reacts with Gag3 (see Materials and Methods). RNA was detected by fluorescence *in situ* hybridization (FISH) using three pooled oligonucleotide probes complementary to Ty3 internal regions. WT cells showed strong co-localization of Gag3 and Ty3 RNA in cytoplasmic foci (Fig 7A). Analysis of *lsm1Δ* and *xrn1Δ* strains showed a punctate cytoplasmic signal interspersed with fewer intense foci than in WT cells (*lsm1Δ* = 55±3% and *xrn1Δ* = 35±6%, compared to WT = 71±5%). However, the overall faintness of the RNA signal prevented a determination as to whether the Ty3 RNA in the mutants also formed fewer foci. RNA foci that were observed were co-localized with Gag3 puncta. These observations suggested that Gag3 retained interaction with Ty3 gRNA, but failed to coalesce normally into assembly foci. This interpretation was confirmed by comparison of this phenotype to that of a Ty3 Gag3-NCΔ mutant, lacking most of the NC-coding region so that it fails to interact with gRNA. This mutant failed to form Gag3 foci and did not have detectable co-localization of Gag3 and gRNA [11](Fig 7B). We conclude that despite an abundance of Ty3 RNA and Gag3, *lsm1Δ* and *xrn1Δ* strains fail to efficiently form Ty3-PB foci so that protein and RNA remain diffuse.

PB components are associated with both deadenylation and translational suppression, and the fluorescence experiment implicated Xrn1 and Lsm1 in localization of Ty3 RNA and Gag3 to foci. In addition, *lsm1Δ* and *xrn1Δ* cells had elevated levels of Gag3, suggesting defects in those PB functions. If Xrn1, deletions of which displayed the more severe phenotype with respect to protein localization and retrotransposition, were required to transition Ty3 RNA from polysomes into assembly, then Gag3 and Gag3-Pol3 polysomes in the mutant should persist relative to those in WT. To test this hypothesis, extracts from WT and *xrn1Δ* cells were fractionated by velocity sedimentation over sucrose density gradients. WT cells treated with pheromone for 2 h or left untreated and processed for polysome analysis did not show gross differences in the RNA A_260_ profile (S5A and S5B Fig). WT and *xrn1Δ* cells were induced with pheromone for 2 h and processed for polysome gradient analysis in the absence (Fig 7C) and presence (Fig 7D) of EDTA. As anticipated, EDTA dissociated ribosomes into 40 and 60S subunits. Alternate gradient fractions were processed for northern blot analysis using Ty3 and *ACT1* control probes and immunoblot analysis using anti-CA (Fig 7C and 7D). In the polysome gradients of WT and *xrn1Δ* extracts not treated with EDTA, Ty3 Gag3 and Ty3 RNA, were concentrated in fractions 17 to 21. In the WT and *xrn1Δ* EDTA-treated samples, most Ty3 and *ACT1* RNA shifted to the top of the gradient as expected for RNAs not associated with polysomes. For extracts from WT cells the pattern of Ty3 Gag3 and CA persisted in fractions 17 to 21 as expected for VLPs that are EDTA resistant (Fig 7D). However, in extracts from *xrn1Δ* cells, Gag3 and CA shifted to gradient fractions 6 and 7 and only a small amount of precursor Gag3 remained in the fraction normally associated with assembled VLPs (Fig 7D). These results indicated that compared to Ty3 protein in WT cells, most Ty3 protein in *xrn1Δ* mutant cells is in an EDTA-sensitive form, suggesting that Gag3 associates co-translationally with its RNA template; furthermore, this form accumulates in the *xrn1Δ* mutant, consistent with delayed transitioning into assembly.

## Discussion

Three important insights into the events between Ty3 retrotransposon transcription and VLP formation resulted from this investigation. First, activation of the mating MAP-kinase pathway stimulates formation of cytoplasmic foci that concentrate PB components together with retrotransposon RNA and protein. Second, PB components Dhh1/DDX6, and Lsm1 and Xrn1 are required for Ty3 expression and assembly of RNA into VLPs, respectively (see S7 Table, Comparison of Ty3 transposition frequency and retrosomes formation in mutant strains). Third, Ty3 expression enhances formation of mating PB foci in cells with attenuated cell cycle arrest. These findings raise questions about how PB proteins facilitate packaging of retrotransposon RNA into VLPs, how any RNA avoids degradation during sequestration in PB and whether retroelements enhance their own survival by promoting formation of perinuclear RNPs that facilitate nuclear entry or transfer during or after mating.

### Mating signals induce formation of RNP containing PB and Ty3 components

Analysis of RNP foci induced by the MAP-kinase mating pathway identified PB proteins Dhh1, Dcp2, Ded1, Lsm1, Xrn1, Pat1, and Edc3. Because Ty3 proteins formed only one or two foci per cell and individually co-localized with each of these PB proteins, we concluded that individual foci generally contain Ty3 proteins and all or most of these PB proteins. We previously established that induction of WT Ty3 expression under the *GAL1-10* UAS, causes formation of foci of PB proteins, but expression of Gag3 assembly mutants disrupts foci formation in various ways [6, 10–12]. These foci were designated retrosomes because of their apparent role in retrotransposon VLP assembly. Haploid cells undergoing pheromone-stimulated MAP kinase signaling were previously reported to form foci containing Dhh1/DDX6 and Dcp2 [16]. Because Ty3 is expressed during pheromone induction and because both PB and SG contain these components, we tested for Ty3 RNA and proteins and additional PB and SG components in these foci. Ty3 and PB components localized to these foci, but two SG proteins tested, Eap1 and Pub1, did not form microscopically visible foci (reviewed in [4, 19, 72, 73]). We concluded that mating cell retrosomes are more closely related to PB than to SG RNPs.

### Ty3 expression and VLP formation requires PB components Dhh1/DDX6, Lsm1 and Xrn1 at different stages

Dhh1/DDX6, Lsm1, Xrn1, and Pub1 were among the proteins required for WT levels of Ty3 retrotransposition. One of the most surprising aspects of Ty3 retrosomes is that Ty3 components including RNA assemble in association with factors implicated in RNA degradation. Further underscoring this unexpected relationship, in the absence of Dhh1, Ty3 RNA levels are dramatically reduced. In yeast, Dhh1/DDX6 is implicated in RNA expression, translation repression, tRNA retrograde translocation into the nucleus during starvation, and RNA turnover [21, 74–76]. However, because homologs of Dhh1 protect maternal RNAs in other organisms [21, 77], one attractive possibility is that Dhh1 shields Ty3 RNA from degradation. Another possibility is that Dhh1 affects Ty3 RNA levels through its role in promoting translation of Ste12 a downstream mating MAP kinase target transcription factor [16] or via its action in transcription elongation [78]. However, these last two roles in transcription seem inconsistent with the observation that expression under the heterologous *GAL1* promoter or expression of a truncated form lacking *POL3* do not rescue the low levels of Ty3 RNA in *dhh1Δ* cells (S4A and S4B Fig).

Ty3 RNA and Gag3 levels were modestly elevated in *xrn1Δ* compared to WT cells, which is consistent with absence of a major exonuclease [24]. However, polysome analysis showed that the *xrn1Δ* mutant lacked the high molecular weight, EDTA-resistant complexes associated with VLP formation in WT cells. Instead, upon EDTA treatment, Gag3 and RNA shifted upward in gradients, consistent with a greater proportion of Gag3 and Ty3 RNA in *xrn1Δ* cells remaining associated with polysomes rather than exiting translation for assembly. Although Gag3 levels were elevated and there were Gag3 foci in these cells, the pattern was relatively dispersed. Xrn1, has previously been implicated in co-translational degradation of polysomal RNAs indicating that at least a fraction is associated with polysomes [79], but our findings suggest that Xrn1 also interacts with RNAs that are not targeted for degradation. Xrn1 has been previously implicated in mRNA localization via microtubule trafficking [80]. One straightforward explanation for our results is that Xrn1 has a role in trafficking Ty3 RNA from polysomes to PB retrosomes. The *lsm1Δ* cells displayed a similar, although less severe, phenotype to that of the *xrn1Δ* cells. Other studies have suggested that the Lsm1-7 heptameric complex and Pat1 interact with the 3’ ends of RNAs and associate with other PB proteins, including decapping proteins and Dhh1 bound to the 5’ end (summarized in [1]). Based on these Lsm interactions and the mutant phenotype, we propose that Lsm1-7 also participates in releasing the translating Ty3 RNA from polysomes, thereby promoting its localization into PB retrosomes.

Unlike Dhh1, Lsm1 and Xrn1, poly(A) binding-protein Pub1 did not localize to PB retrosomes, and *pub1Δ* cells had enlarged Ty3 Gag3 foci. This result is consistent with a model in which the association of Pub1 with translating poly(A) RNAs acts in opposition to PB suppression of translation and subsequent sequestration of Ty3 RNA. While on the surface, the decrease in transposition observed in this mutant might appear to be at odds with larger retrosomes, these retrosomes might exceed an optimal size for assembly foci so that enmeshed VLPs are unable to access nuclear pores.

### Ty3 expression promotes mating-cell PB formation in arrest-attenuated *far1Δ* cells

Because Ty3 RNA and proteins associate with PB proteins in mating cells, we asked whether Ty3 expression promotes formation of mating cell PB. However, mating cell PB formed in a Ty3Δ strain, indicating that full-length Ty3 is not essential for mating PB formation. In mating, the MAP-kinase Fus3 phosphorylates Far1, which inhibits Cdc28-Cln thereby arresting cells in G1 [68]. *Far1Δ* cells, which fail to arrest, but initiate the mating transcriptional program, showed reduced PB formation compared to WT cells. This attenuated PB phenotype allowed us to further test for a role of Ty3 in PB formation.

Because mating cells undergo dramatic changes in transcription and translation, we postulated that PB formation during mating is associated with a specific role in the haploid to diploid transition. For example some cellular RNAs are known to exit and re-enter translation as cells transition from pheromone arrest to recovery [81, 82]. PB protein functions are known to affect mating, for example, PB factors Xrn1 and Dhh1, promote mating cell nuclear fusion and Ste12 expression, respectively [16, 83]. Although Ty3Δ cells formed mating PB, some observations indicate that Ty3 expression could contribute to mating PB formation. First, as previously reported, expression of Ty3 under the *GAL1* promoter in non-mating cells, causes formation of one or two large PB per cell [6]. Second, Ty3 expression increases PB formation in *far1Δ* cells, in which PB formation is attenuated. Third, Edc3 and Pat1, which provide structural support for PB formation in glucose-deprived cells [44–46], are dispensable for Ty3 Gag3 mating PB formation. In mating cell PB, Gag3 may compensate for the deficiency of Edc3 or Pat1 by acting as a scaffold for PB protein recruitment in a manner similar to the contribution observed for the prion-like domain of Lsm4 [44–46]. Finally, a role for persisting transcripts from almost forty solo Ty3 LTRs some of which are induced by pheromone [14] cannot be excluded by our experiments with Ty3Δ cells. Production and storage or degradation of such non-coding transcripts might promote PB formation in Ty3Δ cells. PB formation was recently correlated with larger daughter buds [69]. The tolerance of genomes for retrotransposons is still not completely understood. If Ty3 or its LTR transcripts do enhance mating PB formation, it might, over an evolutionary-scale timeframe contribute to Ty3 genomic retention.

### Ty1 and Ty3 coordinate dependence on PB host factors

Similar to the *S*. *cerevisiae* Ty3/Gypsy LTR retroelement described here, the Ty1/Copia LTR retroelement requires PB proteins for retrotransposition [84, 85]. However, there are significant differences in the lifestyles of these two elements and some of these likely affect their interactions with PB components. First, Ty1 transcripts are among the most abundant in haploid cells but Ty3 transcripts are present in relatively low amounts except under induction. Ty1 transcripts form poly(A) T body foci, which are discrete from PB [86]. Ty1 retrotransposition is low due to repressive transcriptional and post-transcriptional copy number control (CNC) [87–89]. In addition to the full-length Ty1 RNA, subgenomic anti-sense and sense transcripts with roles in CNC are produced. The former has been implicated in epigenetic repression of Ty1 transcription [88, 90], while the latter has recently been shown to encode a truncated Gag protein with both specific and non-specific nucleic acid binding activities that interfere with Ty1 VLP formation. The 5’ to 3’ exonuclease, Xrn1, negatively controls the half-lives of these subgenomic RNAs. In contrast to Ty1, full-length Ty3 copy number, transcription and retrotransposition are relatively low except under conditions of mating pheromone induction [14, 15, 67]. However, these observations do not exclude a similar role for Xrn1 in the Ty3 lifecycle to that in the Ty1 lifecycle. For example, Ty3 expresses a subgenomic transcript of about 3.1 kb which could support expression of truncated Gag3 species. In addition, a species that reacts with anti-CA antibodies, but which is smaller than CA is observed (S3B Fig). However, there do not appear to be relatively higher amounts of the subgenomic sense transcript and the shorter protein in the Xrn1 mutant. In addition, the nucleic acid binding activity for Ty3 upon which PB association of Ty3 RNA depends is lodged in a discrete Gag3-derived NC zinc knuckle species [11, 91] which is not found in Ty1. Overall, the low level of Ty3 copy number compared to that of Ty1 (2 versus 32 in BY4741 for example), and low level of RNA under non-inducing conditions suggests that post-transcriptional CNC if it exists for Ty3 in BY4741 is not as strong as for Ty1.

Second, Ty3 and Ty1 apparently differ in their mode of utilization of PB factors and in the fold-impact of PB factor loss. As discussed in this work and previous work, Ty3 retrosomes are coincident with PB. In contrast, Ty1 retrosomes form in association with the ER through which Gag proteins transit [92], and Ty1 retrosomes associate transiently with or overlap partially, but are not coincident with PB. Nonetheless, deletion of several genes encoding PB factors, also reduced Ty1 retrotransposition compared to that observed in WT cells [*xrn1Δ* (0.5%) < *dhh1Δ* (1.6%) < *upf1*, *3Δ* (2.5,3.4%) < *dcp2Δ* (5.7%), *lsm1Δ* (10%) < *pat1Δ* (16%) < *ccr4Δ* (20%) < *edc3*, *2Δ* (50,86%)] (for example, [85]). Loss of these PB factors generally had the greatest effect at a step between Gag production and cDNA levels [84, 85]. Consistent with this defect, and similar to what was observed for Ty3, the *xrn1Δ* strain failed to package genomic RNA [85], and *xrn1Δ*, *lsm1Δ*, and *pat1Δ* showed reduced RNA foci [84]. Ty3 and Ty1 differ qualitatively and quantitatively with respect to dependence upon PB factors. For example, *DHH1* deletion causes almost complete loss of Ty3, but not Ty1 transcripts, and deletion of several PB genes, for example *PAT1*, affected Ty1, but not Ty3 retrotransposition. In addition, under pheromone induction conditions utilized in our experiments, Ty3 retrotransposition frequency was several orders of magnitude greater than that of native Ty1 retrotransposition and with the exception of *dhh1Δ* and *xrn1Δ*, the Ty3 frequency was much less sensitive to loss of PB function than was that of Ty1 perhaps explaining some of the apparent differences in factor requirements.

The observation that Ty1 and Ty3 expression in response to MAP-kinase activation [55] and common PB factors [7, 54, 84, 85, 93], suggests that they could be in an evolutionary arms race with each other as well as their host. However, further considerations show distinctions. For example, as described here, Ty3 responds to mating MAP-kinase signaling which further destabilizes Ty1 protein [94, 95]. In diploid cells, where Ty3 is repressed, nutritional deprivation MAP-kinase signaling activates Tec1/Ste12 induction of Ty1 expression and retrotransposition [96, 97]. Together, these observations suggest that that despite shared host factors, Ty1 and Ty3 have privatized cellular programs within which they retrotranspose, thus potentially minimizing direct competition.

### Plant and animal viruses display diverse interactions with PB components

Comparison of the roles of PB proteins supporting Ty1 and Ty3 replication in yeast to PB roles in the replication of viruses and retroelements in other systems produces a mixed picture. The plant positive-strand RNA virus, Brome Mosaic Virus, depends upon PB proteins for replication in a yeast model [98]. DEAD box helicase Dhh1/DDX6 was implicated in formation of HIV intracellular intermediates in human immunodeficiency virus (HIV) core assembly and in prototypic foamy virus RNA packaging [99, 100]. However, other studies report no correlation between virus assembly and PB formation or DDX6 activity [35]. In human, DDX3 is required for WT nuclear export of HIV-1 RNA [101]. In our study, PB protein Ded1/DDX3 was identified by mass spectrometry, and co-localizes with Ty3 Gag3 in pheromone-treated cells, but its role remains to be tested. Although not identified in this study, several PB components are also classified in animal cells as restriction factors for retroviruses and retroelements [37, 38]. Collectively, these observations show that highly-conserved PB components associate with retroelement proteins and/or RNAs and may be used in different manners by retroviruses and retroelements.

### Parallels between retrotransposons and ancient perinuclear RNPs

Ty3 [102] and Ty1 [103] retrosomes associate with the nuclear envelope. The concentration of retrotransposons in perinuclear RNPs has obvious advantages for retrotransposition. Accumulating Gag associated with perinuclear clusters would have access to newly-exported gRNAs for packaging. Intriguingly, PB factors Dhh1 and Pat1 are required in parallel pathways for retrograde transport of tRNAs into the nucleus in nutritionally starved cells [75]. Multiple steps occur during VLP assembly and PB factors may promote more than one aspect of this process, including tRNA capture. Indeed, although its relationship to retrograde tRNA transport is not clear, tRNA association has been shown to promote nuclear entry of HIV cores and cDNA [104].

It is noteworthy that Ty3 activation in mating yeast displays many parallel features to retrotransposon activation in germ cell lineages of higher organisms. Animal retroelements are transcriptionally repressed in somatic tissues by epigenetic mechanisms. Repression is relieved in germ cell lineages by transient de-methylation. During phases of transcriptional activation, retrotransposition is suppressed by post-transcriptional degradation of repeated sequence/retroelement RNAs in specialized perinuclear RNP granules. In *D*. *melanogaster*, *C*. *elegans* and *M*. *musculus*, these RNPs contain PIWI Argonaute proteins, piRNAs, and PB components DEAD box helicases DDX3 (ScDed1) and DDX6 (ScDhh1), decapping enzyme subunits Dcp1 and Dcp2, and 5’ to 3’ exonuclease Xrn1 [reviewed in [40, 42, 105]]. Germ cell granule suppressors of retroelements (e.g. APOBEC3, RNAi Argonaute and Dicer components of PB) are not endogenous to budding yeast. However when expressed, two of these factors, APOBEC3G [85] and heterologous yeast Argo/Dicer, suppress Ty1 retrotransposition [43]. This suggests that localization of retroelement RNAs to these RNPs is conserved.

### Summary

We show here that yeast cells undergoing mating MAP-kinase signaling form PB containing Ty3 RNA and proteins, and that components of yeast mating cell PB promote Ty3 retrotransposition at multiple stages. We speculate that in animal germ cells retrotransposition might also be promoted by PB functions associated with perinuclear granules, but is suppressed by components of RNAi. This work poses the following questions for future investigations: Is MAP kinase pathway signaling the underlying regulator of PB formation? How are RNAs recognized for inclusion in mating cell PB, but protected from degradation? In animal cells when suppression of retroelements in germ cell lineages is incomplete do PB components promote the spread of elements into new genomes?

## Materials and Methods

### Strains, plasmids and growth conditions

Yeast and bacterial culture methods were as previously described [106, 107] except where noted. Bacterial strains HB101 or DH5α were used for plasmid preparation. All *S*. *cerevisiae* strains were derivatives of BY4741 (*MAT* **a** *his3Δ1 leu2Δ0 met15Δ0 ura3Δ0*). (S1 Table). BY4741 derivatives included strains deleted for Ty3 elements and killer RNA, and strains tagged with fluorescent reporter proteins. Manipulation of strains and plasmids utilized standard molecular genetics. For all α-factor induction, yeast cultures were grown at 24°C to OD_600_ of 0.2 and α-factor was added to 6 μM final concentration. A complete description of procedures is provided (S1 Text, Supplemental Materials and Methods; S2 Text, Plasmid construction and sequences; and S2 Table, Primers used in this study and S3 Table Plasmids used in this study.

### Antibodies

Polyclonal rabbit antibodies (Berkeley Antibody Company) were raised against WT Ty3 VLPs purified as previously described [57] and affinity purified using recombinant Gag3 (this work). Polyclonal rabbit antibodies against Ty3 CA [60] were used for immunoblot analysis and immunofluorescence. A complete description is provided (S1 Text, Supplemental Materials and Methods).

### Mass spectrometry

Gag3-associated proteins were purified by α-VLP and control IgG affinity chromatography of whole cell extracts. Co-purifying proteins were digested with LysC and trypsin as described [108]. Peptides were analyzed by 1DLC-MS/MS using LTQ-Orbitrap XL MS (ThermoElectron) as described [108]. Database searching was performed using Batch-Tag and search Compare within the developmental version (v.5.2.2) of Protein Prospector as described. Proteins were identified by at least two peptides with a false-positive rate of 0.1%. A total of 154 proteins were identified of which 106 are not essential (S4 Table). A complete description is provided (S1 Text, Supplemental Materials and Methods).

### GO and network analyses

GO term enrichment analysis was performed using the Gene Ontology Term Finder (http://www.yeastgenome.org/cgi-bin/GO/goTermFinder.pl) and the SGD Gene Ontology Slim Mapper(http://www.yeastgenome.org/cgi-bin/GO/goSlimMapper.pl). Genes were sorted according to biological process, molecular function, and cellular component. For Gene Ontology Term Finder, threshold P-value of 0.01 were used to identify specific enrichment.

Gag3-associated RNA interaction network was visualized by Cytoscape [109].

Gag3-interacting P-body proteins (boxes) were queried for protein-protein physical interactions using SGD:Yeastmine (http://yeastmine.yeastgenome.org/yeastmine/begin.do). Cytoscape v3.1.0 (http://www.cytoscape.org/) was used to graphically map interactions (S1 Fig). Additional details including gene names of all interacting partners used to create the network map (S6 Table).

### Transposition assay

BY4741 or derivatives were transformed with a plasmid expressing Ty3 marked with a *HIS3* gene inactivated by the presence of an antisense artificial intron (Ty3-*his3*AI) [62, 110] under the native promoter (pDM3193). Strains were grown in SD-ura at 24°C to OD_600_ = 0.2, induced with α-factor and plated on synthetic dextrose medium (SD)-his, to select for cells that had acquired a chromosomal copy of Ty3-HIS3 in which the artificial intron was spliced out, and plated onto rich medium (1% yeast extract, 2% peptone, 2% dextrose) for total cell counts. At least four independent transformants were assayed for each strain. Transposition frequency was calculated as the frequency of His^+^ colonies and *p* values determined using the student t-test.

### Microscopy

Cells were grown at 24°C to OD_600_ = 0.2 and induced with 6 μM α-factor (GenScript USA Inc.) for 4 h or left uninduced as control. Fluorescent images of live cells were visualized using either a Zeiss Axioplan2 or a Zeiss LSM510 META inverted confocal scanning Plan-Apochromat fluorescence microscope. FISH was performed with an adaptation of previous methods [84, 111, 112]. Ty3 mRNA was detected with an equal parts mixture of antisense oligonucleotides complementary to coding sequence in Ty3 Gag3, RT, and IN. Ty3 protein was visualized using α-VLP followed by Alexa-fluor 568-conjugated donkey anti-rabbit IgG (Life Technologies). Cells were stained with 3 μg/ml DAPI (4’, 6-diamidino-2-phenylindole). FISH was imaged using a Zeiss LSM780 Plan-Apochromat inverted confocal laser scanning microscope. Images were processed for publication with Adobe Photoshop CS3 (Adobe Systems Inc.). For all experiments, images from at least three replicate samples consisting of at least 100 cells each were analyzed, and single plane images are shown. A complete description is provided (S1 Text, Supplemental Methods and Materials).

### Quantitative mating assay

Quantitative mating assays were done essentially as described [113]. In general: Cell cultures were grown at 23°C to OD_600_ = 0.2–0.3 and 10^6^ cells of each mating type were mixed, collected onto 25mm-GF/C filter disc (Whatman), and incubated at 30°C for the indicated times. The cells were eluted from the filter in 1 ml medium. For mating efficiency, 100 μl was plated on medium selective for complementation of auxotrophic markers in *MAT* **a** and *MATα* cells upon cell fusion. To determine the number of unmated cells, eluted cells were diluted 1–1,000 and plated onto the appropriate medium. Cells were incubated at 30°C for several days and colonies were counted. Mating efficiency (%) was calculated as the ratio of diploid cells to unmated cells. For specific experiments: For mating of Ty3(WT) BY4741 (*MAT* **a**) x BY4742(*MATα*) and Ty3Δ yVB1913 (*MAT* **a**) x yDM1456(*MATα*), cells were grown in YPD medium, mated on YPD medium for 1.5 and 2.5 h. Cells were eluted in SD medium lacking methionine and lysine, plated the same medium for mated cells, and on SD lacking methionine or lysine for unmated cells. For the effect of high level of Ty3 expression on mating, Ty3Δ strains yVB1672 (*MAT* **a**) and yVB1926 (*MATα*) were transformed with either a high-copy-number plasmid containing Ty3 (pDM3194) or a plasmid control (pYES2.0). Cells were grown in SD medium lacking uracil, mated on the same medium for 2.0 or 3.5 h. Cells were eluted in SD medium lacking histidine and methionine and plated on the same medium for mated cells, and on SD lacking histidine or methionine for unmated cells.

### VLP intermediate and polysome analysis

RNA, protein and packaging, and cDNA levels were measured essentially as described [10]. Statistical significance was determined using the program R [114] to perform one way ANOVA, with planned contrast between WT and individual mutant samples compared.

For polysome profilings, cells were grown at 24°C in YPD to OD_600_ = 0.2, induced with 6 μM α-factor (GenScript USA Inc.) for 2 h. Polysomes were prepared essentially as described [115]. Extracts from 10–12 OD_260_ cells were fractionated on a 4–47% linear sucrose gradient. A complete description is provided (S1 Text, Supplemental Materials and Methods).

## Supporting Information

S1 Fig Gag3-associated RNA interaction network visualized by Cytoscape. The coloring of edges corresponds to the specific PB protein that identified it as a physically interacting protein. Gag3-associated proteins (black) were included in the map; Ty3 transposition suppressors (triangles); Ty3 transposition enhancers (down arrows); no transposition phenotype or not tested (black dots). S6 Table contains additional details including gene names of all interacting partners used to create the network map.
(TIF)

S2 Fig Reduced transposition frequency in *lsm1Δ*, *pub1Δ* and *xrn1Δ* is not explained by difference in splicing efficiency of the *his3AI* tag. Relative splicing efficiency of the intron in Ty3-*his3AI* was determined by northern blot analysis of RNA from α-factor-treated WT and mutant strains using probes specific for total Ty3 RNA (*his3*), unspliced RNA (*AI* intron) and *SNR17a* (control), and normalized to WT (S1 Text). Data are representative results from at least two independent experiments.
(TIF)

S3 Fig Strains deleted for PB proteins are defective in retrotransposition. Data is support of Fig 6. Levels of Ty3 RNA **(A)**, protein **(B)**, gRNA packaging **(C)** and cDNA **(D)** were determined in untreated cells and cells treated with α-factor as indicated. Results of mutant cells were compared to WT (BY4741). The loading controls were *SNR17a* **(A)**, Pgk1 **(B)**, and *ARG4* **(D)**.
(TIF)

S4 Fig Ty3 RNA and protein expression in *dhh1Δ* are reduced after induction. **(A)** Ty3 RNA level is reduced in *dhh1Δ* cells after α-factor induction (2 h) compared to control RNA (*SNR17a*) and normalized to WT. **(B)** Reduction of Ty3 protein levels in *dhh1Δ* is independent of transcript length. Cells containing galactose-inducible Ty3 variants were expressed in WT and *dhh1Δ* cells for 6 h (S3 Table). Ty3 protein levels were monitored by anti-CA antibody. The loading control was Pgk1 (S1 Text).
(TIF)

S5 Fig Pheromone-treated and -untreated cells have similar polysome profiles. **(A)** Polysome analysis of WT (BY4641) cells. Cell lysates were analyzed by sucrose gradient sedimentation. The 40, 60 and 80s ribosomal subunits and polysomes were monitored with continuous A_260_ measurements. **(B)** and **(C)** Polysome analysis of WT cells treated with α-factor for 2 h. Cell lysates were analyzed as in **(A)** and were either untreated **(B)** or treated with EDTA **(C)**. Data are representative results from at least two independent experiments.
(TIF)

S6 Fig *S*. *cerevisiae* strain (BY4741) cured of killer RNA. Level of L-A and L-BC RNA in killer(+) and killer(-) strain in comparison to loading control.
(TIF)

S1 Table *S*. *cerevisiae* strains used in this study. Table listing *S*. *cerevisiae* strains used in this study.
(DOCX)

S2 Table Primers used in this study. Table listing primers used in this study.
(DOCX)

S3 Table Plasmids used in this study. Table listing plasmids used in this study.
(DOCX)

S4 Table MS data and Ty3 transposition in strains deleted for genes encoding proteins identified by MS/MS. (XLSX)

S5 Table GO analyses of Ty3 Gag3 interacting proteins. (XLSX)

S6 Table Network analyses of Ty3 Gag3 interacting proteins. GAG3-interacting PB proteins queried for protein-protein physical interactions using SGD:Yeastmine (http://yeastmine.yeastgenome.org/yeastmine/begin.do). Data in support of S1 Fig. Gene names of all interacting partners used to create the network map.
(XLSX)

S7 Table Comparison of Ty3 transposition frequency and retrosomes formation in mutant strains. Table showing comparison of Ty3 transposition frequency and retrosome formation in mutant strains.
(DOCX)

S1 Text Supplemental Materials and Methods. (DOCX)

S2 Text Plasmid construction and sequences. (DOCX)
