# Supplementary figures and images for: Ty3 Retrotransposon Hijacks Mating Yeast RNA Processing Bodies to Infect New Genomes
Source: PLoS Genet. 2015 Sep 30;11(9):e1005528. doi: 10.1371/journal.pgen.1005528 (PMC4589538; doi:10.1371/journal.pgen.1005528)

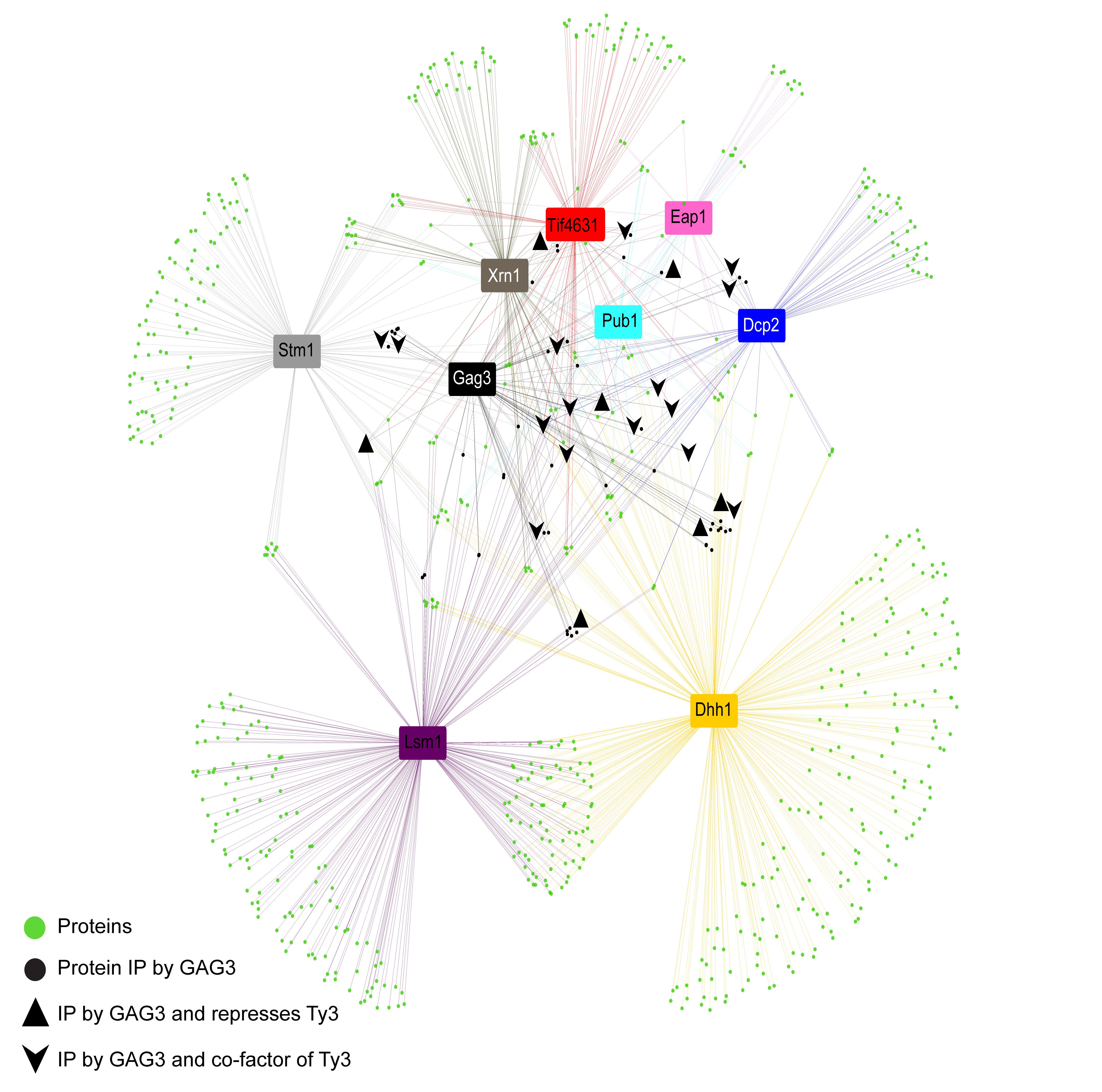

Supplement: S1 Fig — The coloring of edges corresponds to the specific PB protein that identified it as a physically interacting protein. Gag3-associated proteins (black) were included in the map; Ty3 transposition suppressors (triangles); Ty3 transposition enhancers (down arrows); no transposition phenotype or not tested (black dots). S6 Table contains additional details including gene names of all interacting partners used to create the network map. (TIF) [file pgen.1005528.s001.tif]

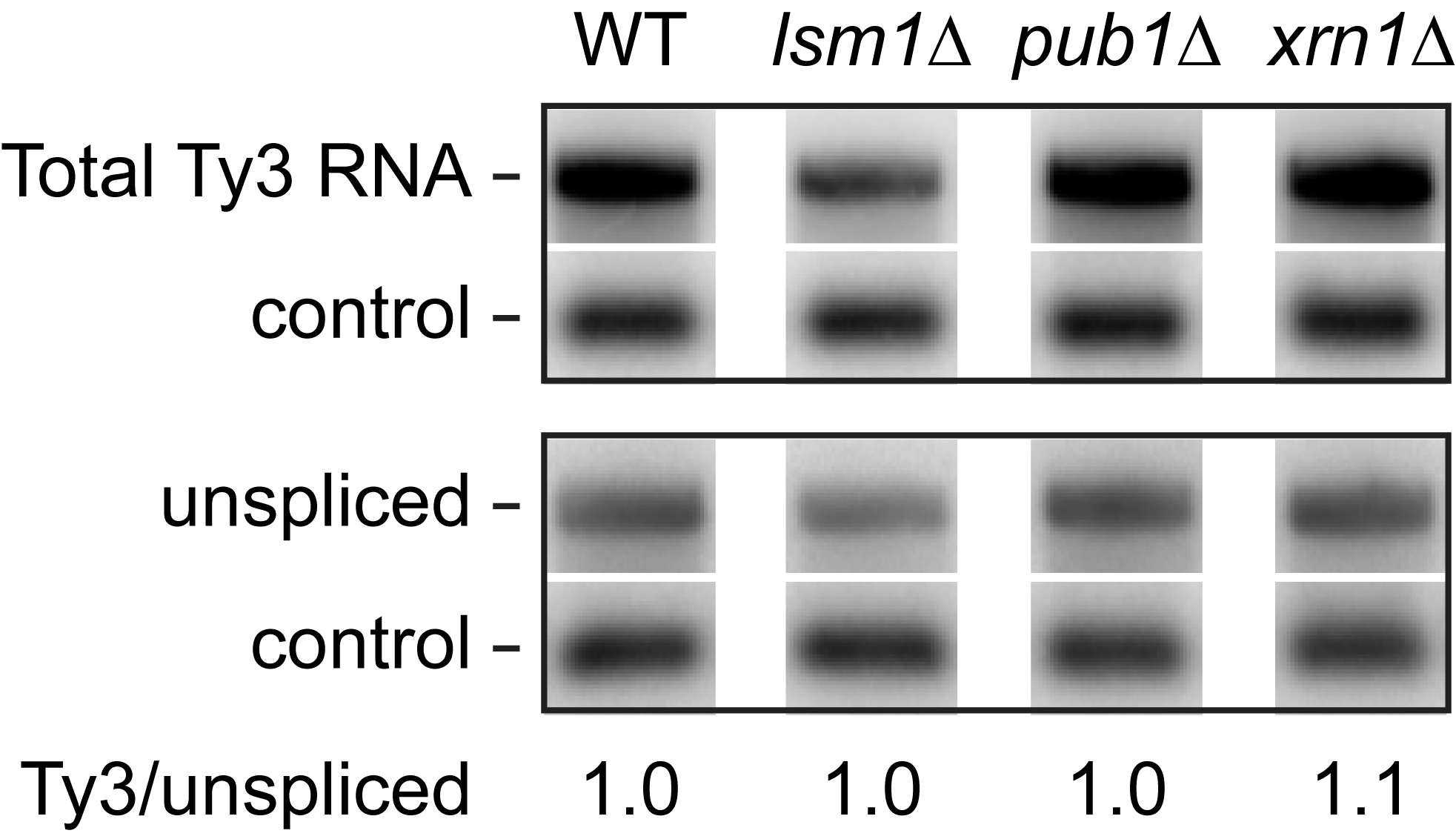

Supplement: S2 Fig — Relative splicing efficiency of the intron in Ty3-his3AI was determined by northern blot analysis of RNA from α-factor-treated WT and mutant strains using probes specific for total Ty3 RNA (his3), unspliced RNA (AI intron) and SNR17a (control), and normalized to WT (S1 Text). Data are representative results from at least two independent experiments. (TIF) [file pgen.1005528.s002.tif]

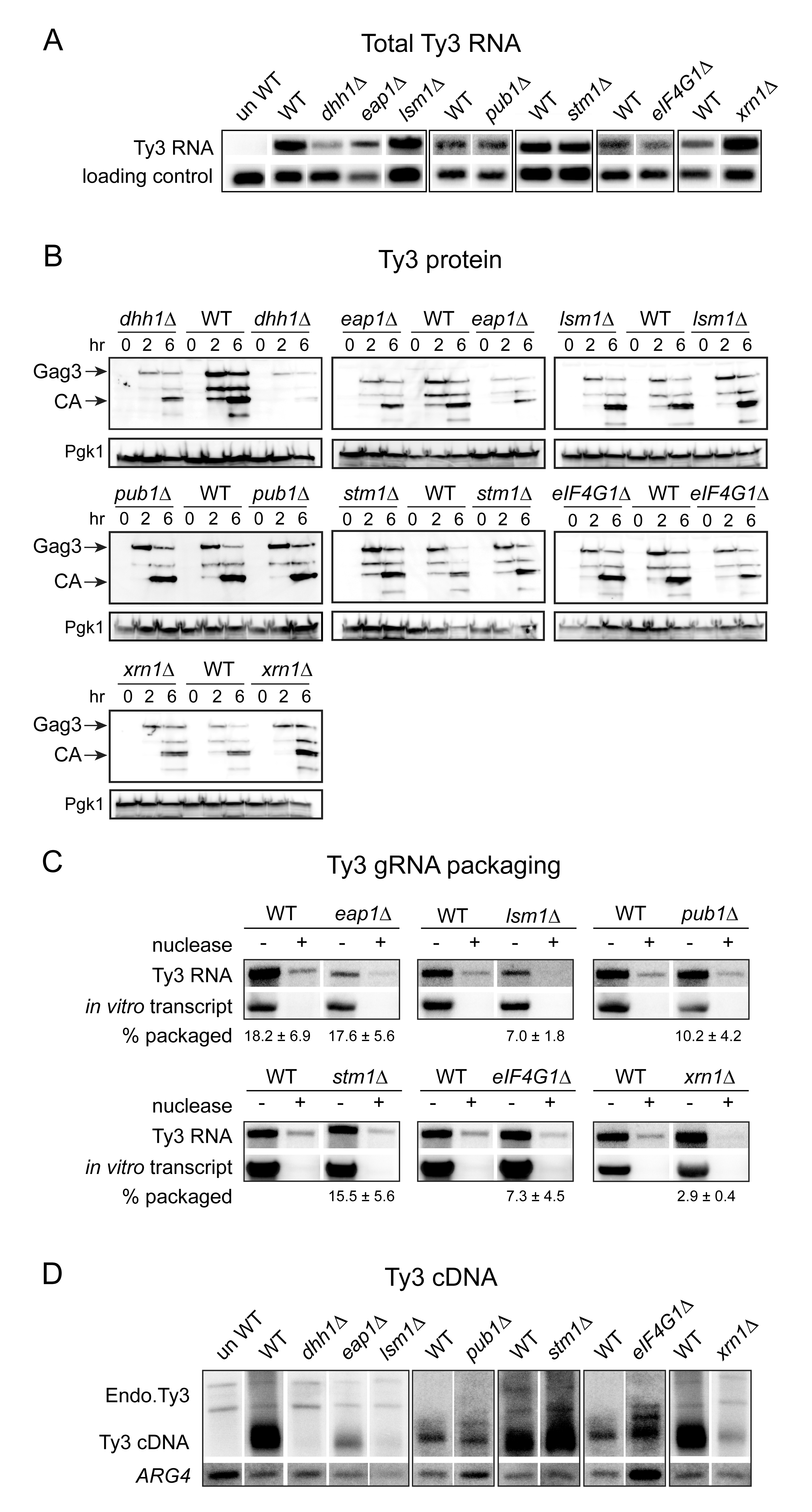

Supplement: S3 Fig — Data is support of Fig 6. Levels of Ty3 RNA (A), protein (B), gRNA packaging (C) and cDNA (D) were determined in untreated cells and cells treated with α-factor as indicated. Results of mutant cells were compared to WT (BY4741). The loading controls were SNR17a (A), Pgk1 (B), and ARG4 (D). (TIF) [file pgen.1005528.s003.tif]

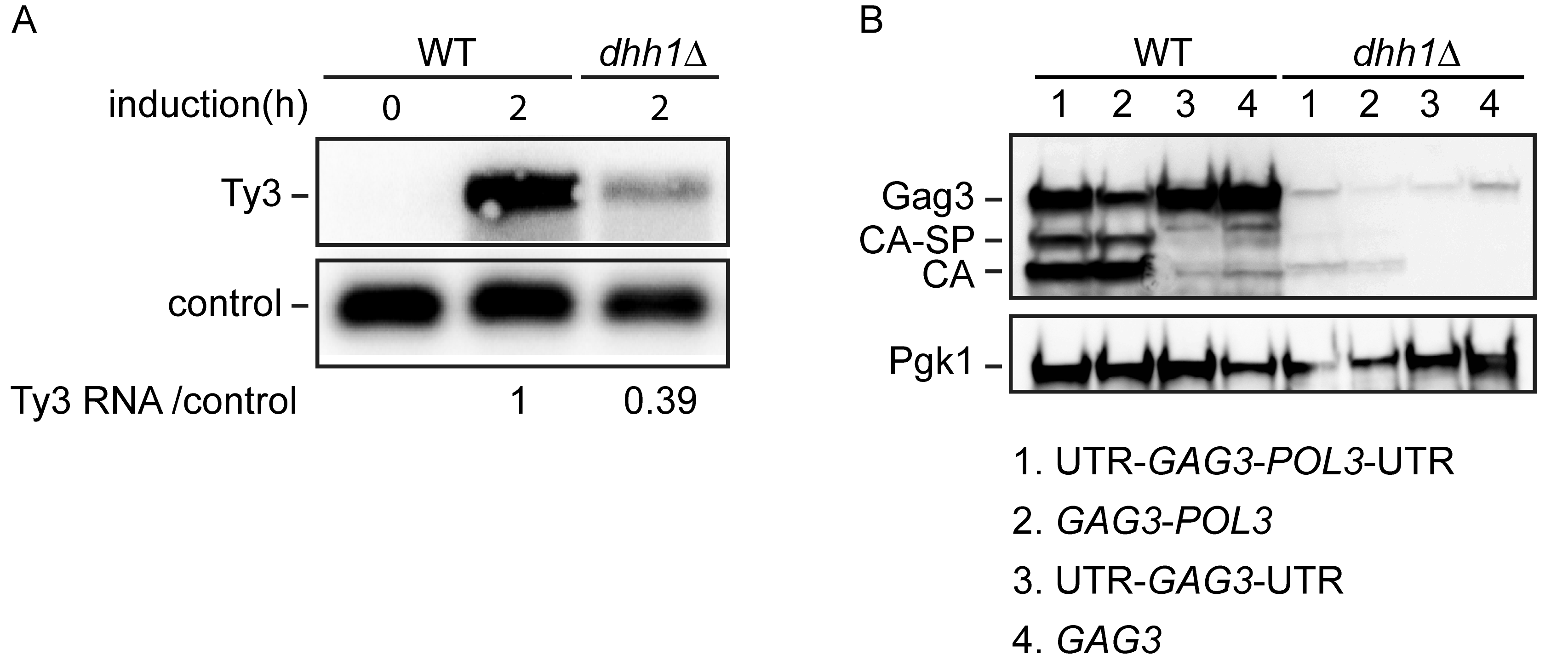

Supplement: S4 Fig — (A) Ty3 RNA level is reduced in dhh1Δ cells after α-factor induction (2 h) compared to control RNA (SNR17a) and normalized to WT. (B) Reduction of Ty3 protein levels in dhh1Δ is independent of transcript length. Cells containing galactose-inducible Ty3 variants were expressed in WT and dhh1Δ cells for 6 h (S3 Table). Ty3 protein levels were monitored by anti-CA antibody. The loading control was Pgk1 (S1 Text). (TIF) [file pgen.1005528.s004.tif]

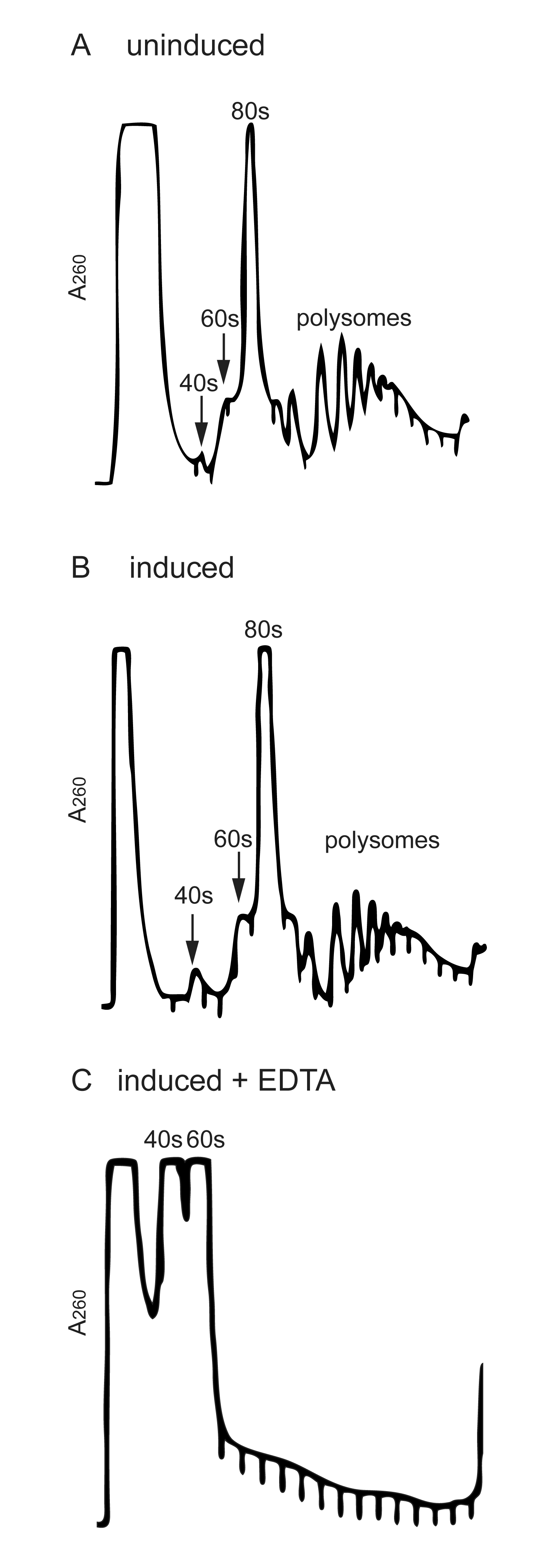

Supplement: S5 Fig — (A) Polysome analysis of WT (BY4641) cells. Cell lysates were analyzed by sucrose gradient sedimentation. The 40, 60 and 80s ribosomal subunits and polysomes were monitored with continuous A260 measurements. (B) and (C) Polysome analysis of WT cells treated with α-factor for 2 h. Cell lysates were analyzed as in (A) and were either untreated (B) or treated with EDTA (C). Data are representative results from at least two independent experiments. (TIF) [file pgen.1005528.s005.tif]

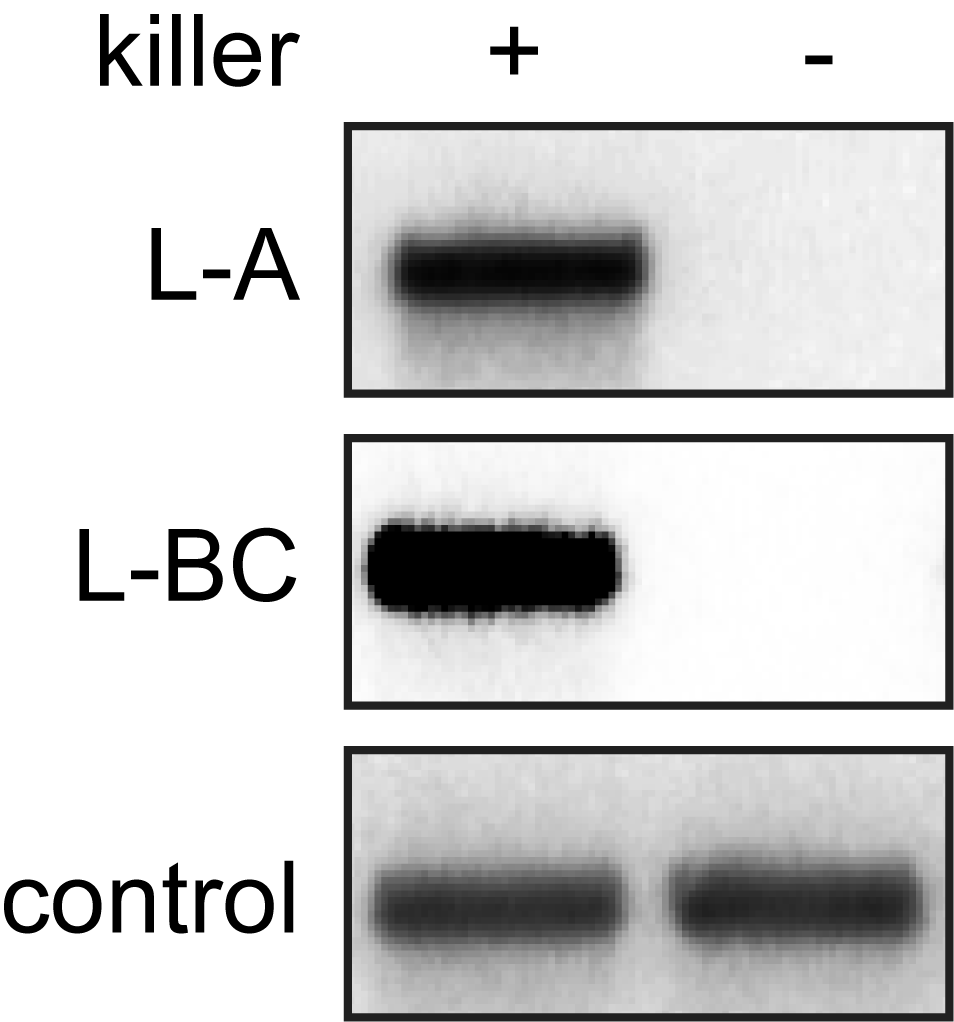

Supplement: S6 Fig — Level of L-A and L-BC RNA in killer(+) and killer(-) strain in comparison to loading control. (TIF) [file pgen.1005528.s006.tif]
