# Supplementary material for: Ty3 Retrotransposon Hijacks Mating Yeast RNA Processing Bodies to Infect New Genomes
Source: PLoS Genet. 2015 Sep 30;11(9):e1005528. doi: 10.1371/journal.pgen.1005528 (PMC4589538; doi:10.1371/journal.pgen.1005528)
Supplement: S1 Table — Table listing S. cerevisiae strains used in this study. (DOCX) [file pgen.1005528.s007.docx]

| **S1 Table.** *S. cerevisiae* strains used in this study. | | | |
| --- | --- | --- | --- |
| **Strain** | **Name** | **Genotype** | **Source / reference** |
| WT | BY4741 | *MAT***a** *his3*∆*1 leu2*∆*0 met15*∆*0 ura3*∆*0* | Open Biosystems [[1](#_ENREF_1)] |
| WT | BY4742 | *MATα his3*Δ*1 leu2*Δ*0 lys2*Δ*0 ura3*Δ*0* | Open Biosystems [[1](#_ENREF_1)] |
| WT Ty3(Δ) | yDM1456 | *MATα his3*Δ*1 leu2*Δ*0 lys2*Δ*0 ura3*Δ*0 ygrwty3-1*Δ*::URA3 yilwty3-1*Δ*::LEU2* (BY4741-derived) | This work |
| WT Ty3(Δ) | yVB1672 | BY4741 *ygrwty3-1∆::loxP* *yilwty3-1∆::loxP* | [2] |
| WT Ty3(Δ) | yVB1913 | BY4741 *ygrwty3-1*Δ*::URA3 yilwty3-1*Δ*::LEU2* | This work |
| WT Ty3(Δ) | yVB1926 | *MAT*α *leu2*∆*0 lys2*Δ*0 met15*∆*0 ura3*∆*0 ygrwty3-1∆::loxP* *yilwty3-1∆::loxP* (BY4741-derived) | This work |
| WT killer(-) | yVB1586 | BY4741 L-A(-) L-BC(-) | This work |
| *dcp2*Δ | yKC1846 | BY4741 *dcp2*∆::*kanMX* | This work |
| *DCP2-GFP* |  | BY4741 *DCP2-GFP(HIS3)* | [[2](#_ENREF_2)] |
| *DED1-GFP* | yVB1697 | BY4741 *DED1-GFP(kanMX)* | This work |
| *dhh1∆* | yNB1518 | BY4741 *dhh1*∆::*hphMX* | [[3](#_ENREF_3)] |
| *DHH1-GFP* |  | BY4741 *DHH1-GFP(HIS3)* | [[2](#_ENREF_2)] |
| *DHH1-GFP far1*Δ | yVB1829 | BY4741 *DHH1-GFP(loxP-LEU2-loxP)*  *far1*Δ *kanMX* | This work |
| *DHH1-GFP* Ty3(Δ) | yVB1777 | BY4741 *DHH1-GFP(kanMX) ygrwty3-1∆::loxP* *yilwty3-1∆:: loxP* | This work |
| *DHH1-GFP* Ty3(Δ) | yVB1830 | BY4741 *DHH1-GFP(loxP-LEU2-loxP) ygrwty3-1∆::loxP* *yilwty3-1∆::loxP* | This work |
| *DHH1-GFP far1*Δ Ty3(Δ) | yVB1831 | BY4741 *DHH1-GFP(loxP-LEU2-loxP) far1*Δ*::kanMX ygrwty3-1∆::loxP* *yilwty3-1∆::loxP* | This work |
| *eap1∆* | yVB1808 | BY4741 *eap1*∆::*kanMX* | This work |
| *EAP1-GFP* |  | BY4741 *EAP1-GFP(HIS3)* | Invitrogen |
| *edc3∆* |  | BY4741 *edc3∆*::*kanMX* | Open Biosystems [[1](#_ENREF_1)] |
| *EDC3-GFP* |  | BY4741 *EDC3-GFP(HIS3)* | [[2](#_ENREF_2)] |
| *far1*Δ |  | BY4741 *far1*Δ*::kanMX* | [[2](#_ENREF_2)] |
| *lsm1∆* | yVB1811 | BY4741 *lsm1∆*::*kanMx* | This work |
| *LSM1-GFP* |  | BY4741 *LSM1-GFP(HIS3)* | [[2](#_ENREF_2)] |
| *pat1∆* | yVB1819 | BY4741 *pat1∆*::*kanMX* | This work |
| *PAT1-GFP* |  | BY4741 *DCP2-GFP(HIS3)* | [[2](#_ENREF_2)] |
| *pbp1∆* |  | BY4741 *pbp1∆*::*kanMX* | Open Biosystems [[1](#_ENREF_1)] |
| *pbp4∆* |  | BY4741 *pbp4∆:*:k*anMX* | Open Biosystems [[1](#_ENREF_1)] |
| *pub1∆* | yVB1813 | BY4741 *pub1∆*::*kanMX* | This work |
| *PUB1-GFP* | yVB1696 | BY4741 *PUB1-GFP(kanMX)* | This work |
| *sbp1∆* |  | BY4741 *sbp1∆*:: *kanMX* | Open Biosystems [[1](#_ENREF_1)] |
| *stm1∆* |  | BY4741 (YLR150W) *stm1∆*::*kanMX* | Open Biosystems [[1](#_ENREF_1)] |
| *STM1-GFP* | yVB1771 | BY4741 *STM1-GFP(kanMX)* | This work |
| *tif4631∆* | yVB1812 | BY4741 *tif4631∆*::*kanMX* | This work |
| *TIF4631-GFP* | yVB1700 | BY4741 *TIF4631-GFP(kanMX)* | This work |
| *tif4632∆* |  | BY4741 *tif4632∆*::*kanMX* | Open Biosystems [[1](#_ENREF_1)] |
| *xrn1∆* | yVB1810 | BY4741 *xrn1∆*::*kanMX* | This work |
| *XRN1-GFP* |  | BY4741 *XRN1-GFP(HIS3)* | [[2](#_ENREF_2)] |

**Supporting references**

1. Winzeler EA, Shoemaker DD, Astromoff A, Liang H, Anderson K, Andre B, et al. Functional characterization of the S. cerevisiae genome by gene deletion and parallel analysis. Science. 1999;285(5429):901-6. Epub 1999/08/07. doi: 7737 [pii]. PubMed PMID: 10436161.

2. Huh WK, Falvo JV, Gerke LC, Carroll AS, Howson RW, Weissman JS, et al. Global analysis of protein localization in budding yeast. Nature. 2003;425(6959):686-91. Epub 2003/10/17. doi: 10.1038/nature02026 [pii]. PubMed PMID: 14562095.

3. Beliakova-Bethell N, Beckham C, Giddings TH, Jr., Winey M, Parker R, Sandmeyer S. Virus-like particles of the Ty3 retrotransposon assemble in association with P-body components. RNA. 2006;12(1):94-101. Epub 2005/12/24. doi: 12/1/94 [pii]10.1261/rna.2264806. PubMed PMID: 16373495; PubMed Central PMCID: PMCPMC1370889.
