# Supplementary material for: Ty3 Retrotransposon Hijacks Mating Yeast RNA Processing Bodies to Infect New Genomes
Source: PLoS Genet. 2015 Sep 30;11(9):e1005528. doi: 10.1371/journal.pgen.1005528 (PMC4589538; doi:10.1371/journal.pgen.1005528)
Supplement: S2 Table — Table listing primers used in this study. (DOCX) [file pgen.1005528.s008.docx]

| **S2 Table.** Primers used in this study. | | |
| --- | --- | --- |
| **Strain / plasmid /**  **fragment** | **Name** | **Sequence^1^** |
| yKC1846 | *dcp2Δ* | F5-ATAATATTGCTTTGAATCTGAAAAAAATAAAAGTACCT  TCGCATTAGACAACATGGAGGCCCAGAATACCC-3  R5- CGGCTGCCTTCATTTACAGTGTGTCTATAAAACGTATA  ACACTTATTCTTCAGTATAGCGACCAGCATTCAC-3  CF5- CCTCCTTAAGCTCACTTAGT-3  CR5- CATCGCTTCGGCTGCCTTCA-3 |
| yVB1697 | *DED1-GFP* | F5-CTGGGGTAACAGCGGTGGTTCAAACAACTCTTCTTGGT  GGGGTCGACGGATCCCCGGG-3  R5-GAGCAGAAAACGAAGAATCCTCACCCTAGTTTGTCTGA  AAATCGATGAATTCGAGCTCG-3  CF5-GAAGCTAACCAAGAAGTCCCATCATT-3  CF5-CCATCTAATTCAACAAGAATTGGGACAAC-3 |
| yVB1829 | *DHHI-GFP*  *far1Δ* | for GFP from pFA6a-GFP(S65T)-kanMX6*:*  F5-AGCAGGAACATTTCATGGCGATGCCACCTGGTCAGTCA  CAACCCCAGTATCGGATCCCCGGGTTAATTAA-3  R5-AAGCTAAACAGATCTATATTACCCTGTTATCCCTAGCG  GATCTGCCGGTA-3  for loxP-*LEU2*-loxP from pXP322:  F5-TACCGGCAGATCCGCTAGGGATAACAGGGTAATATAGATCT  GTTTAGCTTGCAGGTCGACTCTAGAGGATC-3  R5- GATCACAAAAAAAGCGTATCTCACCACAGTAGTTATTT  TTTCTTAGATATTCTGAATTCGAGCTCGGTACCCGG-3  CF5- GCCATCGCAGCAGGGGTATC-3  CR5- TATCACGAAATTCACACACTTCCATTG-3 |
| yVB1777 | *DHH1-GFP* Ty3(Δ) | F5-TTTCATGGCGATGCCACCTGGTCAGTCACAACCCCAGT  ATCGGATCCCCGGGTTAATTAA-3  R5-GCGTATCTCACCACAGTAGTTATTTTTTCTTAGATATTC  TATCGATGAATTCGAGCTCG-3  CF5-GCCATCGCAGCAGGGGTATC-3  CR5-CCATCTAATTCAACAAGAATTGGGACAAC-3 |
| yVB1830 | *DHH1-GFP* Ty3(Δ) | for GFP from pFA6a-GFP(S65T)-kanMX6*:*  F5-AGCAGGAACATTTCATGGCGATGCCACCTGGTCAGTCA  CAACCCCAGTATCGGATCCCCGGGTTAATTAA-3  R5-AAGCTAAACAGATCTATATTACCCTGTTATCCCTAGCG  GATCTGCCGGTA-3  for loxP-*LEU2*-loxP from pXP322:  F5-TACCGGCAGATCCGCTAGGGATAACAGGGTAATATAGATCT  GTTTAGCTTGCAGGTCGACTCTAGAGGATC-3  R5-GATCACAAAAAAAGCGTATCTCACCACAGTAGTTATTT  TTTCTTAGATATTCTGAATTCGAGCTCGGTACCCGG-3  CF5- GCCATCGCAGCAGGGGTATC-3  CR5- TATCACGAAATTCACACACTTCCATTG-3 |
| yVB1831 | *DHH1-GFP* *far1Δ*  Ty3(Δ) | In yVB1827:  for GFP from pFA6a-GFP(S65T)-kanMX6*:*  F5- AGCAGGAACATTTCATGGCGATGCCACCTGGTCAGTCA  CAACCCCAGTATCGGATCCCCGGGTTAATTAA-3  R5- AAGCTAAACAGATCTATATTACCCTGTTATCCCTAGCG  GATCTGCCGGTA-3  for loxP-*LEU2*-loxP from pXP322:  F5-TACCGGCAGATCCGCTAGGGATAACAGGGTAATATAGAT  CTGTTTAGCTTGCAGGTCGACTCTAGAGGATC-3  R5-GATCACAAAAAAAGCGTATCTCACCACAGTAGTTATTT  TTTCTTAGATATTCTGAATTCGAGCTCGGTACCCGG-3  CF5- GCCATCGCAGCAGGGGTATC-3  CR5- TATCACGAAATTCACACACTTCCATTG-3 |
| yVB1808 | *eap1∆* | F5-CACTTTGGTTTAGCAGCTAGTCATGTAATGGCACTA-3  R5-TCCTTCGACTAGTAAATATCAAAGGCGAAAACA-3  CF5-GACTTATCATTCACTTTGGTTTAGC-3  CR5-TTCCGAGCGTGACGTTACTAA-3 |
| yVB1827 | *far1Δ*  Ty3(Δ) | F5-ACTATCCTTTTTGTTCACTCTGTCTTG-3  R5-ATTAGCCACATCGACGTTTCTTTTACT-3  CF5-TAAAAAGGAAATAGGCAATAAAATGACA-3  CR5-AGATAAACAACGCCAAACGAAAAA-3 |
| yVB1926 | *HIS3Δ*  *lys2Δ0* | for *HIS3*:  F5-CAGGCAAGATAAACGAAGGCAAAGATG-3  R5-GTGGCTTCTCTTATGGCAACC-3  CF5-CCACCTAGCGGATGACTCTTT-3  CR5-GCTGCAGCTTTAAATAATCGGTGTC-3  for *lys2*Δ*0:*  F5- GTGAAAAACTGCTAATTATAGAGAGATATCACAGAGTTACT  CACTAATG ACATGGAGGCCCAGAATACCC-3  R5- CATATTTAATTATTGTACATGGACATATCATACGTAATGCTC  AACCTTACAGTATAGCGACCAGCATTCAC-3  CF5- ATTTTCTTCTTGCTGACCGCTTCT-3  CR5- GCGGTGTTGCTTTGAATCTTTG-3 |
| yVB1811 | *lsm1∆* | F5-TCGTGAATATACAACGGTGTTCAGCACCTGTA-3  R5-AAAAAGAATGCTGCGCAAATACGTTACTTCATAAA-3  CF5-TACCCGCGTTCGTTGAATAATGGAAAATATG-3  CR5-ATTCGTTGCTAACGGCATGGCATAAACA-3 |
| yVB1913 | *met15Δ0* | F5-TACAGGGTCGTCAGATACATAGATACAATTCTATTACCCCC  ATCCATACA ACATGGAGGCCCAGAATACCC-3  R5-TTGTGAGAGAAAGTAGGTTTATACATAATTTTACAACTCATT  ACGCACACCAGTATAGCGACCAGCATTCAC-3  CF5-TAATACAGGGTCGTCAGATACATAGATACAA-3  CR5-AGGTTCAAAGTACGAGTCACGACATGT-3 |
| yVB1819 | *pat1∆* | F5-CCCAGCCGCATCAAATTCCCCTCTT-3  R5-TGCTACCAGTTCAACCTTATTCTCAA-3  CF5-TTCTCTAACCGGTTGATCCTACTCG-3  CR5- CTGCAGCGAGGAGCCGTAAT -3 |
| yVB1813 | *pub1∆* | F5-GGCCGTCTTTCCTTGTCCTTCATTTTCCTCTC-3  R5-TCTCTTTATTCTTTCTTTTTGTTTCATT-3  CF5-ACTCGTTCTCTTTTCATCATTTTGTA-3  CR5-TGACGAAGGAAGGAAATAAGACACAGAAAAACTAA-3 |
| yVB1696 | *PUB1-GFP* | F5-TCTGAGCAACAACAGCAACAGCAGCAACAGCAGCAAC  AACAAGGTCGACGGATCCCCGGG-3  R5-ATTCTTTCTTTTTGTTTCATTCCACTTTTCTTCATAATAT  ATCGATGAATTCGAGCTCG-3  CF5-GCCAACCCCAACAATCCCAGACCAT-3  CR5-CCATCTAATTCAACAAGAATTGGGACAAC-3 |
| yVB1774 | *STM1-GFP* | F5-GAACCGTAACATTGACGTTTCTAACTTGCCATCTTTGGC  TCGGATCCCCGGGTTAATTAA-3  R5-TTATTGGATTCTTTCAGTTGGAATTATTCATATATAAGG  CATCGATGAATTCGAGCTCG-3  CF5-TCGGTGACAGAAACAACAACAGCAGA-3  CR 5-CCATCTAATTCAACAAGAATTGGGACAAC-3 |
| yVB1812 | *tif4631∆* | F5-GTGTGCAACGGATGGATGGTAGATGG-3  R5-TCGAGAAAATATAACAATGGTAAATTAGGGACACTT-3  CF 5-ACCGTATTTTTGTGCGTCCTTTTT-3  CR 5-CAGAAAGGCATTGACATATAGAACA-3 |
| yVB1700 | *TIF4631-GFP* | F5-TATGTTCAGTGCATTAATGGGAGAAAGTGATGACGAA  GAGGGTCGACGGATCCCCGGG-3  R5-ATCCAAGTGACATTTTCGATACTTAACATGATCTATTC  ATGATCGATGAATTCGAGCTCG-3  CF5-GGTTTGGTTCGTTTCATCGGTTTCCTA-3  CR5-CCATCTAATTCAACAAGAATTGGGACAAC-3 |
| yVB1810 | *xrn1∆* | F5-CGTACTTATAATCGGGTTCACAAT-3  R5-CAAAATGAGATCAATGAGAAGAAAGTGC-3  CF-5TCTCTGCCTTTTATTTCCGTTCC-3  CR-5TTACACATGACAAAGAAAGAACTG-3 |
| *his* | Ty3-*his* for riboprobe | F5-CCCGAATTCTAATACGACTCACTATAGGACGGCTGGTCGC  TAATCGTTGAGTGC-3  R5-CCCGGATCCGGATGAAGAAAAAAAATGTTTCTTGGCTGCA  GCTTTAAATAATCGGTGTCACTA-3 |
| *hisAI* | *hisAI* intron for riboprobe | F5-CCCGAATTCTAATACGACTCACTATAGGATGTTATAAATAAT  ACCATTTGTTAGTAAAAATTC-3  R5-CCCGGATCCGGATGAAGAAAAAAAATGTTTGTATGTTAATA  TGGACTAAAGGAGG-3 |
| *SNR17A* | *SNR17A* for riboprobe | F5-CCCGAATTCTAATACGACTCACTATAGGAACTTGTCAGACT  GCCATTTGTACC-3  R5-CCCGGATCCGGATGAAGAAAAAAAATGTTTGTCGACGTACT  TCAGTATGTAATATACCC-3 |
|  | Ty3 Gag3  for FISH | 5-ATTCTACTGGGAGTTTTGGATAATTTCCTCCTCCTGGGATTTG  AT -3 |
|  | Ty3 RT  for FISH | 5-CAGCTTTCTGTGTCGATAGGTCGGGATGTTTCGGGGGT  TATAGTATATA-3 |
|  | Ty3 IN  for FISH | 5-CTCAGGGTTGTCTTGTGTTTCGTAGAGTTGTCTAAAGTT  GGCTAATAT-3 |
| *SNR17A* | *SNR17A*  DNA probe | F5-GTCGACGTACTTCAGTATGTAATATACCCCAAAC-3  R5-CAGACTGCCATTTGTACCCACCCATAGAGCCCTA-3 |
| *GAG3* | Gag3 qPCR  fragment | F5-CGAACTTGATGCTGATGGAGAC-3  R5-GATCTTCTTGTCCTTACGGTATGG-3 |
| *ACT1* | actin qPCR  fragment | F5-ATTCTGAGGTTGCTGCTTTGG-3  R5-TGTCTTGGTCTACCGACGATAG-3 |
| ^1^F, forward primer; R, reverse primer; CF, forward primer for verification; CR, reverse primer for verification | | |
