# Supplementary material for: Ty3 Retrotransposon Hijacks Mating Yeast RNA Processing Bodies to Infect New Genomes
Source: PLoS Genet. 2015 Sep 30;11(9):e1005528. doi: 10.1371/journal.pgen.1005528 (PMC4589538; doi:10.1371/journal.pgen.1005528)
Supplement: S3 Table — Table listing plasmids used in this study. (DOCX) [file pgen.1005528.s009.docx]

| **S3 Table.** Plasmids used in this study. | | | |
| --- | --- | --- | --- |
| **Plasmid** | **Contents** | **Genetic markers** | **Source / Reference** |
| pYES 2.0 |  | *URA3*, 2µ, Amp^R^ | Invitrogen |
| pFA6a-GFP(S65T)-kanMX6 | GFP(S65T)-kanMX6 | Amp^R^ | [[1](#_ENREF_1)] |
| pHO-c12 | HO endonuclease | *URA3*, 2µ, Amp^R^ | [[2](#_ENREF_2)] |
| pBS34 | mCherry replaces GFP in pFA6a-GFP(S65T)-kanMX6 | Amp^R^ | Yeast Resource Center, [University of Washington](http://www.washington.edu/), Seattle, WA. |
| pDLC201 | Ty3; (*GAL1-10* UAS) | *URA3*, 2µ, Amp^R^ | [[3](#_ENREF_3)] |
| pTM218 | Ty3 with *his3AI*; (*GAL1-10* UAS) | *URA3*, 2µ, Amp^R^ | (Gift from T. Menees)[[4](#_ENREF_4)] |
| pRS316 |  | *URA3,CEN/ARS,* Amp^R^ | ATCC 77145 |
| pXP322 | *loxP-LEU2-loxP* cassette | *URA3,CEN/ARS,* Amp^R^ | [[5](#_ENREF_5)] |
| pEUTy3-1 (pPK712) | Ty3 | *URA3*, 2µ, Amp^R^ | [[6](#_ENREF_6)] |
| pNB2176 | Ty3-IN-GFP | *URA3*, *CEN/ARS*, Amp^R^ | [[7](#_ENREF_7)] |
| pNB2183 | UTR-*GAG3*-*POL3*-UTR; (*GAL1-10* UAS) | URA3, 2µ, Amp^R^ | [[8](#_ENREF_8)] |
| pNB2241 | *GAG3*-*POL3;* (*GAL1-10* UAS) | URA3, 2µ, Amp^R^ | [[8](#_ENREF_8)] |
| pNB2242 | *GAG3;* (*GAL1-10* UAS) | URA3, 2µ, Amp^R^ | [[8](#_ENREF_8)] |
| pLZL2423 | Ty3 *GAG3* (IPTG inducible) | Amp^R^  (pET3a) | [[9](#_ENREF_9)] |
| pLZL2519 | Ty3 K15A; (*GAL1-10* UAS) | *URA3*, 2µ, Amp^R^ | This work |
| pNB2622 | Ty3 (NCΔ) |  | [[9](#_ENREF_9)] |
| pVB3011 | L-BC *GAG* (P_GAL1_) | *URA3*, 2µ, Amp^R^ (pYES2.0) | This work |
| pNB3027 | UTR-*GAG3*-UTR; (*GAL1-10* UAS) | *URA3*, 2µ, Amp^R^ | [[8](#_ENREF_8)] |
| pBF3038 | *CreA;* (*GAL1-10* UAS) | *URA3*, 2µ, Amp^R^ | [[10](#_ENREF_10)] |
| pDM3193 | Ty3 with *his3AI* | *URA3*, 2µ, Amp^R^ | This work |
| pDM3194 | Ty3 (IN D225E, E261D mutations) with *his3AI* | *URA3*, 2µ, Amp^R^ | This work |
| pTD3547 | *Sa*l1, *Xho*1 and *Kpn*1 deleted from MCR of pRS316 | *URA3,CEN/ARS,* Amp^R^ | This work |
| pTD3548 | Ty3 IN-GFP | *URA3*, *CEN/ARS*, Amp^R^ (pTD3547) | This work |
| pTD3655 | Ty3 IN-mCherry | *URA3*, *CEN/ARS*, Amp^R^ (pTD3547) | This work |
| pTD3685 | Ty3 | *URA3*, *CEN/ARS*, Amp^R^ (pTD3547) | This work |
| pPS3705 | Ty3 (NCΔ) | *URA3, CEN/ARS*, Amp^R^ (pTD3547) | This work |
| pVB3734 | Ty3 GAG3-mCherry | *URA3, URA3*, *CEN/ARS*, Amp^R^ (pTD3547) | This work |

**References**

1. Wach A, Brachat A, Alberti-Segui C, Rebischung C, Philippsen P. Heterologous HIS3 marker and GFP reporter modules for PCR-targeting in Saccharomyces cerevisiae. Yeast. 1997;13(11):1065-75. Epub 1997/09/18. doi: 10.1002/(SICI)1097-0061(19970915)13:11<1065::AID-YEA159>3.0.CO;2-K [pii]. PMID: 9290211.

2. Herskowitz I, Jensen RE. Putting the HO gene to work: practical uses for mating-type switching. Methods Enzymol. 1991;194:132-46. Epub 1991/01/01. PMID: 2005783.

3. Hansen LJ, Chalker DL, Sandmeyer SB. Ty3, a yeast retrotransposon associated with tRNA genes, has homology to animal retroviruses. Mol Cell Biol. 1988;8(12):5245-56. Epub 1988/12/01. PMID: 2854194.

4. Sadeghi N, Rutz ML, Menees TM. Thermal blockage of viruslike particle formation for the yeast retrotransposon Ty3 reveals differences in the cellular stress response. Arch Virol. 2001;146(10):1919-34. Epub 2001/11/28. PMID: 11722014.

5. Luo GZ, MacQueen A, Zheng G, Duan H, Dore LC, Lu Z, et al. Unique features of the m6A methylome in Arabidopsis thaliana. Nature communications. 2014;5:5630. Epub 2014/11/29. doi: 10.1038/ncomms6630. PMID: 25430002.

6. Kinsey PT, Sandmeyer SB. Ty3 transposes in mating populations of yeast: a novel transposition assay for Ty3. Genetics. 1995;139(1):81-94. Epub 1995/01/01. PMID: 7705653.

7. Beliakova-Bethell N, Beckham C, Giddings TH, Jr., Winey M, Parker R, Sandmeyer S. Virus-like particles of the Ty3 retrotransposon assemble in association with P-body components. RNA. 2006;12(1):94-101. Epub 2005/12/24. doi: 12/1/94 [pii]10.1261/rna.2264806. PMID: 16373495.

8. Clemens K, Bilanchone V, Beliakova-Bethell N, Larsen LS, Nguyen K, Sandmeyer S. Sequence requirements for localization and packaging of Ty3 retroelement RNA. Virus Res. 2013;171:319-31. Epub 2012/10/18. doi: 10.1016/j.virusres.2012.10.008. PMID: 23073180.

9. Larsen LS, Beliakova-Bethell N, Bilanchone V, Zhang M, Lamsa A, Dasilva R, et al. Ty3 nucleocapsid controls localization of particle assembly. J Virol. 2008;82(5):2501-14. Epub 2007/12/21. doi: JVI.01814-07 [pii]10.1128/JVI.01814-07. PMID: 18094177.

10. Fang F, Salmon K, Shen MW, Aeling KA, Ito E, Irwin B, et al. A vector set for systematic metabolic engineering in Saccharomyces cerevisiae. Yeast. 2010;(2):123-36. Epub 2010/10/12. doi: 10.1002/yea.1824. PMID: 20936606.
