# Supplementary material for: Ty3 Retrotransposon Hijacks Mating Yeast RNA Processing Bodies to Infect New Genomes
Source: PLoS Genet. 2015 Sep 30;11(9):e1005528. doi: 10.1371/journal.pgen.1005528 (PMC4589538; doi:10.1371/journal.pgen.1005528)
Supplement: S7 Table — Table showing comparison of Ty3 transposition frequency and retrosome formation in mutant strains. (DOCX) [file pgen.1005528.s013.docx]

| **S7 Table.** Comparison of Ty3 transposition frequency and retrosome formation in mutant strains | | | | | | |  |
| --- | --- | --- | --- | --- | --- | --- | --- |
| Strain | Tnspnfold decr | Cells with foci  (% ± SD) | Cells with foci fold decr | | *p*^1^ | | |
| WT | 1.0 | 94 ± 5 | 1.0 | |  | |  |
| *dcp2 Δ* | 2.5* | 52 ± 6 | 1.8 | | ˂0.0001 | |  |
| *dhh1Δ* | >541* | 8 ± 3 | 11.8 | | ˂0.0001 | |  |
| *eap1Δ* | 20.0* | 48 ± 6 | 2.0 | | ˂0.0001 | |  |
| *edc3Δ* | 1.0 | 76 ± 3 | 1.2 | | 0.0025 | |  |
| *lsm1Δ* | 3.6* | 15 ± 8 | 6.3 | | ˂0.0001 | |  |
| *pat1Δ* | 1.4 | 93 ± 4 | 1.0 | | 0.8570 | |  |
| *pub1Δ* | 3.3* | 90 ± 6 | 1.0 | | 0.5066 | |  |
| *stm1Δ (YLR150W)* | 4.0* | 91 ± 2 | 1.0 | | 0.7780 | |  |
| *tif4631Δ (eIF4G1)* | 3.4* | 80 ± 11 | 1.2 | | 0.0558 | |  |
| *xrn1Δ* | 3.8* | 38 ± 5 | 2.5 | | ˂0.0001 | |  |
| *Significant decrease in transposition | | | |  | |  |  |
| ^1^ t test assuming equal variances (two-tailed) | | | |  | |  |  |
