# Supplementary material for: Ty3 Retrotransposon Hijacks Mating Yeast RNA Processing Bodies to Infect New Genomes
Source: PLoS Genet. 2015 Sep 30;11(9):e1005528. doi: 10.1371/journal.pgen.1005528 (PMC4589538; doi:10.1371/journal.pgen.1005528)
Supplement: S1 Text — (DOCX) [file pgen.1005528.s014.docx]

**S1 Text Supplemental Materials and Methods**

**Strains and growth conditions**

Yeast and bacterial culture methods were as previously described [[1](#_ENREF_1), [2](#_ENREF_2)] except where noted. Bacterial strains HB101 [F^−^*hsd-20* (*rB*^−^*mB*^−^) *recA13 leuB6 ara14 proA2 lacY1 galK2 rpsL20* (Sm^r^) xyl-5 myl-1 supE44λ] or DH5α [F^−^Φ80*lacZ*Δ *M15*Δ (*lacZYA*-*argF*) *U169 deoR recA1 endA1 hsdR17*(r_K_^−^m_K_^+^) *phoA supE44 thi-1 gyrA96 relA1* λ^−^] were used for plasmid preparations. All *S.cerevisiae* strains were derivatives of BY4741 (*MAT***a** *his3Δ1 leu2Δ0 met15Δ0 ura3Δ0*) (Open Biosystems, Inc.). (S1 Table). Yeast cultures were grown at 24^o^C for Ty3 expression. Yeast cells were grown in either complete medium (1% yeast extract, 2% peptone, 2% dextrose) or in synthetic dextrose medium [0.67% yeast nitrogen base, 2% dextrose], containing complete amino acids, inositol and adenine sulfate lacking selection nutrients for selection and growth of cells transformed with plasmids containing particular prototrophic markers. For galactose-regulated expression, cells were grown in synthetic raffinose medium [0.67% yeast nitrogen base, 1% raffinose, 2% v/v glycerol, 2% v/v sodium lactate] containing complete amino acids, inositol and adenine sulfate lacking selection nutrients, galactose was added to a final concentration of 2% and cells were grown for 2 h. For pheromone induction, yeast cultures were grown at 24^o^C to OD_600_ of 0.2 and α-factor was added to 6 µM final concentration for the indicated time. To visualize individual proteins by fluorescence microscopy, strains were derived by fusion of the genomic ORF in-frame to the sequence encoding *GFP* (*S65T*)[[3](#_ENREF_3)] or mCherry in a similar manner. For some live cell imaging experiments, cells were transformed with plasmids expressing Ty3 proteins under the native promoter fused in-frame to either GFP or mCherry (S3 Table).

**Yeast strain construction**

*S. cerevisiae* strains with deletion of specific ORFs were made by replacement of the ORF in BY4741 by *KanMX* [[4](#_ENREF_4)]. Strain yVB1913 was converted to *HIS3* by transformation with a PCR-generated fragment. Mating-type switching was done using pHO-c12 as described [[5](#_ENREF_5)]. Strains with ORF-GFP or -mCh fusions were made in BY4741 using either plasmid pFA6a-GFP(S65T)-kanMX6 or pBS34 essentially as described [[6](#_ENREF_6)]. Some strains with *DHH1-GFP* were made in a similar manner except *loxP-LEU2-loxP* from plasmid pXP322 [[7](#_ENREF_7)] was used for selection. BY4741 was cured of killer L-A by treatment with 0.8 µM anisomycin for 4 days [[8](#_ENREF_8)], and cured of L-BC by overexpression of L-BC *GAG* (P_GAL1_) for 24 h as originally described [[9](#_ENREF_9)] from plasmid pVB3011. Strain yVB1586 was verified to be cured of L-A and L-BC by northern blot analysis using probes specific for L-A and L-BC (S6 Fig.).

**Antibodies**

Polyclonal antibodies (Berkeley Antibody Company) were raised in rabbits against purified WT Ty3 VLPs [[10](#_ENREF_10)]. The antibodies were purified using Gag3 affinity column. For western blot analysis, antibody against capsid [[11](#_ENREF_11)] was used at 1-5000 dilution and antibody against yeast phosphoglycerate kinase (Pgk1, Invitrogen) was used at 1-5000 dilution for a loading control. For immunofluorescence microscopy (IF), anti-VLP antibody was used at 1-5000 dilution to detect Gag3 products. Proteins were visualized using Alexa Fluor 568-conjugated donkey anti-rabbit IgG (Life Technologies). Digoxigenin (DIG)-labeled probes were detected using anti-DIG-fluorescein Fab fragments (1-200 dilution, Roche Applied Science).

**Mass spectrometry**

Rabbit polyclonal anti-VLP antibodies (Covance Research Products Inc.) were affinity purified using recombinant Gag3 expression from pLZL2423 [[12](#_ENREF_12), [13](#_ENREF_13)], and cross-linked to AminoLink Plus Resin according to manufacturer’s instructions (Thermo Scientific Inc.). Control non-immunized rabbit IgG was similarly immobilized. Independent cultures of yVB1586 transformed with pDLC201 expressing WT Ty3 or pLZL2519 expressing K15A mutant Ty3 under *GAL1-10* UAS were induced by growth in galactose-containing medium for 2 h. K15A is a Ty3 mutant with elevated transposition frequency [Ty3 (WT) 1.40 ± 0.03 x10^-4^ versus Ty3 (Gag3-K15A) 20.3 ± 5.3 x 10^-4^]. Cells were frozen in liquid N_2,_ pulverized in a ball mill (Retsch, Inc., Newtown, PA) [[14](#_ENREF_14)] and solubilized in buffer [20 mM Hepes pH 7.2, 100 mM KCl, 10 mM EDTA 0.1% NP40) containing proteinase inhibitors [1 mM PMSF, 1 µg/ml pepstatin A, 1µg/ml leupeptin]. Clarified extracts were chromatographed separately over control IgG or anti-VLP matrices [[12](#_ENREF_12), [13](#_ENREF_13)]. Aliquots of eluted proteins were analyzed by SDS-PAGE and immunoblotting. Remaining sample was solubilized in 8 M urea, reduced, alkylated and digested with LysC and then trypsin as described [[15](#_ENREF_15)]. Peptides were analyzed by 1DLC-MS/MS using LTQ-Orbitrap XL MS (ThermoElectron) as described [[15](#_ENREF_15)]. Monoisotopic masses of parent ions and corresponding fragment ions were extracted using in house software based on Raw_Extract script from Xcalibur v2.4 and extracted data were searched and analyzed using the Batch-Tag and Search Compare within the developmental version (v. 5.2.2) of Protein Prospector as described [[15](#_ENREF_15)]. Proteins were identified by at least two peptides with a false-positive rate of 0.1%. Based on the MS/MS results from both wild type and K15A mutant samples, we identified 154 non-overlapping proteins. To discriminate background proteins from potential Ty3 interacting protein targets, we used a semi-quantitative analysis based on spectral count. Only identified proteins with a total peptide count of at least 4 times greater than the total peptide count from control (IgG) were considered as Ty3 interacting proteins. Based on the MS/MS results from both WT and K15A mutant samples, we identified 154 non-overlapping proteins. To discriminate background proteins from potential Ty3 interacting targets, we used a semi-quantitative analysis based on spectral count. Only identified proteins with a total peptide count of at least 4 times greater than the total peptide count from control (IgG) were considered as Ty3 interacting proteins. Of the154 identified proteins, 107 are not essential (S4 Table). Ty3 transposition was measured in these strains. In 45 strains (42%), the frequency was significantly different from WT [8 were elevated and 32 were decreased (equal to or greater than twofold)].

**FISH**

The procedure was adapted from previously published work [[16](#_ENREF_16), [17](#_ENREF_17)]. Cells were grown at 24ºC in YPD to OD_600_ of 0.2, and either incubated with pheromone for 4 h or left untreated. Cells transformed with a plasmid were grown in the appropriate synthetic medium for selection and induced in the same manner. Cells were fixed by the addition of 0.1 volume of 37% formaldehyde. After 15 min, cells were harvested by centrifugation and resuspended in 4 ml 4% paraformaldehyde, 0.1M potassium phosphate pH 6.5, 5 mM MgCl_2_. After 3 h, cells were washed twice with solution B (1.2 M sorbitol, 0.1 M potassium phosphate pH 6.5). For spheroplasting, cells were resuspended in freshly prepared solution B containing 0.005% β-mercaptoethanol, 30 units RNasin/ml (Promega), 0.3 mg/ml Zymolyase 100T (ICN Biochemicals) and incubated for 15-20 min at 37ºC. Spheroplasts were washed 3 times with solution B, resuspended in solution B and applied to poly-L-lysine-coated coverslips (Electron Microscopy Sciences). Non-adhered cells were removed by aspiration. Cells were treated successively with 70, 90 and 100% ethanol for 5 min each and stored in 70% ethanol at -20ºC until use. Cells were treated with 0.1% Triton X-100 for 5 min, washed with solution B, treated with 0.1M triethanolamine hydrochloride (TEA) for 2 min, treated with 0.1 M TEA, 2.5% acetic anhydride for 10 min, and washed with 4 X SSC. Cells were prehybridized with 40% formamide, 4 x SSC, 1 x Denhardt’s solution, 0.25 µg/ml tRNA, 0.25 µg/ml sheared salmon sperm DNA, 5 mM EDTA, RNasin (400 units/ml)for 1 h at 37ºC in a humid chamber. Ty3 mRNA was detected with an equal parts mixture of antisense oligonucleotides (complementary to Ty3 Gag3;

5’ ATTCTACTGGGAGTTTTGGATAATTTCCTCCTCCTGGGATTTGAT 3’),

(complementary to Ty3 RT; 5’CAGCTTTCTGTGTCGATAGGTCGGGATGTTTCGGGGGTTATAGTATATA 3’) and (complementary to Ty3 IN; 5’ CTCAGGGTTGTCTTGTGTTTCGTAGAGTTGTCTAAAGTTGGCTAATAT 3’)

synthesized and HPLC-purified by Integrated DNA Technologies, Inc . Oligonucleotides were 3’ end-labeled with digoxigenin (DIG)-dUTP using the DIG Oligonucleotide Labeling Kit, 2nd generation as instructed by the manufacturer (Roche Applied Science). Labeled oligonucleotides were purified using Micro Bio-Spin P-30 Tris RNase-Free column (Bio-Rad). Hybridization was carried out in the same solution containing an equal parts mixture of DIG-labeled oligonucleotides (approximately 5pM each oligonucleotide/coverslip) overnight at 37ºC. Cells were washed twice with 2 X SSC, 40% formamide at 37ºC for 10 min each, washed once with 1 X SSC at 37ºC for 5 min, and twice with 1 X SSC at room temperature for 5 min each. IF detection of Ty3 RNA using anti-DIG and Ty3 protein using anti-VLP was carried out sequentially as follows. For IF detection of DIG, cells were washed once with 1 X PBS for 5 min, once with 10 mM potassium phosphate buffer pH 6.5, 0.02% SDS for 2 min, once with 1 X PBS for 5 min and blocked in 1% Roche block (Roche Applied Science) in 1 X PBS for 1 hr at room temperature. Cells were incubated with 1-200 dilution of fluoresceinated anti-DIG Fab fragment (Roche Applied Science) in 0.5% Roche block, 1 X PBS, 10 mM RVC (ribonucleoside vanadyl complex, New England Biolabs) for 1.5 h at room temperature, washed once with 8% formamide, 2 X SSC for 5 min, three times with 1 X PBS, 0.2% Tween 20 for 5 min each and once with 1 X PBS for 5 min at room temperature. For detection of Ty3 protein, cells were blocked in 5% normal horse serum (Gibco) in 1 X PBS, 10 mM RVC for 1 h, incubated with 1-2,500 dilution of anti-VLP (pre-cleared by incubating with acetone precipitated yeast protein extract from Ty3 null strain) in 1% normal horse serum, 10 mM RVC for 1.5 h, and washed five times with 1 X PBS, 0.2% Tween 20, 1% normal horse serum, 10 mM RVC for 5 min each. Cells were incubated with 1-1000 dilution of Alexa-fluor 568-conjugated donkey anti-rabbit IgG (Life Technologies) in 1% normal horse serum, 1 X PBS, 10 mM RVC for 1 h at room temperature, washed five times with 1 X PBS, 0.2% Tween 20 for 5 min each, washed once with 1 X PBS for 5 min, and once with 1 X PBS. Cells were stained with 3 µg/ml DAPI (4’, 6-diamidino-2-phenylindole), and mounted using VECTASHIELD medium (Vector Laboratories).

**Microscopy**

Cells were grown at 24^o^C in the designated medium to OD_600_ of 0.2 and either induced with pheromone for 4 h or remained uninduced as control. Fluorescent images of live cells were visualized using either a Zeiss Axioplan2 fluorescence microscope or an inverted confocal scanning microscope [Zeiss LSM510 META, Plan-Apochromat 63x/1.4NA oil objective; Department of Microbioloby and Molecular Genetics, UCI]. For FISH experiments, images were recorded using an inverted confocal laser scanning microscope [Zeiss LSM780, Plan-Apochromat 63x /1.4NA oil objective; Optical Biology Core Facility UCI]. Images were processed for publication with Adobe Photoshop CS3 (Adobe Systems Inc.). Images from at least three replicate samples consisting of at least 100 cells each were analyzed, and single plane images are shown.

**qPCR**

BY4741 cells were grown at 24^o^C in YPD medium to OD_600_ =0.2. Uninduced cells (5 OD_600_ cell pellet) were harvested. The rest of the culture was induced with α-factor and cells were harvested at 2, 4, 6 and 8 h. Cells were lysed with glass beads in a FastPrep (MP Biomedicals), RNA was extracted using RNeasy Mini Kit (Qiagen), and treated with RNase-free DNase (Qiagen) using manufacturers protocols. Total RNA (1.5 µg) was used as a template for cDNA synthesis using an oligo(dT) primer (final concentration of 0.025 µg/mL) in a Superscript II Reverse Transcriptase reaction according to the manufacturer’s protocol (Invitrogen). Quantitative real-time PCR was performed in triplicate on three independent cDNA samples as indicated using iQSYBRGreen Supermix (Bio-Rad Laboratories) according to the manufacturer’s protocol using a RT-qPCR Cycler (Bio-Rad Laboratories). The ratio of Ty3 GAG3 to *ACT1* signal was calculated for each construct. The primers used were: for GAG3, F5- CGAACTTGATGCTGATGGAGAC-3, R5-GATCTTCTTGTCCTTACGGTATGG-3; for ACT1, F5-ATTCTGAGGTTGCTGCTTTGG-3, R5-TGTCTTGGTCTACCGACGATAG-3.

**RNA, protein, RNA packaging and cDNA analysis**

RNA, protein RNA packaging and cDNA levels were essentially as described [[18](#_ENREF_18)]. Briefly, total RNA was prepared from cells induced with pheromone for 8 h, total RNA was isolated and quantified by northern blotting. WT Ty3 RNA signal of the ^32^P-labeled DNA probe specific to a 2.9 kb region (*Bgl*II fragment) of the Ty3 genome was normalized to the signal of the cellular RNA control snR17a. Ty3 protein level in 2 and 6 h α-factor-induced or 6 h galactose-induced cells from two or more independent experiments was determined by western blot analysis. Harvested cells were lysed under denaturing conditions to generate whole cell extracts and analyzed. The sum of Ty3 protein levels were normalized to Pgk1 signal and averaged. WT values at 2 and 6 h were set and mutant values were normalized by a fold factor. To monitor the efficiency of RNA packaging into Ty3 VLPs, an RNA nuclease-protection assay was performed essentially as described [[19](#_ENREF_19)] with the following modifications. Cells were induced for Ty3 expression with α-factor for 8 h. Cells (10-20 OD_600_) were harvested, and subjected to extraction of RNA and protein under native conditions, and the amount of Ty3 RNA resistant to TurboNuclease (Accelagen) digestion was determined. An *in vitro* transcribed, truncated Ty3 RNA was added to the extract to monitor RNA digestion. Following treatment, RNA was purified and quantified by northern blotting. The percentage of Ty3 RNA resistant to degradation was calculated. Ty3 cDNA production in uninduced and pheromone induced cells was monitored by Southern blot analysis. DNA was extracted, digested with *Nhe*I and *Bam*HI restriction enzymes, and Ty3 endogenous elements and cDNA were detected by a ^32^P-labeled DNA probe specific to a 2.9 kb region of the Ty3 genome. A fragment of *ARG4* was detected as a loading control in the same manner. For RNA, packaging and cDNA experiments, adjusted WT measurements were set to 1.0 and three or more measurements from biological replicates for each strain were normalized to WT. Statistical significance was determined using the program R. A planned contrast, one way ANOVA was performed, with contrast between WT and individual mutant samples compared.

**Relative splicing efficiency of Ty3-*his3AI* RNA**

RNA was isolated from WT (BY4741), *lsm1Δ,* *pub1Δ,*  and *xrn1Δ* cells that were transformed with plasmid expressing Ty3-*his3AI* (pDM3193) and treated with α-factor for 2 h. RNA was analyzed by northern blot using ^32^P-RNA probes to detect *his3* (anti-sense), *AI* intron-specific (sense) and control *SNR17A* (sense) transcripts. Probes were generated *in vitro* by run-off transcription with bacteriophage T7 RNA polymerase using DNA fragments made by PCR. The T7 RNA polymerase reactions contained PCR-generated DNA fragment (1-2 pmol), 1 X T7 transcription buffer (80 mM HEPES pH7.5, 2 mM spermidine, 40 mM DTT, 25 mM MgCl_2_ ), 2 mM each NTP and including ^32^P-CTP, T7 RNA polymerase (25 units/μl final concentration). The reaction was incubated for 2 h at 37^o^C. The relative splicing efficiency in the mutant strains was normalized to WT from a minimum of 2 independent experiments (S2 Fig.).

**Velocity gradient analysis**

BY4741 cells were grown to OD_600_=0.2, induced with α-factor for 1.5 h and a portion of the culture was harvested. The α-factor was washed out of the remainder, the culture was grown for an additional 4 h for a total time of 5.5 h and cells were harvested. Cells were lysed in STEK buffer [10% w/w sucrose, 10 mM Tris, 100 mM KCl, 10 mM EDTA pH7.5] containing protease inhibitors (final concentration 1mM PMSF, 1 µg/ml pepstatin, 2 µg/ml aproteinin, 1 µg/ml leupeptin) and RNase inhibitor (RNasin, Promega). The extract was clarified by centrifugation at 4^o^C at 500 x g for 3 min, layered on top of a linear 20-60% sucrose gradient prepared in the same buffer and centrifuged at 5^o^C in a SW55Ti rotor (Beckman Coulter, Inc.) at 100,000 x g for 2 h. Fractions were collected, protein, RNA and DNA was extracted from portions of the same sample and analyzed as previously described.

**Polysome gradient analysis**

Cells were grown at 24^o^C in YPD to OD_600_ of 0.2, and induced with α-factor for 2 h. Prior to harvesting, cycloheximide was added to a final concentration of 0.1 mg/ml and incubated on ice for 5 min. Frozen cell pellets were stored at -80^o^C. Cell pellets were lysed in ice-cold extraction buffer (20mM Tris pH7.4, 100mM NaCl, 30mM MgCl_2_, 0.2 mg/ml heparin, 0.1 mg/ml cycloheximide) in a FastPrep (MP Biomedicals). Extract was clarified by centrifugation at 6,010 x g for 5 min and then at 12,420 x g for 5 min. Extract (10-12 OD_260_) were loaded onto 4-47% linear sucrose gradient prepared in gradient buffer plus Mg (50 mM Tris pH 7.6, 100 mM NH_4_Cl, 24 mM MgCl_2_, 1 mM DTT) or gradient buffer plus EDTA (50 mM Tris pH7.6, 100 mM NH_4_Cl, 20 mM EDTA, 1 mM DTT) and centrifuged at 35,000 rpm in a SW41 Ti swinging bucket rotor (Beckman Coulter, Inc.) at 4^o^C for 2.5 h. Gradient fractions (Teledyne ISCO) were analyzed for Ty3 RNA and protein as indicated.

**Supplemental References**

1. Ausubel FM, Brent R, Kingston RE, Moore DD, Seidman JG, Smith JA, et al. Current Protocols in Molecular Biology: John Wiley and Sons, Inc.; 2007.

2. Amberg DC, Burke DJ, Strathern JN. Methods in Yeast Genetics. Cold Spring Harbor: Cold Spring Harbor Laboratory Press; 2005.

3. Huh WK, Falvo JV, Gerke LC, Carroll AS, Howson RW, Weissman JS, et al. Global analysis of protein localization in budding yeast. Nature. 2003;425(6959):686-91. Epub 2003/10/17. doi: 10.1038/nature02026 [pii]. PMID: 14562095.

4. Wach A, Brachat A, Pohlmann R, Philippsen P. New heterologous modules for classical or PCR-based gene disruptions in Saccharomyces cerevisiae. Yeast. 1994;10(13):1793-808. Epub 1994/12/01. PMID: 7747518.

5. Herskowitz I, Jensen RE. Putting the HO gene to work: practical uses for mating-type switching. Methods Enzymol. 1991;194:132-46. Epub 1991/01/01. PMID: 2005783.

6. Wach A, Brachat A, Alberti-Segui C, Rebischung C, Philippsen P. Heterologous HIS3 marker and GFP reporter modules for PCR-targeting in Saccharomyces cerevisiae. Yeast. 1997;13(11):1065-75. Epub 1997/09/18. doi: 10.1002/(SICI)1097-0061(19970915)13:11<1065::AID-YEA159>3.0.CO;2-K. PMID: 9290211.

7. Fang F, Salmon K, Shen MW, Aeling KA, Ito E, Irwin B, et al. A vector set for systematic metabolic engineering in Saccharomyces cerevisiae. Yeast. 2010;(2):123-36. Epub 2010/10/12. doi: 10.1002/yea.1824. PMID: 20936606.

8. Dinman JD, Ruiz-Echevarria MJ, Czaplinski K, Peltz SW. Peptidyl-transferase inhibitors have antiviral properties by altering programmed -1 ribosomal frameshifting efficiencies: development of model systems. Proc Natl Acad Sci U S A. 1997;94(13):6606-11. Epub 1997/06/24. PubMed PMID: 9192612.

9. Yao W, Bruenn JA. Interference with replication of two double-stranded RNA viruses by production of N-terminal fragments of capsid polypeptides. Virology. 1995;214(1):215-21. Epub 1995/12/01. doi: S0042-6822(85)79938-2 [pii]10.1006/viro.1995.9938. PMID: 8525618.

10. Hansen LJ, Chalker DL, Orlinsky KJ, Sandmeyer SB. Ty3 GAG3 and POL3 genes encode the components of intracellular particles. J Virol. 1992;66(3):1414-24. Epub 1992/03/01. PubMed PMID: 1371165.

11. Menees TM, Sandmeyer SB. Transposition of the yeast retroviruslike element Ty3 is dependent on the cell cycle. Mol Cell Biol. 1994;14(12):8229-40. Epub 1994/12/01. PMID: 7969160.

12. Harlow E, Lane D. Using Antibodies, A Laboratory Manual. Cold Spring Harbor, N.Y.: Cold Spring Harbor Laboratory Press; 1999.

13. Gingras AC, Gstaiger M, Raught B, Aebersold R. Analysis of protein complexes using mass spectrometry. Nat Rev Mol Cell Biol. 2007;8(8):645-54. Epub 2007/06/28. doi: nrm2208 [pii]10.1038/nrm2208. PMID: 17593931.

14. Oeffinger M, Wei KE, Rogers R, DeGrasse JA, Chait BT, Aitchison JD, et al. Comprehensive analysis of diverse ribonucleoprotein complexes. Nat Methods. 2007;4(11):951-6. Epub 2007/10/09. doi: nmeth1101 [pii]10.1038/nmeth1101. PMID: 17922018.

15. Kaake RM, Milenkovic T, Przulj N, Kaiser P, Huang L. Characterization of cell cycle specific protein interaction networks of the yeast 26S proteasome complex by the QTAX strategy. J Proteome Res. 2010;9(4):2016-29. Epub 2010/02/23. doi: 10.1021/pr1000175. PMID: 20170199.

16. Sarkar S, Azad AK, Hopper AK. Nuclear tRNA aminoacylation and its role in nuclear export of endogenous tRNAs in Saccharomyces cerevisiae. Proc Natl Acad Sci U S A. 1999;96(25):14366-71. Epub 1999/12/10. PMID: 10588711.

17. Checkley MA, Nagashima K, Lockett SJ, Nyswaner KM, Garfinkel DJ. P-body components are required for Ty1 retrotransposition during assembly of retrotransposition-competent virus-like particles. Mol Cell Biol. 2010;30(2):382-98. Epub 2009/11/11. doi: MCB.00251-09 [pii]10.1128/MCB.00251-09. PMID 19901074;.

18. Clemens K, Bilanchone V, Beliakova-Bethell N, Larsen LS, Nguyen K, Sandmeyer S. Sequence requirements for localization and packaging of Ty3 retroelement RNA. Virus Res. 2013;171:319-31. Epub 2012/10/18. doi: 10.1016/j.virusres.2012.10.008. PMID: 23073180.

19. Larsen LS, Zhang M, Beliakova-Bethell N, Bilanchone V, Lamsa A, Nagashima K, et al. Ty3 capsid mutations reveal early and late functions of the amino-terminal domain. J Virol. 2007;81(13):6957-72. Epub 2007/04/20. doi: JVI.02207-06 [pii]10.1128/JVI.02207-06. PMID: 17442718.
