# Supplementary material for: Ty3 Retrotransposon Hijacks Mating Yeast RNA Processing Bodies to Infect New Genomes
Source: PLoS Genet. 2015 Sep 30;11(9):e1005528. doi: 10.1371/journal.pgen.1005528 (PMC4589538; doi:10.1371/journal.pgen.1005528)
Supplement: S2 Text — (DOCX) [file pgen.1005528.s015.docx]

**S2 Text. Plasmid construction and sequences.**

pLZL2519

Contents in bold: Ty3 K15A; (*GAL1-10* UAS)

Genetic markers: *URA3*, 2µ, Amp^R^

Construction: Plasmid pDLC201 (Hansen LJ, Chalker DL, Sandmeyer SB. Ty3, a yeast retrotransposon associated with tRNA genes, has homology to animal retroviruses. Mol Cell Biol. 1988; 8: 5245-5256) was modified by changing the sequence encoding K at position 15 to the sequence encoding A in *GAG3* (red) by standard methods using oligonucleotide 5-CCCAGGAGGAGGAAATTATCCAGCACTCCCAGTAGAATGC-3.

AAGCTTTTCAATTCAATTCATCATTTTTTTTTTATTCTTTTTTTTGATTTCGGTTTCTTTGAAATTTTTTTGATTCGGTAATCTCCGAACAGAAGGAAGAACGAAGGAAGGAGCACAGACTTAGATTGGTATATATACGCATATGTAGTGTTGAAGAAACATGAAATTGCCCAGTATTCTTAACCCAACTGCACAGAACAAAAACCTGCAGGAAACGAAGATAAATCATGTCGAAAGCTACATATAAGGAACGTGCTGCTACTCATCCTAGTCCTGTTGCTGCCAAGCTATTTAATATCATGCACGAAAAGCAAACAAACTTGTGTGCTTCATTGGATGTTCGTACCACCAAGGAATTACTGGAGTTAGTTGAAGCATTAGGTCCCAAAATTTGTTTACTAAAAACACATGTGGATATCTTGACTGATTTTTCCATGGAGGGCACAGTTAAGCCGCTAAAGGCATTATCCGCCAAGTACAATTTTTTACTCTTCGAAGACAGAAAATTTGCTGACATTGGTAATACAGTCAAATTGCAGTACTCTGCGGGTGTATACAGAATAGCAGAATGGGCAGACATTACGAATGCACACGGTGTGGTGGGCCCAGGTATTGTTAGCGGTTTGAAGCAGGCGGCAGAAGAAGTAACAAAGGAACCTAGAGGCCTTTTGATGTTAGCAGAATTGTCATGCAAGGGCTCCCTATCTACTGGAGAATATACTAAGGGTACTGTTGACATTGCGAAGAGCGACAAAGATTTTGTTATCGGCTTTATTGCTCAAAGAGACATGGGTGGAAGAGATGAAGGTTACGATTGGTTGATTATGACACCCGGTGTGGGTTTAGATGACAAGGGAGACGCATTGGGTCAACAGTATAGAACCGTGGATGATGTGGTCTCTACAGGATCTGACATTATTATTGTTGGAAGAGGACTATTTGCAAAGGGAAGGGATGCTAAGGTAGAGGGTGAACGTTACAGAAAAGCAGGCTGGGAAGCATATTTGAGAAGATGCGGCCAGCAAAACTAAAAAACTGTATTATAAGTAAATGCATGTATACTAAACTCACAAATTAGAGCTTCAATTTAATTATATCAGTTATTACCC**GATCAAAAATCATCGCTTCGCTGATTAATTACCCCAGAAATAAGGCTAAAAAACTAATCGCATTATCATCCTATGGTTGTTAATTTGATTCGTTCATTTGAAGGTTTGTGGGGCCAGGTTACTGCCAATTTTTCCTCTTCATAACCATAAAAGCTAGTATTGTAGAATCTTTATTGTTCGGAGCAGTGCGGCGCGAGGCACATCTGCGTTTCAGGAACGCGACCGGTGAAGACGAGGACGCACGGAGGAGAGTCTTCCTTCGGAGGGCTGTCACCCGCTCGGCGGCTTCTAATCCGTACTTCAATATAGCAATGAGCAGTTAAGCGTATTACTGAAAGTTCCAAAGAGAAGGTTTTTTTAGGCTAACTCGAGCTCGACTCTAGAGGATCCCCCTGAACTACCCAAAGTATAAATGCCTGAACAATTAGTTTAGATCCGAGATTCCGCGCTTCCACCACTTAGTATGATTCATATTTTATATAATATATAAGATAAGTAACATTCCGTGAATTAATCTGATAAACTGTTTTGACAACTGGTTACTTCCCTAAGACTGTTTATATTAGGATTGTCAAGACACTCCGGTATTACTCGAGCCCGTAATACAACACCTGGTAGCGTTAAAGGTTACTAATTGTTCAAACGAACCATCGAAAAGCCGAACCTAGCTACACCACACCCCAGTATGAGCTTTATGGATCAAATCCCAGGAGGAGGAAATTATCCAGCACTCCCAGTAGAATGCCTTCCTAACTTCCCGATCCAACCATCTTTGACCTTCAGAGGTAGAAATGACTCGCATAAACTGAAAAACTTTATCTCCGAAATAATGTTAAACATGTCTATGATATCTTGGCCGAATGATGCCAGTCGTATTGTGTACTGCAGAAGACATTTATTAAACCCCGCTGCTCAGTGGGCTAATGACTTTGTACAAGAACAAGGTATACTTGAAATAACATTCGACACATTCATACAAGGATTATATCAGCATTTCTATAAGCCACCAGATATCAATAAAATCTTTAATGCAATCACGCAACTTTCCGAAGCTAAACTTGGTATTGAGCGTCTCAACCAACGATTCAGAAAGATTTGGGACAGAATGCCACCAGACTTCATGACCGAAAAAGCTGCCATAATGACATATACTAGGCTATTGACAAAGGAAACCTATAATATTGTCAGAATGCACAAACCAGAGACATTAAAAGACGCCATGGAAGAGGCTTACCAGACAACTGCACTAACTGAAAGATTCTTCCCAGGATTCGAACTTGATGCTGATGGAGACACTATCATCGGTGCCACAACCCACTTACAAGAAGAATACGACTCTGACTATGATTCAGAAGATAATCTGACCCAGAATGGATACGTCCATACCGTAAGGACAAGAAGATCTTACAATAAACCAATGTCAAATCATCGAAACAGGAGAAATAACAACCCATCTAGAGAAGAATGTATAAAAAATCGGCTATGCTTCTATTGTAAGAAAGAGGGACATCGCCTGAACGAATGTAGAGCACGTAAGGCGAGTTCTAACCGATCTTGAACTCGAATCAAAAGACCAACAAACTCCTTTTATCAAAACCTTACCAATTGTACACTATATCGCCATCCCCGAGATGGACAATACCGCCGAAAAAACCATAAAAATACAAAACACGAAAGTAAAAACCCTGTTTGACAGTGGATCACCCACGTCATTTATCCGAAGAGATATTGTAGAACTTCTCAAATACGAAATCTACGAGACCCCTCCACTCCGTTTTAGAGGATTCGTAGCCACCAAATCCGCCGTTACATCCGAAGCAGTCACCATTGACCTCAAAATCAATGACCTGCATATAACTTTAGCCGCGTACATACTGGATAACATGGACTACCAATTGTTAATTGGAAATCCAATCTTACGCCGCTACCCGAAAATCCTGCACACAGTACTGAATACCAGAGAGAGCCCCGACTCCTTAAAGCCCAAGACTTATCGCTCCGAAACCGTTAATAACGTTAGAACCTACTCCGCTGGTAATCGTGGTAACCCCAGAAACATAAAACTGTCTTTTGCCCCCACCATTCTCGAAGCAACTGACCCGAAATCCGCTGGTAATCGTGGTGACTCCAGAACCAAAACCCTGTCTCTTGCAACCACTACTCCTGCAGCAATTGACCCGCTTACGACCCTTGATAACCCAGGTAGTACTCAAAGTACATTTGCGCAATTCCCGATACCTGAAGAAGCGAGCATCCTAGAAGAGGATGGAAAATACTCCAACGTTGTCTCAACCATTCAGAGTGTAGAACCTAATGCTACTGATCACAGCAATAAGGACACCTTTTGCACTTTGCCAGTTTGGTTACAACAGAAGTATAGAGAGATCATACGTAATGATCTCCCACCAAGACCTGCCGACATTAATAACATCCCCGTAAAACATGATATTGAAATTAAACCTGGCGCAAGACTACCTCGACTACAGCCATACCATGTTACAGAAAAGAACGAACAAGAAATCAACAAAATAGTTCAAAAACTGCTCGATAACAAGTTCATTGTTCCCTCAAAGTCGCCTTGCAGCTCCCCTGTAGTCCTCGTCCCGAAGAAAGACGGTACCTTCCGACTCTGCGTCGATTACCGCACCCTGAACAAAGCTACCATCTCCGACCCATTCCCATTACCCAGAATCGACAACCTATTGAGCCGTATTGGAAATGCCCAGATATTTACCACGCTAGATTTGCATAGTGGTTACCACCAGATCCCGATGGAACCCAAAGACCGCTACAAAACCGCCTTTGTCACACCATCCGGTAAGTATGAATATACCGTCATGCCATTTGGCTTAGTCAATGCACCTAGTACATTCGCAAGATACATGGCTGATACATTTAGAGACCTGAGATTCGTCAATGTTTACCTTGATGATATATTAATATTCTCCGAATCTCCAGAAGAACATTGGAAACATTTAGACACGGTACTAGAAAGATTAAAGAACGAGAACCTCATTGTTAAGAAGAAAAAATGTAAATTTGCATCTGAAGAAACTGAGTTTTTAGGCTATAGTATTGGAATCCAGAAAATAGCTCCACTACAGCACAAATGTGCAGCAATCCGAGACTTTCCGACGCCTAAAACAGTAAAACAAGCACAGAGATTTTTAGGAATGATTAATTACTACAGACGATTCATTCCAAATTGCTCCAAGATTGCACAGCCAATCCAACTGTTTATTTGTGACAAAAGTCAATGGACAGAAAAACAAGACAAGGCAATTGATAAACTAAAAGACGCCTTGTGTAACTCCCCCGTCCTAGTACCATTCAACAACAAAGCAAACTACCGACTTACAACAGACGCCTCAAAAGACGGCATTGGTGCTGTTCTAGAAGAAGTCGACAACAAGAACAAACTTGTTGGTGTCGTCGGTTACTTCTCTAAATCCTTAGAGAGTGCCCAGAAAAACTATCCTGCTGGCGAATTAGAACTACTTGGAATTATCAAAGCACTCCACCACTTCCGATATATGCTTCACGGAAAGCATTTCACGTTAAGAACAGACCACATTAGTTTGTTATCATTACAAAACAAGAACGAACCCGCACGACGCGTGCAACGCTGGTTAGATGACCTAGCCACATATGACTTCACCTTAGAATACCTAGCTGGACCCAAGAACGTTGTCGCAGATGCCATATCCCGTGCCGTATATACTATAACCCCCGAAACATCCCGACCTATCGACACAGAAAGCTGGAAATCTTACTACAAATCAGACCCATTATGTAGTGCTGTCTTAATTCATATGAAAGAATTGACACAACACAACGTCACACCTGAAGATATGTCAGCCTTCCGTAGTTACCAGAAGAAACTCGAACTATCAGAGACCTTCCGAAAGAATTATTCCCTAGAAGACGAAATGATCTATTACCAAGACCGACTAGTAGTACCAATAAAACAACAGAACGCAGTTATGAGACTATATCATGACCATACCTTATTTGGAGGACATTTTGGTGTAACAGTGACCCTTGCGAAAATCAGCCCAATTTACTATTGGCCAAAATTACAACATTCGATCATACAATACATCAGGACCTGCGTACAATGTCAACTAATAAAATCACACCGACCACGCTTACATGGACTATTACAACCACTCCCTATAGCAGAAGGAAGATGGCTTGATATATCAATGGATTTTGTGACAGGATTACCCCCGACATCAAATAACTTGAATATGATCCTCGTCGTAGTTGATCGTTTTTCGAAACGCGCTCACTTCATAGCTACAAGGAAAACCTTAGACGCAACACAACTAATAGATCTACTCTTTCGATACATTTTTTCATATCATGGTTTTCCCAGGACAATAACCAGTGATAGAGATGTCCGTATGACCGCCGACAAATATCAAGAACTCACGAAAAGACTAGGAATAAAATCGACAATGTCTTCCGCGAACCACCCCCAAACAGATGGACAATCCGAACGAACGATACAGACATTAAACAGGTTACTAAGAGCCTATGCTTCAACCAATATTCAGAATTGGCATGTATATTTACCACAAATCGAATTTGTTTACAATTCTACACCTACTAGAACACTTGGAAAATCACCATTTGAAATTGATTTAGGATATTTACCGAATACCCCTGCTATTAAGTCAGATGACGAAGTCAACGCAAGAAGTTTTACTGCCGTAGAACTTGCCAAACACCTCAAAGCCCTTACCATCCAAACGAAGGAACAGCTAGAACACGCTCAAATCGAAATGGAAACTAATAACAATCAAAGACGTAAACCCTTATTGTTAAACATAGGAGATCACGTATTAGTGCATAGAGATGCATACTTCAAGAAAGGTGCTTATATGAAAGTACAACAAATATACGTCGGACCATTTCGAGTTGTCAAGAAAATAAACGATAACGCCTACGAACTAGATTTAAACTCTCACAAGAAAAAGCACAGAGTTATTAATGTACAATTCCTGAAAAAGTTTGTATACCGTCCAGACGCGTACCCAAAGAATAAACCAATCAGCTCCACTGAAAGAATTAAGAGAGCACACGAAGTTACTGCACTCATAGGAATAGATACTACACACAAAACTTACTTATGTCACATGCAAGATGTAGACCCAACACTTTCAGTAGAATACTCAGAAGCTGAATTTTGCCAAATTCCCGAAAGAACACGAAGATCAATATTAGCCAACTTTAGACAACTCTACGAAACACAAGACAACCCTGAGAGAGAGGAAGATGTTGTATCTCAAAATGAGATATGTCAGTATGACAATACGTCACCCTGAACGTTCATAAAACACATATGAAACAACCTTATAACAAAACGAACAACATGAGACAAAACCCGACCTTCCCTAGCTGAACTACCCAAAGTATAAATGCCTGAACAATTAGTTTAGATCCGAGATTCCGCGCTTCCACCACTTAGTATGATTCATATTTTATATAATATATAAGATAAGTAACATTCCGTGAATTAATCTGATAAACTGTTTTGACAACTGGTTACTTCCCTAAGACTGTTTATATTAGGATTGTCAAGACACTCCGGTATTACTCGAGCCCGTAATACAACA**GAAAGTTCCATTTTGGATGCTCTATTTATGGGAATATGACTATACGAATGTCACCCACGATGGCCGGAGTAGCTGTGATGAGCATCTTTTTGTCCTGGGCGGCTTAAAACGTGAGCAAAATTGTACTGTAAATTACTTACGTTATCAATGGAAGTGTGTGAATTTCGTGATAAAATTATTGTCAGAATAGTGACTTCATTATTCTTTATAACTAACCATTAGGATCATATAAGTAATCAGATGCAGTTCGTCATTAATATGTCAGGATGGATAACCATAGGTAAGCACGTGATTATGGAAGGACAGTAAAGTTACCGATCTACTTTTTTGAATGATCAACTTGGTGTTTTCATCTATAAGGATATGGGTCGTTACATTGAAACTATAGTAAAAGATCTCGTTATTGGTATATCGGTCATAATGAAGATGAAATAGTCGGTATGTATTTTTATACCCCCCCCTATATGGTAAAAAGGTTAAGATTCATATTTAAATCTTCATTAATACCACCTCTAATTTCTAATTACCAACATTTTTAACTCCACTTTTATTATATGCTATATACAACATTATGTAAAAGGAGTATACCAAAAATTCTCCTTCATAATATAGGAATTCCACGGACTATAGACTATACTAGTATACTCCGTCTACTGTACGATACACTTCCGCTCAGGTCCTTGTCCTTTAACGAGGCCTTACCACTCTTTTGTTACTCTATTGATCCAGCTCAGCAAAGGCAGTGTGATCTAAGATTCTATCTTCGCGATGTAGTAAAACTAGCTAGACCGAGAAAGAGACTAGAAATGCAAAAGGCACTTCTACAATGGCTGCCATCATTATTATCCGATGTGACGCTGCAGCTTCTCAATGATATTCGAATACGCTTTGAGGAGATACAGCCTAATATCCGACAAACTGTTTTACAGATTTACGATCGTACTTGTTACCCATCATTGAATTTTGAACATCCGAACCTGGGAGTTTTCCCTGAAACAGATAGTATATTTGAACCTGTATAATAATATATAGTCTAGCGCTTTACGGAAGACAATGTATGTATTTCGGTTCCTGGAGAAACTATTGCATCTATTGCATAGGTAATCTTGCACGTCGCATCCCCGGTTCATTTTCTGCGTTTCCATCTTGCACTTCAATAGCATATCTTTGTTAACGAAGCATCTGTGCTTCATTTTGTAGAACAAAAATGCAACGCGAGAGCGCTAATTTTTCAAACAAAGAATCTGAGCTGCATTTTTACAGAACAGAAATGCAACGCGAAAGCGCTATTTTACCAACGAAGAATCTGTGCTTCATTTTTGTAAAACAAAAATGCAACGCGAGAGCGCTAATTTTTCAAACAAAGAATCTGAGCTGCATTTTTACAGAACAGAAATGCAACGCGAGAGCGCTATTTTACCAACAAAGAATCTATACTTCTTTTTTGTTCTACAAAAATGCATCCCGAGAGCGCTATTTTTCTAACAAAGCATCTTAGATTACTTTTTTTCTCCTTTGTGCGCTCTATAATGCAGTCTCTTGATAACTTTTTGCACTGTAGGTCCGTTAAGGTTAGAAGAAGGCTACTTTGGTGTCTATTTTCTCTTCCATAAAAAAAGCCTGACTCCACTTCCCGCGTTTACTGATTACTAGCGAAGCTGCGGGTGCATTTTTTCAAGATAAAGGCATCCCCGATTATATTCTATACCGATGTGGATTGCGCATACTTTGTGAACAGAAAGTGATAGCGTTGATGATTCTTCATTGGTCAGAAAATTATGAACGGTTTCTTCTATTTTGTCTCTATATACTACGTATAGGAAATGTTTACATTTTCGTATTGTTTTCGATTCACTCTATGAATAGTTCTTACTACAATTTTTTTGTCTAAAGAGTAATACTAGAGATAAACATAAAAAATGTAGAGGTCGAGTTTAGATGCAAGTTCAAGGAGCGAAAGGTGGATGGGTAGGTTATATAGGGATATAGCACAGAGATATATAGCAAAGAGATACTTTTGAGCAATGTTTGTGGAAGCGGTATTCGCAATATTTTAGTAGCTCGTTACAGTCCGGTGCGTTTTTGGTTTTTTGAAAGTGCGTCTTCAGAGCGCTTTTGGTTTTCAAAAGCGCTCTGAAGTTCCTATACTTTCTAGAGAATAGGAACTTCGGAATAGGAACTTCAAAGCGTTTCCGAAAACGAGCGCTTCCGAAAATGCAACGCGAGCTGCGCACATACAGCTCACTGTTCACGTCGCACCTATATCTGCGTGTTGCCTGTATATATATATACATGAGAAGAACGGCATAGTGCGTGTTTATGCTTAAATGCGTACTTATATGCGTCTATTTATGTAGGATGAAAGGTAGTCTAGTACCTCCTGTGATATTATCCCATTCCATGCGGGGTATCGTATGCTTCCTTCAGCACTACCCTTTAGCTGTTCTATATGCTGCCACTCCTCAATTGGATTAGTCTCATCCTTCAATGCTATCATTTCCTTTGATATTGGATCATATGCATAGTACCGAGAAACTAGTGCGAAGTAGTGATCAGGTATTGCTGTTATCTGATGAGTATACGTTGTCCTGGCCACGGCAGAAGCACGCTTATCGCTCCAATTTCCCACAACATTAGTCAACTCCGTTAGGCCCTTCATTGAAAGAAATGAGGTCATCAAATGTCTTCCAATGTGAGATTTTGGGCCATTTTTTATAGCAAAGATTGAATAAGGCGCATTTTTCTTCAAAGCTTTATTGTACGATCTGACTAAGTTATCTTTTAATAATTGGTATTCCTGTTTATTGCTTGAAGAATTGCCGGTCCTATTTACTCGTTTTAGGACTGGTTCAGAATTCGGTCTCCCTATAGTGAGTCGTATTAATTCGATATCCGTAATCATGGTCATAGCTGTTTCCTGTGTGAAATTGTTATCCGCTCACAATTCCACACAACATACGAGCCGGAAGCATAAAGTGTAAAGCCTGGGGTGCCTAATGAGTGAGCTAACTCACATTAATTGCGTTGCGCTCACTGCCCGCTTTCCAGTCGGGAAACCTGTCGTGCCAGCTGCATTAATGAATCGGCCAACGCGCGGGGAGAGGCGGTTTGCGTATTGGGCGCTCTTCCGCTTCCTCGCTCACTGACTCGCTGCGCTCGGTCGTTCGGCTGCGGCGAGCGGTATCAGCTCACTCAAAGGCGGTAATACGGTTATCCACAGAATCAGGGGATAACGCAGGAAAGAACATGTGAGCAAAAGGCCAGCAAAAGGCCAGGAACCGTAAAAAGGCCGCGTTGCTGGCGTTTTTCCATAGGCTCCGCCCCCCTGACGAGCATCACAAAAATCGACGCTCAAGTCAGAGGTGGCGAAACCCGACAGGACTATAAAGATACCAGGCGTTTCCCCCTGGAAGCTCCCTCGTGCGCTCTCCTGTTCCGACCCTGCCGCTTACCGGATACCTGTCCGCCTTTCTCCCTTCGGGAAGCGTGGCGCTTTCTCATAGCTCACGCTGTAGGTATCTCAGTTCGGTGTAGGTCGTTCGCTCCAAGCTGGGCTGTGTGCACGAACCCCCCGTTCAGCCCGACCGCTGCGCCTTATCCGGTAACTATCGTCTTGAGTCCAACCCGGTAAGACACGACTTATCGCCACTGGCAGCAGCCACTGGTAACAGGATTAGCAGAGCGAGGTATGTAGGCGGTGCTACAGAGTTCTTGAAGTGGTGGCCTAACTACGGCTACACTAGAAGGACAGTATTTGGTATCTGCGCTCTGCTGAAGCCAGTTACCTTCGGAAAAAGAGTTGGTAGCTCTTGATCCGGCAAACAAACCACCGCTGGTAGCGGTGGTTTTTTTGTTTGCAAGCAGCAGATTACGCGCAGAAAAAAAGGATCTCAAGAAGATCCTTTGATCTTTTCTACGGGGTCTGACGCTCAGTGGAACGAAAACTCACGTTAAGGGATTTTGGTCATGAGATTATCAAAAAGGATCTTCACCTAGATCCTTTTAAATTAAAAATGAAGTTTTAAATCAATCTAAAGTATATATGAGTAAACTTGGTCTGACAGTTACCAATGCTTAATCAGTGAGGCACCTATCTCAGCGATCTGTCTATTTCGTTCATCCATAGTTGCCTGACTCCCCGTCGTGTAGATAACTACGATACGGGAGGGCTTACCATCTGGCCCCAGTGCTGCAATGATACCGCGAGACCCACGCTCACCGGCTCCAGATTTATCAGCAATAAACCAGCCAGCCGGAAGGGCCGAGCGCAGAAGTGGTCCTGCAACTTTATCCGCCTCCATCCAGTCTATTAATTGTTGCCGGGAAGCTAGAGTAAGTAGTTCGCCAGTTAATAGTTTGCGCAACGTTGTTGCCATTGCTACAGGCATCGTGGTGTCACGCTCGTCGTTTGGTATGGCTTCATTCAGCTCCGGTTCCCAACGATCAAGGCGAGTTACATGATCCCCCATGTTGTGCAAAAAAGCGGTTAGCTCCTTCGGTCCTCCGATCGTTGTCAGAAGTAAGTTGGCCGCAGTGTTATCACTCATGGTTATGGCAGCACTGCATAATCCTCTTACTGTCATGCCATCCGTAAGATGCTTTTCTGTGACTGGTGAGTACTCAACCAAGTCATTCTGAGAATAGTGTATGCGGCGACCGAGTTGGTCTTGCCCGGCGTCAATACGGGATAATACCGCGCCACATAGCAGAACTTTAAAAGTGCTCATCATTGGAAAACGTTCTTCGGGGCGAAAACTCTCAAGGATCTTACCGCTGTTTAGATCCAGTTCGATGTAACCCACTCGTGCACCCAACTGATCTTCAGCATCTTTTACTTTCACCAGCGTTTCTGGGTGAGCAAAAACAGGAAGGCAAAATGCCGCAAAAAAGGGAATAAGGGCGACACGGAAATGTTGAATACTCATACTCTTCCTTTTTCAATATTATTGAAGCATTTATCAGGGTTATTGTCTCATGAGCGGATACATATTTGAATGTATTTAGAAAAATAAACAAATAGGGGTTCCGCGCACATTTCCCCGAAAAGTGCCACCTGACGTCTAAGAAACCATTATTATCATGACATTAACCTATAAAAATAGGCGTATCACGAGGCCCTTTCGTCTCGCGCGTTTCGGTGATGACGGTGAAAACCTCTGACACATGCAGCTCCCGGAGACGGTCACAGCTTGTCTGTAAGCGGATGCCGGGAGCAGACAAGCCCGTCAGGGTGCGTCAGCGGGTGTTGGCGGGTGTCGGGGCTGGCTTAACTATGCGGCATCAGAGCAGATTGTACTGAGAGTGCACCATATGCGGTGTGAAATACCGCACAGATGCGTAAGGAGAAAATACCGCATCAGGAANTTCCAGACGATTGAGCGTCAAAATGTAGGTATTTCCATGAGCGTTTTTCCTGTTGCAATGGCTGGCGGTAATATTGTTCTGGATATTACCAGCAAGGCCGATAGTTTGAGTTCTTCTACTCAGGCAAGTGATGTTATTACTAATCAAAGAAGTATTGCGACAACGGTTAATTTGCGTGATGGACAGACTCTTTTACTCGGTGGCCTCACTGATTATAAAAACACTTCTCAGGATTCTGGCGTACCGTTCCTGTCTAAAATCCCTTTAATCGGCCTCCTGTTTAGCTCCCGCTCTGATTCTAACGAGGAAAGCACGTTATACGTGCTCGTCAAAGCAACCATAGTACGCGCCCTGTAGCGGCGCATTAAGCGCGGCGGGTGTGGTGGTTACGCGCAGCGTGACCGCTACACTTGCCAGCGCCCTAGCGCCCGCTCCTTTCGCTTTCTTCCCCTCCTTTGTCGCCACGTTCGCCGGCTTTCCCCGTCAAGCTCTAAATCGGGGGCTCCCTTTAGGGTTCCGATTTAGTGCTTTACGGCACCTCGACCCCAAAAAACTTGATTAGGGTGATGGTTCACGTAGTGGGCCATCGCCCTGATAGACGGTTTTTCGCCCTTTGACGTTGGAGTCCACGTTCTTTAATAGTGGACTCTTGTTCCAAACTGGAACAACACTCAACCCTATCTCGGTCTATTCTTTTGATTTATAAGGGATTTTGCCGATTTCGGCCTATTGGTTAAAAAATGAGCTGATTTAACAAAAATTTAACGCGAATTTTAACAAAATATTAACGTTTACAATTTAAATATTTGCTTATACAATCTTCCTGTTTTTGGGGCTTTTCTGATTATCAACCGGGGTACATATGATTGACATGCTAGTTTTACGATTACCGTTCATCGATTCTCTTGTTTGCTCCAGACTCTCAGGCAATGACCTGATAGCCTTTGTAGACCTCTCAAAAATAGCTACCCTCTCCGGCATGAATTTATCAGCTAGAACGGTTGAATATCATATTGATGGTGATTTGACTGTCTCCGGCCTTTCTCACCCGTTTGAATCTTTACCTACACATTACTCAGGCATTGCATTTAAAATATATGAGGGTTCTAAAAATTTTTATCCTTGCGTTGAAATAAAGGATTCTCCCGCAAAAGTATTACAGGGTCATAATGTTTTTGGTACAACCGATTTAGCTTTATGCTCTGAGGCTTTATTGCTTAATTTTGCTAATTCTTTGCCTTGCCTGTATGATTTATTGGATGTTGGAANTTCCATTCGCCCTTCAGGCTGCGCAACTGTTGGGAAGGGCGATCGGTGCGGGCCTCTTCGCTATTACGCCAGCTGGCGAAAGGGGGATGTGCTGCAAGGCGATTAAGTTGGGTAACGCCAGGGTTTTCCCAGTCACGACGTTGTAAAACGACGGCCAGTGCC

pVB3011

Contents in bold: L-BC GAG

Genetic markers: URA3, 2µ, Amp^R^

Construction: L-BC cDNA was prepared using AMV reverse transcriptase and template RNA isolated from BY4741 (Open Biosystems). L-BC *GAG* was amplified from the cDNA using primers VB1762 (F5- cactatagggaatattaagcttggtaccaaccggaattatgtcgtctctgttaaattc-3) and VB1763 (R5- ccagcacactggcggccgttactagtggatccctattctatatccggtggaagttcgtcctctcc-3) with *Kpn*I and *Bam*HI restriction sites added (underlined), and cloned into *Kpn*I-*Bam*HI digested pYES 2.0 (Invitrogen).

ACGGATTAGAAGCCGCCGAGCGGGTGACAGCCCTCCGAAGGAAGACTCTCCTCCGTGCGTCCTCGTCTTCACCGGTCGCGTTCCTGAAACGCAGATGTGCCTCGCGCCGCACTGCTCCGAACAATAAAGATTCTACAATACTAGCTTTTATGGTTATGAAGAGGAAAAATTGGCAGTAACCTGGCCCCACAAACCTTCAAATGAACGAATCAAATTAACAACCATAGGATGATAATGCGATTAGTTTTTTAGCCTTATTTCTGGGGTAATTAATCAGCGAAGCGATGATTTTTGATCTATTAACAGATATATAAATGCAAAAACTGCATAACCACTTTAACTAATACTTTCAACATTTTCGGTTTGTATTACTTCTTATTCAAATGTAATAAAAGTATCAACAAAAAATTGTTAATATACCTCTATACTTTAACGTCAAGGAGAAAAAACCCCGGATCGGACTACTAGCAGCTGTAATACGACTCACTATAGGGAATATTAAGCTT**GGTACCAACCGGAATTATGTCGTCTCTGTTAAATTCATTACTACCAGAATATTTTAAACCTAAAACTAATTTGAATATCAACTCTTCTAGGGTCCAATATGGCTTTAATGCTCGCATTGATATGCAGTATGAAGACGATAGTGGGACTAGAAAAGGCTCAAGACCCAATGCATTTATGTCTAACACAGTTGCTTTTATAGGAAACTATGAAGGTATTATTGTTGATGACATTCCGATATTGGATGGTCTTAGGGCCGACATTTTTGATACTCATGGTGACTTAGACATGGGCCTCGTTGAGGATGCATTGTCTAAGAGTACCATGATTAGAAGGAATGTACCAACTTATACTGCTTACGCGAGTGAATTACTGTATAAGAGAAACCTTACATCTCTATTTTACAATATGCTCCGTTTATACTACATTAAAAAATGGGGCAGTATTAAGTATGAAAAAGATGCCATCTTTTATGATAATGGCCACGCCTGTCTTTTAAACAGGCAATTGTTTCCAAAGTCTCGTGATGCTTCTTTGGAATCAAGCCTCTCTTTACCTGAGGCTGAAATTGCAATGCTTGATCCTGGCCTGGAATTTCCAGAAGAGGATGTGCCTGCAATTTTATGGCACGGCAGAGTGTCATCCAGAGCAACGTGTATCTTAGGGCAAGCTTGCTCAGAGTTCGCGCCTCTGGCCCCCTTTTCGATTGCGCATTATTCACCACAATTGACGAGAAAACTATTTGTCAATGCGCCCGCTGGGATTGAGCCTAGCTCCGGGCGGTATACTCACGAGGATGTAAAAGATGCGATTACGATCCTTGTGTCTGCAAACCAGGCTTATACTGACTTTGAGGCAGCATACTTGATGCTTGCTCAAACGTTGGTCTCACCTGTACCACGCACTGCCGAAGCAAGTGCATGGTTCATCAATGCTGGCATGGTCAATATGCCAACTTTGTCATGTGCAAATGGTTATTATCCAGCACTGACCAATGTCAATCCTTACCACCGGCTAGACACATGGAAAGATACGTTAAATCATTGGGTGGCTTATCCCGACATGCTGTTTTACCATTCAGTGGCAATGATTGAGAGCTGCTATGTTGAACTCGGGAATGTGGCTCGTGTGTCAGACAGTGATGCAATAAACAAATACACTTTCACTGAGCTATCAGTGCAAGGACGGCCTGTTATGAATCGAGGAATTATTGTAGATCTGACACTTGTGGCAATGCGTACTGGTAGGGAGATCTCACTACCTTACCCGGTCAGCTGTGGCCTGACCCGTACAGACGCGTTATTGCAAGGTACTGAGATTCACGTTCCAGTTGTTGTCAAAGATATTGACATGCCCCAGTATTACAACGCGATTGATAAGGATGTTATTGAGGGGCAGGAAACTGTGATTAGAGTGAAACAGCTGCCACCAGCTATGTATCCAATTTATACTTACGGCATCAACACTACTGAATTCTATTCTGACCATTTTGAAGACCAGGTACAAGTTGAAATGGCACCAATCGATAATGGAAAAGCAGTTTTTAACGATGCAAGAAAGTTTTCGAAATTTATGTCCATAATGCGCATGATGGGGAATGATGTTACTGCTACTGATTTAGTTACAGGTAGAAAAGTGTCGAATTGGGCCGACAACTCATCAGGGCGTTTCTTGTACACGGATGTGAAGTATGAAGGACAAACTGCCTTTTTGGTTGATATGGATACTGTCAAGGCGAGAGACCACTGTTGGGTGTCAATTGTTGATCCTAATGGTACAATGAACTTGTCATATAAGATGACCAATTTTAGAGCAGCGATGTTTTCTAGAAACAAGCCCTTGTATATGACAGGGGGGTCAGTCAGGACCATAGCTACTGGCAATTATCGAGATGCTGCTGAAAGATTGCGTGCAATGGATGAAACGCTCAGATTAAAACCTTTTAAGATTACTGAGAAGTTGGATTTTCGTGTAGCAGCTTACGCGATACCAAGTTTGTCGGGCAGCAATATGCCATCCTTACACCATCAGGAACAACTACAGATATCAGAAGTGGACGCGGAACCAATCAATCCTATAGGAGAGGACGAACTTCCACCGGATATAGAATAGGGATCC**ACTAGTAACGGCCGCCAGTGTGCTGGAATTCTGCAGATATCCATCACACTGGCGGCCGCTCGAGCATGCATCTAGAGGGCCGCATCATGTAATTAGTTATGTCACGCTTACATTCACGCCCTCCCCCCACATCCGCTCTAACCGAAAAGGAAGGAGTTAGACAACCTGAAGTCTAGGTCCCTATTTATTTTTTTATAGTTATGTTAGTATTAAGAACGTTATTTATATTTCAAATTTTTCTTTTTTTTCTGTACAGACGCGTGTACGCATGTAACATTATACTGAAAACCTTGCTTGAGAAGGTTTTGGGACGCTCGAAGGCTTTAATTTGCGGCCCTGCATTAATGAATCGGCCAACGCGCGGGGAGAGGCGGTTTGCGTATTGGGCGCTCTTCCGCTTCCTCGCTCACTGACTCGCTGCGCTCGGTCGTTCGGCTGCGGCGAGCGGTATCAGCTCACTCAAAGGCGGTAATACGGTTATCCACAGAATCAGGGGATAACGCAGGAAAGAACATGTGAGCAAAAGGCCAGCAAAAGCCCAGGAACCGTAAAAAGGCCGCGTTGCTGGCGTTTTTCCATAGGCTCCGCCCCCCTGACGAGCATCACAAAAATCGACGCTCAAGTCAGAGGTGGCGAAACCCGACAGGACTATAAAGATACCAGGCGTTTCCCCCTGGAAGCTCCCTCGTGCGCTCTCCTGTTCCGACCCTGCCGCTTACCGGATACCTGTCCGCCTTTCTCCCTTCGGGAAGCGTGGCGCTTTCTCATAGCTCACGCTGTAGGTATCTCAGTTCGGTGTAGGTCGTTCGCTCCAAGCTGGGCTGTGTGCACGAACCCCCCGTTCAGCCCGACCGCTGCGCCTTATCCGGTAACTATCGTCTTGAGTCCAACCCGGTAAGACACGACTTATCGCCACTGGCAGCAGCCACTGGTAACAGGATTAGCAGAGCGAGGTATGTAGGCGGTGCTACAGAGTTCTTGAAGTGGTGGCCTAACTACGGCTACACTAGAAGGACAGTATTTGGTATCTGCGCTCTGCTGAAGCCAGTTACCTTCGGAAAAAGAGTTGGTAGCTCTTGATCCGGCAAACAAACCACCGCTGGTAGCGGTGGTTTTTTTGTTTGCAAGCAGCAGATTACGCGCAGAAAAAAAGGATCTCAAGAAGATCCTTTGATCTTTTCTACGGGGTCTGACGCTCAGTGGAACGAAAACTCACGTTAAGGGATTTTGGTCATGAGATTATCAAAAAGGATCTTCACCTAGATCCTTTTAAATTAAAAATGAAGTTTTAAATCAATCTAAAGTATATATGAGTAAACTTGGTCTGACAGTTACCAATGCTTAATCAGTGAGGCACCTATCTCAGCGATCTGTCTATTTCGTTCATCCATAGTTGCCTGACTCCCCGTCGTGTAGATAACTACGATACGGGAGCGCTTACCATCTGGCCCCAGTGCTGCAATGATACCGCGAGACCCACGCTCACCGGCTCCAGATTTATCAGCAATAAACCAGCCAGCCGGAAGGGCCGAGCGCAGAAGTGGTCCTGCAACTTTATCCGCCTCCATTCAGTCTATTAATTGTTGCCGGGAAGCTAGAGTAAGTAGTTCGCCAGTTAATAGTTTGCGCAACGTTGTTGGCATTGCTACAGGCATCGTGGTGTCACTCTCGTCGTTTGGTATGGCTTCATTCAGCTCCGGTTCCCAACGATCAAGGCGAGTTACATGATCCCCCATGTTGTGCAAAAAAGCGGTTAGCTCCTTCGGTCCTCCGATCGTTGTCAGAAGTAAGTTGGCCGCAGTGTTATCACTCATGGTTATGGCAGCACTGCATAATTCTCTTACTGTCATGCCATCCGTAAGATGCTTTTCTGTGACTGGTGAGTACTCAACCAAGTCATTCTGAGAATAGTGTATGCGGCGACCGAGTTGCTCTTGCCCGGCGTCAATACGGGATAATAGTGTATCACATAGCAGAACTTTAAAAGTGCTCATCATTGGAAAACGTTCTTCGGGGCGAAAACTCTCAAGGATCTTACCGCTGTTGAGATCCAGTTCGATGTAACCCACTCGTGCACCCAACTGATCTTCAGCATCTTTTACTTTCACCAGCGTTTCTGGGTGAGCAAAAACAGGAAGGCAAAATGCCGCAAAAAAGGGAATAAGGGCGACACGGAAATGTTGAATACTCATACTCTTCCTTTTTCAATGGGTAATAACTGATATAATTAAATTGAAGCTCTAATTTGTGAGTTTAGTATACATGCATTTACTTATAATACAGTTTTTTAGTTTTGCTGGCCGCATCTTCTCAAATATGCTTCCCAGCCTGCTTTTCTGTAACGTTCACCCTCTACCTTAGCATCCCTTCCCTTTGCAAATAGTCCTCTTCCAACAATAATAATGTCAGATCCTGTAGAGACCACATCATCCACGGTTCTATACTGTTGACCCAATGCGTCTCCCTTGTCATCTAAACCCACACCGGGTGTCATAATCAACCAATCGTAACCTTCATCTCTTCCACCCATGTCTCTTTGAGCAATAAAGCCGATAACAAAATCTTTGTCGCTCTTCGCAATGTCAACAGTACCCTTAGTATATTCTCCAGTAGATAGGGAGCCCTTGCATGACAATTCTGCTAACATCAAAAGGCCTCTAGGTTCCTTTGTTACTTCTTCTGCCGCCTGCTTCAAACCGCTAACAATACCTGGGCCCACCACACCGTGTGCATTCGTAATGTCTGCCCATTCTGCTATTCTGTATACACCCGCAGAGTACTGCAATTTGACTGTATTACCAATGTCAGCAAATTTTCTGTCTTCGAAGAGTAAAAAATTGTACTTGGCGGATAATGCCTTTAGCGGCTTAACTGTGCCCTCCATGGAAAAATCAGTCAAGATATCCACATGTGTTTTTAGTAAACAAATTTTGGGACCTAATGCTTCAACTAACTCCAGTAATTCCTTGGTGGTACGAACATCCAATGAAGCACACAAGTTTGTTTGCTTTTCGTGCATGATATTAAATAGCTTGGCAGCAACAGGACTAGGATGAGTAGCAGCACGTTCCTTATATGTAGCTTTCGACATGATTTATCTTCGTTTCCTGCAGGTTTTTGTTCTGTGCAGTTGGGTTAAGAATACTGGGCAATTTCATGTTTCTTCAACACTACATATGCGTATATATACCAATCTAAGTCTGTGCTCCTTCCTTCGTTCTTCCTTCTGTTCGGAGATTACCGAATCAAAAAAATTTCAAAGAAACCGAAATCAAAAAAAAGAATAAAAAAAAAATGATGAATTGAATTGAAAAGCTAGCTTATCGATGATAAGCTGTCAAAGATGAGAATTAATTCCACGGACTATAGACTATACTAGATACTCCGTCTACTGTACGATACACTTCCGCTCAGGTCCTTGTCCTTTAACGAGGCCTTACCACTCTTTTGTTACTCTATTGATCCAGCTCAGCAAAGGCAGTGTGATCTAAGATTCTATCTTCGCGATGTAGTAAAACTAGCTAGACCGAGAAAGAGACTAGAAATGCAAAAGGCACTTCTACAATGGCTGCCATCATTATTATCCGATGTGACGCTGCAGCTTCTCAATGATATTCGAATACGCTTTGAGGAGATACAGCCTAATATCCGACAAACTGTTTTACAGATTTACGATCGTACTTGTTACCCATCATTGAATTTTGAACATCCGAACCTGGGAGTTTTCCCTGAAACAGATAGTATATTTGAACCTGTATAATAATATATAGTCTAGCGCTTTACGGAAGACAATGTATGTATTTCGGTTCCTGGAGAAACTATTGCATCTATTGCATAGGTAATCTTGCACGTCGCATCCCCGGTTCATTTTCTGCGTTTCCATCTTGCACTTCAATAGCATATCTTTGTTAACGAAGCATCTGTGCTTCATTTTGTAGAACAAAAATGCAACGCGAGAGCGCTAATTTTTCAAACAAAGAATCTGAGCTGCATTTTTACAGAACAGAAATGCAACGCGAAAGCGCTATTTTACCAACGAAGAATCTGTGCTTCATTTTTGTAAAACAAAAATGCAACGCGACGAGAGCGCTAATTTTTCAAACAAAGAATCTGAGCTGCATTTTTACAGAACAGAAATGCAACGCGAGAGCGCTATTTTACCAACAAAGAATCTATACTTCTTTTTTGTTCTACAAAAATGCATCCCGAGAGCGCTATTTTTCTAACAAAGCATCTTAGATTACTTTTTTTCTCCTTTGTGCGCTCTATAATGCAGTCTCTTGATAACTTTTTGCACTGTAGGTCCGTTAAGGTTAGAAGAAGGCTACTTTGGTGTCTATTTTCTCTTCCATAAAAAAAGCCTGACTCCACTTCCCGCGTTTACTGATTACTAGCGAAGCTGCGGGTGCATTTTTTCAAGATAAAGGCATCCCCGATTATATTCTATACCGATGTGGATTGCGCATACTTTGTGAACAGAAAGTGATAGCGTTGATGATTCTTCATTGGTCAGAAAATTATGAACGGTTTCTTCTATTTTGTCTCTATATACTACGTATAGGAAATGTTTACATTTTCGTATTGTTTTCGATTCACTCTATGAATAGTTCTTACTACAATTTTTTTGTCTAAAGAGTAATACTAGAGATAAACATAAAAAATGTAGAGGTCGAGTTTAGATGCAAGTTCAAGGAGCGAAAGGTGGATGGGTAGGTTATATAGGGATATAGCACAGAGATATATAGCAAAGAGATACTTTTGAGCAATGTTTGTGGAAGCGGTATTCGCAATGGGAAGCTCCACCCCGGTTGATAATCAGAAAAGCCCCAAAAACAGGAAGATTGTATAAGCAAATATTTAAATTGTAAACGTTAATATTTTGTTAAAATTCGCGTTAAATTTTTGTTAAATCAGCTCATTTTTTAACGAATAGCCCGAAATCGGCAAAATCCCTTATAAATCAAAAGAATAGACCGAGATAGGGTTGAGTGTTGTTCCAGTTTCCAACAAGAGTCCACTATTAAAGAACGTGGACTCCAACGTCAAAGGGCGAAAAAGGGTCTATCAGGGCGATGGCCCACTACGTGAACCATCACCCTAATCAAGTTTTTTGGGGTCGAGGTGCCGTAAAGCAGTAAATCGGAAGGGTAAACGGATGCCCCCATTTAGAGCTTGACGGGGAAAGCCGGCGAACGTGGCGAGAAAGGAAGGGAAGAAAGCGAAAGGAGCGGGGGCTAGGGCGGTGGGAAGTGTAGGGGTCACGCTGGGCGTAACCACCACACCCGCCGCGCTTAATGGGGCGCTACAGGGCGCGTGGGGATGATCCACTAGT

pDM3193

Contents: Ty3 in bold with *his3AI* underlined

Genetic markers: *URA3*, 2µ, Amp^R^

Construction: The *Xho*I fragment containing Ty3-*his3A*I from pTM218 (gift from T. Menees) was inserted in *Xho*I-digested pEUTy3-1 (Kinsey PT, Sandmeyer SB. Ty3 transposes in mating populations of yeast: a novel transposition assay for Ty3. Genetics. 1995; 139: 81-94).

AAGCTTGTTATTATTTAATAAAAGATAAAAGTCACTTGTATGAAATGAAGCAGTTATATATATGTTAATAAGAAAAAGGCAAAAACTTAACCCTAGCAAAAAAGGTAATCAATGTGCTAAAACGGTAAATATTTAAACGCAGGTTGCGAATTGCCAATTGCCAATTGTAGGATACACAGGGAGAGTAGAAACAACCAAGTCCTGTGTCCTGTGGTAGACCCACTTGTTTTACCGCAGAGGGCCCTTTTTTTGTTTACCGTCGCAAAAAAAAAACGCGAAGAATACGCGTTGGTCGTTAAATTTTGTGGTACGCGTAAAACTGCTATTTGGTATCCTCTTTAGGGACGCGCCCATTGTCTATACGGTAATATAGGGCTTTCTTCATTACCTGCTTGCGTCAATTAGCTTTACTCATGTAACACATAATAACCTCTTACATTGTAATGAGGCCCAAAAAAAAAAAAAGAACATCTTCAAAAAAAAAAAACTTTCATGGAAGGACCACCTAGTTAATAAAAAGCTCGCACTCAGGATCGAACTAAGGACCAACAGATTTGCAATCTGCTGCGCTACCACTGCGCCATACGAGCTTGATTTTCTGAAAG**TGTTGTATCTCAAAATGAGATATGTCAGTATGACAATACGTCACCCTGAACGTTCATAAAACACATATGAAACAACCTTATAACAAAACGAACAACATGAGACAAAACCCGACCTTCCCTAGCTGAACTACCCAAAGTATAAATGCCTGAACAATTAGTTTAGATCCGAGATTCCGCGCTTCCACCACTTAGTATGATTCATATTTTATATAATATATAAGATAAGTAACATTCCGTGAATTAATCTGATAAACTGTTTTGACAACTGGTTACTTCCCTAAGACTGTTTATATTAGGATTGTCAAGACACTCCGGTATTACTCGAGCCCGTAATACAACACCTGGTAGCGTTAAAGGTTACTAATTGTTCAAACGAACCATCGAAAAGCCGAACCTAGCTACACCACACCCCAGTATGAGCTTTATGGATCAAATCCCAGGAGGAGGAAATTATCCAAAACTCCCAGTAGAATGCCTTCCTAACTTCCCGATCCAACCATCTTTGACCTTCAGAGGTAGAAATGACTCGCATAAACTGAAAAACTTTATCTCCGAAATAATGTTAAACATGTCTATGATATCTTGGCCGAATGATGCCAGTCGTATTGTGTACTGCAGAAGACATTTATTAAACCCCGCTGCTCAGTGGGCTAATGACTTTGTACAAGAACAAGGTATACTTGAAATAACATTCGACACATTCATACAAGGATTATATCAGCATTTCTATAAGCCACCAGATATCAATAAAATCTTTAATGCAATCACGCAACTTTCCGAAGCTAAACTTGGTATTGAGCGTCTCAACCAACGATTCAGAAAGATTTGGGACAGAATGCCACCAGACTTCATGACCGAAAAAGCTGCCATAATGACATATACTAGGCTATTGACAAAGGAAACCTATAATATTGTCAGAATGCACAAACCAGAGACATTAAAAGACGCCATGGAAGAGGCTTACCAGACAACTGCACTAACTGAAAGATTCTTCCCAGGATTCGAACTTGATGCTGATGGAGACACTATCATCGGTGCCACAACCCACTTACAAGAAGAATACGACTCTGACTATGATTCAGAAGATAATCTGACCCAGAATGGATACGTCCATACCGTAAGGACAAGAAGATCTTACAATAAACCAATGTCAAATCATCGAAACAGGAGAAATAACAACCCATCTAGAGAAGAATGTATAAAAAATCGGCTATGCTTCTATTGTAAGAAAGAGGGACATCGCCTGAACGAATGTAGAGCACGTAAGGCGAGTTCTAACCGATCTTGAACTCGAATCAAAAGACCAACAAACTCCTTTTATCAAAACCTTACCAATTGTACACTATATCGCCATCCCCGAGATGGACAATACCGCCGAAAAAACCATAAAAATACAAAACACGAAAGTAAAAACCCTGTTTGACAGTGGATCACCCACGTCATTTATCCGAAGAGATATTGTAGAACTTCTCAAATACGAAATCTACGAGACCCCTCCACTCCGTTTTAGAGGATTCGTAGCCACCAAATCCGCCGTTACATCCGAAGCAGTCACCATTGACCTCAAAATCAATGACCTGCATATAACTTTAGCCGCGTACATACTGGATAACATGGACTACCAATTGTTAATTGGAAATCCAATCTTACGCCGCTACCCGAAAATCCTGCACACAGTACTGAATACCAGAGAGAGCCCCGACTCCTTAAAGCCCAAGACTTATCGCTCCGAAACCGTTAATAACGTTAGAACCTACTCCGCTGGTAATCGTGGTAACCCCAGAAACATAAAACTGTCTTTTGCCCCCACCATTCTCGAAGCAACTGACCCGAAATCCGCTGGTAATCGTGGTGACTCCAGAACCAAAACCCTGTCTCTTGCAACCACTACTCCTGCAGCAATTGACCCGCTTACGACCCTTGATAACCCAGGTAGTACTCAAAGTACATTTGCGCAATTCCCGATACCTGAAGAAGCGAGCATCCTAGAAGAGGATGGAAAATACTCCAACGTTGTCTCAACCATTCAGAGTGTAGAACCTAATGCTACTGATCACAGCAATAAGGACACCTTTTGCACTTTGCCAGTTTGGTTACAACAGAAGTATAGAGAGATCATACGTAATGATCTCCCACCAAGACCTGCCGACATTAATAACATCCCCGTAAAACATGATATTGAAATTAAACCTGGCGCAAGACTACCTCGACTACAGCCATACCATGTTACAGAAAAGAACGAACAAGAAATCAACAAAATAGTTCAAAAACTGCTCGATAACAAGTTCATTGTTCCCTCAAAGTCGCCTTGCAGCTCCCCTGTAGTCCTCGTCCCGAAGAAAGACGGTACCTTCCGACTCTGCGTCGATTACCGCACCCTGAACAAAGCTACCATCTCCGACCCATTCCCATTACCCAGAATCGACAACCTATTGAGCCGTATTGGAAATGCCCAGATATTTACCACGCTAGATTTGCATAGTGGTTACCACCAGATCCCGATGGAACCCAAAGACCGCTACAAAACCGCCTTTGTCACACCATCCGGTAAGTATGAATATACCGTCATGCCATTTGGCTTAGTCAATGCACCTAGTACATTCGCAAGATACATGGCTGATACATTTAGAGACCTGAGATTCGTCAATGTTTACCTTGATGATATATTAATATTCTCCGAATCTCCAGAAGAACATTGGAAACATTTAGACACGGTACTAGAAAGATTAAAGAACGAGAACCTCATTGTTAAGAAGAAAAAATGTAAATTTGCATCTGAAGAAACTGAGTTTTTAGGCTATAGTATTGGAATCCAGAAAATAGCTCCACTACAGCACAAATGTGCAGCAATCCGAGACTTTCCGACGCCTAAAACAGTAAAACAAGCACAGAGATTTTTAGGAATGATTAATTACTACAGACGATTCATTCCAAATTGCTCCAAGATTGCACAGCCAATCCAACTGTTTATTTGTGACAAAAGTCAATGGACAGAAAAACAAGACAAGGCAATTGATAAACTAAAAGACGCCTTGTGTAACTCCCCCGTCCTAGTACCATTCAACAACAAAGCAAACTACCGACTTACAACAGACGCCTCAAAAGACGGCATTGGTGCTGTTCTAGAAGAAGTCGACAACAAGAACAAACTTGTTGGTGTCGTCGGTTACTTCTCTAAATCCTTAGAGAGTGCCCAGAAAAACTATCCTGCTGGCGAATTAGAACTACTTGGAATTATCAAAGCACTCCACCACTTCCGATATATGCTTCACGGAAAGCATTTCACGTTAAGAACAGACCACATTAGTTTGTTATCATTACAAAACAAGAACGAACCCGCACGACGCGTGCAACGCTGGTTAGATGACCTAGCCACATATGACTTCACCTTAGAATACCTAGCTGGACCCAAGAACGTTGTCGCAGATGCCATATCCCGTGCCGTATATACTATAACCCCCGAAACATCCCGACCTATCGACACAGAAAGCTGGAAATCTTACTACAAATCAGACCCATTATGTAGTGCTGTCTTAATTCATATGAAAGAATTGACACAACACAACGTCACACCTGAAGATATGTCAGCCTTCCGTAGTTACCAGAAGAAACTCGAACTATCAGAGACCTTCCGAAAGAATTATTCCCTAGAAGACGAAATGATCTATTACCAAGACCGACTAGTAGTACCAATAAAACAACAGAACGCAGTTATGAGACTATATCATGACCATACCTTATTTGGAGGACATTTTGGTGTAACAGTGACCCTTGCGAAAATCAGCCCAATTTACTATTGGCCAAAATTACAACATTCGATCATACAATACATCAGGACCTGCGTACAATGTCAACTAATAAAATCACACCGACCACGCTTACATGGACTATTACAACCACTCCCTATAGCAGAAGGAAGATGGCTTGATATATCAATGGATTTTGTGACAGGATTACCCCCGACATCAAATAACTTGAATATGATCCTCGTCGTAGTTGATCGTTTTTCGAAACGCGCTCACTTCATAGCTACAAGGAAAACCTTAGACGCAACACAACTAATAGATCTACTCTTTCGATACATTTTTTCATATCATGGTTTTCCCAGGACAATAACCAGTGATAGAGATGTCCGTATGACCGCCGACAAATATCAAGAACTCACGAAAAGACTAGGAATAAAATCGACAATGTCTTCCGCGAACCACCCCCAAACAGATGGACAATCCGAACGAACGATACAGACATTAAACAGGTTACTAAGAGCCTATGCTTCAACCAATATTCAGAATTGGCATGTATATTTACCACAAATCGAATTTGTTTACAATTCTACACCTACTAGAACACTTGGAAAATCACCATTTGAAATTGATTTAGGATATTTACCGAATACCCCTGCTATTAAGTCAGATGACGAAGTCAACGCAAGAAGTTTTACTGCCGTAGAACTTGCCAAACACCTCAAAGCCCTTACCATCCAAACGAAGGAACAGCTAGAACACGCTCAAATCGAAATGGAAACTAATAACAATCAAAGACGTAAACCCTTATTGTTAAACATAGGAGATCACGTATTAGTGCATAGAGATGCATACTTCAAGAAAGGTGCTTATATGAAAGTACAACAAATATACGTCGGACCATTTCGAGTTGTCAAGAAAATAAACGATAACGCCTACGAACTAGATTTAAACTCTCACAAGAAAAAGCACAGAGTTATTAATGTACAATTCCTGAAAAAGTTTGTATACCGTCCAGACGCGTACCCAAAGAATAAACCAATCAGCTCCACTGAAAGAATTAAGAGAGCACACGAAGTTACTGCACTCATAGGAATAGATACTACACACAAAACTTACTTATGTCACATGCAAGATGTAGACCCAACACTTTCAGTAGAATACTCAGAAGCTGAATTTTGCCAAATTCCCGAAAGAACACGAAGATCAATATTAGCCAACTTTAGACAACTCTACGAAACACAAGACAACCCTGAGAGAGAGGAAGATGTTGTATCTCAAAATGAGATATGTCAGTATGACAATACGTCACCCTGAACGTTCATAAAACACATATGAAACAACCTTATAACAAATCGATAAGCTTGGCTGCAGCTTTAAATAATCGGTGTCACTACATAAGAACACCTTTGGTGGAGGGAACATCGTTGGTACCATTGGGCGAGGTGGCTTCTCTTATGGCAACCGCAAGAGCCTTGAACGCACTCTCACTACGGTGATGATCATTCTTGCCTCGCAGACAATCAACGTGGAGGGTAATTCTGCTAGCCTCTGCAAAGCTTTCAAGAAAATGCGGGATCATCTCGCAAGAGAGATCTCCTACTTTCTCCCTTTGCAAACCAAGTTCGACAACTGCGTACGGCCTGTTCGAAAGATCTACCACCGCTCTGGAAAGTGCCTCATCCAAAGGCGCAAATCCTGATCCAAACCTTTTTACTCCACGCACGGCCCCTAGGGCCTCTTTAAAAGCTTGACCGAGAGCAATCCCGCAGTCTTCAGTGGTGTGATGGTCGTCTATGTGTAAGTCACCAATGCACTCAACGATTAGCGACCAGCCGGAATGCTTGGGTATGTTAATATGGACTAAAGGAGGCTTTTCTGCAGGTCGACTCTAGAGGATCCCCGGGTACCGAGCTCGAATTTTTACTAACAAATGGTATTATTTATAACAGCCAGAGCATGTATCATATGGTCCAGAAACCCTATACCTGTGTGGACGTTAATCACTTGCGATTGTGTGGCCTGTTCTGCTACTGCTTCTGCCTCTTTTTCTGGGAAGATCGAGTGCTCTATCGCTAGGGGACCACCCTTTAAAGAGATCGCAATCTGAATCTTGGTTTCATTTGTAATACGCTTTACTAGGGCTTTCTGCTCTGTCATCTTTGCCTTCGTTTATCTTGCCTGCTCATTTTTTAGTATATTCTTCGAAGAAATCACATTACTTTATATAATGTATAATTCATTATGTGATAATGCCAATCGCTAAGAAAAAAAAAGAGTCATCCGCTAGGTGGAAAAAAAAAAATGAAAATCATTACCGAGGCATAAAAAAATATAGAGTGTACTAGAGGCTCCAAGAGTGTACTAGAGGATCCCCGGGCGAGCTCGAATTCCGAGCTTATCGATCAACATGAGACAAAACCCGACCTTCCCTAGCTGAACTACCCAAAGTATAAATGCCTGAACAATTAGTTTAGATCCGAGATTCCGCGCTTCCACCACTTAGTATGATTCATATTTTATATAATATATAAGATAAGTAACATTCCGTGAATTAATCTGATAAACTGTTTTGACAACTGGTTACTTCCCTAAGACTGTTTATATTAGGATTGTCAAGACACTCCGGTATTACTCGAGCCCGTAATACAACA**GAAAGTTCCATTTTGGATGCTCTATTTATGGGAATATGACTATACGAATGTCACCCACGATGGCCGGAGTAGCTGTGATGAGCATCTTTTTGTCCTGGGCGGCTTAAAACGTGAGCAAAATTGTACTGTAAATTACTTACGTTATCAATGGAAGTGTGTGAATTTCGTGATAAAATTATTGTCAGAATAGTGACTTCATTATTCTTTATAACTAACCATTAGGATCATATAAGTAATCAGATGCAGTTCGTCATTAATATGTCAGGATGGATAACCATAGGTAAGCACGTGATTATGGAAGGACAGTAAAGTTACCGATCTACTTTTTTGAATGATCAACTTGGTGTTTTCATCTATAAGGATATGGGTCGTTACATTGAAACTATAGTAAAAGATCTCGTTATTGGTATATCGGTCATAATGAAGATGAAATAGTCGGTATGTATTTTTATACCCCCCCCTATATGGTAAAAAGGTTAAGATTCATATTTAAATCTTCATTAATACCACCTCTAATTTCTAATTACCAACATTTTTAACTCCACTTTTATTATATGCTATATACAACATTATGTAAAAGGAGTATACCAAAAATTCTCCTTCATAATATAGGAATTCGGTCTCCCTATAGTGAGTCGTATTAATTCGATATCCGTAATCATGGTCATAGCTGTTTCCTGTGTGAAATTGTTATCCGCTCACAATTCCACACAACATACGAGCCGGAAGCATAAAGTGTAAAGCCTGGGGTGCCTAATGAGTGAGCTAACTCACATTAATTGCGTTGCGCTCACTGCCCGCTTTCCAGTCGGGAAACCTGTCGTGCCAGCTGCATTAATGAATCGGCCAACGCGCGGGGAGAGGCGGTTTGCGTATTGGGCGCTCTTCCGCTTCCTCGCTCACTGACTCGCTGCGCTCGGTCGTTCGGCTGCGGCGAGCGGTATCAGCTCACTCAAAGGCGGTAATACGGTTATCCACAGAATCAGGGGATAACGCAGGAAAGAACATGTGAGCAAAAGGCCAGCAAAAGGCCAGGAACCGTAAAAAGGCCGCGTTGCTGGCGTTTTTCCATAGGCTCCGCCCCCCTGACGAGCATCACAAAAATCGACGCTCAAGTCAGAGGTGGCGAAACCCGACAGGACTATAAAGATACCAGGCGTTTCCCCCTGGAAGCTCCCTCGTGCGCTCTCCTGTTCCGACCCTGCCGCTTACCGGATACCTGTCCGCCTTTCTCCCTTCGGGAAGCGTGGCGCTTTCTCATAGCTCACGCTGTAGGTATCTCAGTTCGGTGTAGGTCGTTCGCTCCAAGCTGGGCTGTGTGCACGAACCCCCCGTTCAGCCCGACCGCTGCGCCTTATCCGGTAACTATCGTCTTGAGTCCAACCCGGTAAGACACGACTTATCGCCACTGGCAGCAGCCACTGGTAACAGGATTAGCAGAGCGAGGTATGTAGGCGGTGCTACAGAGTTCTTGAAGTGGTGGCCTAACTACGGCTACACTAGAAGGACAGTATTTGGTATCTGCGCTCTGCTGAAGCCAGTTACCTTCGGAAAAAGAGTTGGTAGCTCTTGATCCGGCAAACAAACCACCGCTGGTAGCGGTGGTTTTTTTGTTTGCAAGCAGCAGATTACGCGCAGAAAAAAAGGATCTCAAGAAGATCCTTTGATCTTTTCTACGGGGTCTGACGCTCAGTGGAACGAAAACTCACGTTAAGGGATTTTGGTCATGAGATTATCAAAAAGGATCTTCACCTAGATCCTTTTAAATTAAAAATGAAGTTTTAAATCAATCTAAAGTATATATGAGTAAACTTGGTCTGACAGTTACCAATGCTTAATCAGTGAGGCACCTATCTCAGCGATCTGTCTATTTCGTTCATCCATAGTTGCCTGACTCCCCGTCGTGTAGATAACTACGATACGGGAGGGCTTACCATCTGGCCCCAGTGCTGCAATGATACCGCGAGACCCACGCTCACCGGCTCCAGATTTATCAGCAATAAACCAGCCAGCCGGAAGGGCCGAGCGCAGAAGTGGTCCTGCAACTTTATCCGCCTCCATCCAGTCTATTAATTGTTGCCGGGAAGCTAGAGTAAGTAGTTCGCCAGTTAATAGTTTGCGCAACGTTGTTGCCATTGCTACAGGCATCGTGGTGTCACGCTCGTCGTTTGGTATGGCTTCATTCAGCTCCGGTTCCCAACGATCAAGGCGAGTTACATGATCCCCCATGTTGTGCAAAAAAGCGGTTAGCTCCTTCGGTCCTCCGATCGTTGTCAGAAGTAAGTTGGCCGCAGTGTTATCACTCATGGTTATGGCAGCACTGCATAATCCTCTTACTGTCATGCCATCCGTAAGATGCTTTTCTGTGACTGGTGAGTACTCAACCAAGTCATTCTGAGAATAGTGTATGCGGCGACCGAGTTGGTCTTGCCCGGCGTCAATACGGGATAATACCGCGCCACATAGCAGAACTTTAAAAGTGCTCATCATTGGAAAACGTTCTTCGGGGCGAAAACTCTCAAGGATCTTACCGCTGTTTAGATCCAGTTCGATGTAACCCACTCGTGCACCCAACTGATCTTCAGCATCTTTTACTTTCACCAGCGTTTCTGGGTGAGCAAAAACAGGAAGGCAAAATGCCGCAAAAAAGGGAATAAGGGCGACACGGAAATGTTGAATACTCATACTCTTCCTTTTTCAATATTATTGAAGCATTTATCAGGGTTATTGTCTCATGAGCGGATACATATTTGAATGTATTTAGAAAAATAAACAAATAGGGGTTCCGCGCACATTTCCCCGAAAAGTGCCACCTGACGTCTAAGAAACCATTATTATCATGACATTAACCTATAAAAATAGGCGTATCACGAGGCCCTTTCGTCTCGCGCGTTTCGGTGATGACGGTGAAAACCTCTGACACATGCAGCTCCCGGAGACGGTCACAGCTTGTCTGTAAGCGGATGCCGGGAGCAGACAAGCCCGTCAGGGTGCGTCAGCGGGTGTTGGCGGGTGTCGGGGCTGGCTTAACTATGCGGCATCAGAGCAGATTGTACTGAGAGTGCACCATATGCGGTGTGAAATACCGCACAGATGCGTAAGGAGAAAATACCGCATCAGGAANTTCCAGACGATTGAGCGTCAAAATGTAGGTATTTCCATGAGCGTTTTTCCTGTTGCAATGGCTGGCGGTAATATTGTTCTGGATATTACCAGCAAGGCCGATAGTTTGAGTTCTTCTACTCAGGCAAGTGATGTTATTACTAATCAAAGAAGTATTGCGACAACGGTTAATTTGCGTGATGGACAGACTCTTTTACTCGGTGGCCTCACTGATTATAAAAACACTTCTCAGGATTCTGGCGTACCGTTCCTGTCTAAAATCCCTTTAATCGGCCTCCTGTTTAGCTCCCGCTCTGATTCTAACGAGGAAAGCACGTTATACGTGCTCGTCAAAGCAACCATAGTACGCGCCCTGTAGCGGCGCATTAAGCGCGGCGGGTGTGGTGGTTACGCGCAGCGTGACCGCTACACTTGCCAGCGCCCTAGCGCCCGCTCCTTTCGCTTTCTTCCCCTCCTTTGTCGCCACGTTCGCCGGCTTTCCCCGTCAAGCTCTAAATCGGGGGCTCCCTTTAGGGTTCCGATTTAGTGCTTTACGGCACCTCGACCCCAAAAAACTTGATTAGGGTGATGGTTCACGTAGTGGGCCATCGCCCTGATAGACGGTTTTTCGCCCTTTGACGTTGGAGTCCACGTTCTTTAATAGTGGACTCTTGTTCCAAACTGGAACAACACTCAACCCTATCTCGGTCTATTCTTTTGATTTATAAGGGATTTTGCCGATTTCGGCCTATTGGTTAAAAAATGAGCTGATTTAACAAAAATTTAACGCGAATTTTAACAAAATATTAACGTTTACAATTTAAATATTTGCTTATACAATCTTCCTGTTTTTGGGGCTTTTCTGATTATCAACCGGGGTACATATGATTGACATGCTAGTTTTACGATTACCGTTCATCGATTCTCTTGTTTGCTCCAGACTCTCAGGCAATGACCTGATAGCCTTTGTAGACCTCTCAAAAATAGCTACCCTCTCCGGCATGAATTTATCAGCTAGAACGGTTGAATATCATATTGATGGTGATTTGACTGTCTCCGGCCTTTCTCACCCGTTTGAATCTTTACCTACACATTACTCAGGCATTGCATTTAAAATATATGAGGGTTCTAAAAATTTTTATCCTTGCGTTGAAATAAAGGATTCTCCCGCAAAAGTATTACAGGGTCATAATGTTTTTGGTACAACCGATTTAGCTTTATGCTCTGAGGCTTTATTGCTTAATTTTGCTAATTCTTTGCCTTGCCTGTATGATTTATTGGATGTTGGAANTTCCATTCGCCCTTCAGGCTGCGCAACTGTTGGGAAGGGCGATCGGTGCGGGCCTCTTCGCTATTACGCCAGCTGGCGAAAGGGGGATGTGCTGCAAGGCGATTAAGTTGGGTAACGCCAGGGTTTTCCCAGTCACGACGTTGTAAAACGACGGCCAGTGCC

pDM3194

Contents: Ty3 in bold with *his3AI* underlined; IN catalytic mutations in red

Genetic markers: *URA3*, 2µ, Amp^R^

Construction: The *Xho*I fragment containing Ty3-*his3A*I from pTM216 (gift from T. Menees) was inserted in *Xho*I-digested pEUTy3-1 (Kinsey PT, Sandmeyer SB. Ty3 transposes in mating populations of yeast: a novel transposition assay for Ty3. Genetics. 1995; 139: 81-94).

AAGCTTGTTATTATTTAATAAAAGATAAAAGTCACTTGTATGAAATGAAGCAGTTATATATATGTTAATAAGAAAAAGGCAAAAACTTAACCCTAGCAAAAAAGGTAATCAATGTGCTAAAACGGTAAATATTTAAACGCAGGTTGCGAATTGCCAATTGCCAATTGTAGGATACACAGGGAGAGTAGAAACAACCAAGTCCTGTGTCCTGTGGTAGACCCACTTGTTTTACCGCAGAGGGCCCTTTTTTTGTTTACCGTCGCAAAAAAAAAACGCGAAGAATACGCGTTGGTCGTTAAATTTTGTGGTACGCGTAAAACTGCTATTTGGTATCCTCTTTAGGGACGCGCCCATTGTCTATACGGTAATATAGGGCTTTCTTCATTACCTGCTTGCGTCAATTAGCTTTACTCATGTAACACATAATAACCTCTTACATTGTAATGAGGCCCAAAAAAAAAAAAAGAACATCTTCAAAAAAAAAAAACTTTCATGGAAGGACCACCTAGTTAATAAAAAGCTCGCACTCAGGATCGAACTAAGGACCAACAGATTTGCAATCTGCTGCGCTACCACTGCGCCATACGAGCTTGATTTTCTGAAAG**TGTTGTATCTCAAAATGAGATATGTCAGTATGACAATACGTCACCCTGAACGTTCATAAAACACATATGAAACAACCTTATAACAAAACGAACAACATGAGACAAAACCCGACCTTCCCTAGCTGAACTACCCAAAGTATAAATGCCTGAACAATTAGTTTAGATCCGAGATTCCGCGCTTCCACCACTTAGTATGATTCATATTTTATATAATATATAAGATAAGTAACATTCCGTGAATTAATCTGATAAACTGTTTTGACAACTGGTTACTTCCCTAAGACTGTTTATATTAGGATTGTCAAGACACTCCGGTATTACTCGAGCCCGTAATACAACACCTGGTAGCGTTAAAGGTTACTAATTGTTCAAACGAACCATCGAAAAGCCGAACCTAGCTACACCACACCCCAGTATGAGCTTTATGGATCAAATCCCAGGAGGAGGAAATTATCCAAAACTCCCAGTAGAATGCCTTCCTAACTTCCCGATCCAACCATCTTTGACCTTCAGAGGTAGAAATGACTCGCATAAACTGAAAAACTTTATCTCCGAAATAATGTTAAACATGTCTATGATATCTTGGCCGAATGATGCCAGTCGTATTGTGTACTGCAGAAGACATTTATTAAACCCCGCTGCTCAGTGGGCTAATGACTTTGTACAAGAACAAGGTATACTTGAAATAACATTCGACACATTCATACAAGGATTATATCAGCATTTCTATAAGCCACCAGATATCAATAAAATCTTTAATGCAATCACGCAACTTTCCGAAGCTAAACTTGGTATTGAGCGTCTCAACCAACGATTCAGAAAGATTTGGGACAGAATGCCACCAGACTTCATGACCGAAAAAGCTGCCATAATGACATATACTAGGCTATTGACAAAGGAAACCTATAATATTGTCAGAATGCACAAACCAGAGACATTAAAAGACGCCATGGAAGAGGCTTACCAGACAACTGCACTAACTGAAAGATTCTTCCCAGGATTCGAACTTGATGCTGATGGAGACACTATCATCGGTGCCACAACCCACTTACAAGAAGAATACGACTCTGACTATGATTCAGAAGATAATCTGACCCAGAATGGATACGTCCATACCGTAAGGACAAGAAGATCTTACAATAAACCAATGTCAAATCATCGAAACAGGAGAAATAACAACCCATCTAGAGAAGAATGTATAAAAAATCGGCTATGCTTCTATTGTAAGAAAGAGGGACATCGCCTGAACGAATGTAGAGCACGTAAGGCGAGTTCTAACCGATCTTGAACTCGAATCAAAAGACCAACAAACTCCTTTTATCAAAACCTTACCAATTGTACACTATATCGCCATCCCCGAGATGGACAATACCGCCGAAAAAACCATAAAAATACAAAACACGAAAGTAAAAACCCTGTTTGACAGTGGATCACCCACGTCATTTATCCGAAGAGATATTGTAGAACTTCTCAAATACGAAATCTACGAGACCCCTCCACTCCGTTTTAGAGGATTCGTAGCCACCAAATCCGCCGTTACATCCGAAGCAGTCACCATTGACCTCAAAATCAATGACCTGCATATAACTTTAGCCGCGTACATACTGGATAACATGGACTACCAATTGTTAATTGGAAATCCAATCTTACGCCGCTACCCGAAAATCCTGCACACAGTACTGAATACCAGAGAGAGCCCCGACTCCTTAAAGCCCAAGACTTATCGCTCCGAAACCGTTAATAACGTTAGAACCTACTCCGCTGGTAATCGTGGTAACCCCAGAAACATAAAACTGTCTTTTGCCCCCACCATTCTCGAAGCAACTGACCCGAAATCCGCTGGTAATCGTGGTGACTCCAGAACCAAAACCCTGTCTCTTGCAACCACTACTCCTGCAGCAATTGACCCGCTTACGACCCTTGATAACCCAGGTAGTACTCAAAGTACATTTGCGCAATTCCCGATACCTGAAGAAGCGAGCATCCTAGAAGAGGATGGAAAATACTCCAACGTTGTCTCAACCATTCAGAGTGTAGAACCTAATGCTACTGATCACAGCAATAAGGACACCTTTTGCACTTTGCCAGTTTGGTTACAACAGAAGTATAGAGAGATCATACGTAATGATCTCCCACCAAGACCTGCCGACATTAATAACATCCCCGTAAAACATGATATTGAAATTAAACCTGGCGCAAGACTACCTCGACTACAGCCATACCATGTTACAGAAAAGAACGAACAAGAAATCAACAAAATAGTTCAAAAACTGCTCGATAACAAGTTCATTGTTCCCTCAAAGTCGCCTTGCAGCTCCCCTGTAGTCCTCGTCCCGAAGAAAGACGGTACCTTCCGACTCTGCGTCGATTACCGCACCCTGAACAAAGCTACCATCTCCGACCCATTCCCATTACCCAGAATCGACAACCTATTGAGCCGTATTGGAAATGCCCAGATATTTACCACGCTAGATTTGCATAGTGGTTACCACCAGATCCCGATGGAACCCAAAGACCGCTACAAAACCGCCTTTGTCACACCATCCGGTAAGTATGAATATACCGTCATGCCATTTGGCTTAGTCAATGCACCTAGTACATTCGCAAGATACATGGCTGATACATTTAGAGACCTGAGATTCGTCAATGTTTACCTTGATGATATATTAATATTCTCCGAATCTCCAGAAGAACATTGGAAACATTTAGACACGGTACTAGAAAGATTAAAGAACGAGAACCTCATTGTTAAGAAGAAAAAATGTAAATTTGCATCTGAAGAAACTGAGTTTTTAGGCTATAGTATTGGAATCCAGAAAATAGCTCCACTACAGCACAAATGTGCAGCAATCCGAGACTTTCCGACGCCTAAAACAGTAAAACAAGCACAGAGATTTTTAGGAATGATTAATTACTACAGACGATTCATTCCAAATTGCTCCAAGATTGCACAGCCAATCCAACTGTTTATTTGTGACAAAAGTCAATGGACAGAAAAACAAGACAAGGCAATTGATAAACTAAAAGACGCCTTGTGTAACTCCCCCGTCCTAGTACCATTCAACAACAAAGCAAACTACCGACTTACAACAGACGCCTCAAAAGACGGCATTGGTGCTGTTCTAGAAGAAGTCGACAACAAGAACAAACTTGTTGGTGTCGTCGGTTACTTCTCTAAATCCTTAGAGAGTGCCCAGAAAAACTATCCTGCTGGCGAATTAGAACTACTTGGAATTATCAAAGCACTCCACCACTTCCGATATATGCTTCACGGAAAGCATTTCACGTTAAGAACAGACCACATTAGTTTGTTATCATTACAAAACAAGAACGAACCCGCACGACGCGTGCAACGCTGGTTAGATGACCTAGCCACATATGACTTCACCTTAGAATACCTAGCTGGACCCAAGAACGTTGTCGCAGATGCCATATCCCGTGCCGTATATACTATAACCCCCGAAACATCCCGACCTATCGACACAGAAAGCTGGAAATCTTACTACAAATCAGACCCATTATGTAGTGCTGTCTTAATTCATATGAAAGAATTGACACAACACAACGTCACACCTGAAGATATGTCAGCCTTCCGTAGTTACCAGAAGAAACTCGAACTATCAGAGACCTTCCGAAAGAATTATTCCCTAGAAGACGAAATGATCTATTACCAAGACCGACTAGTAGTACCAATAAAACAACAGAACGCAGTTATGAGACTATATCATGACCATACCTTATTTGGAGGACATTTTGGTGTAACAGTGACCCTTGCGAAAATCAGCCCAATTTACTATTGGCCAAAATTACAACATTCGATCATACAATACATCAGGACCTGCGTACAATGTCAACTAATAAAATCACACCGACCACGCTTACATGGACTATTACAACCACTCCCTATAGCAGAAGGAAGATGGCTTGATATATCAATGGATTTTGTGACAGGATTACCCCCGACATCAAATAACTTGAATATGATCCTCGTCGTAGTTGATCGTTTTTCGAAACGCGCTCACTTCATAGCTACAAGGAAAACCTTAGACGCAACACAACTAATAGATCTACTCTTTCGATACATTTTTTCATATCATGGTTTTCCCAGGACAATAACCAGTGAAAGAGATGTCCGTATGACCGCCGACAAATATCAAGAACTCACGAAAAGACTAGGAATAAAATCGACAATGTCTTCCGCGAACCACCCCCAAACAGATGGACAATCCGATCGAACGATACAGACATTAAACAGGTTACTAAGAGCCTATGCTTCAACCAATATTCAGAATTGGCATGTATATTTACCACAAATCGAATTTGTTTACAATTCTACACCTACTAGAACACTTGGAAAATCACCATTTGAAATTGATTTAGGATATTTACCGAATACCCCTGCTATTAAGTCAGATGACGAAGTCAACGCAAGAAGTTTTACTGCCGTAGAACTTGCCAAACACCTCAAAGCCCTTACCATCCAAACGAAGGAACAGCTAGAACACGCTCAAATCGAAATGGAAACTAATAACAATCAAAGACGTAAACCCTTATTGTTAAACATAGGAGATCACGTATTAGTGCATAGAGATGCATACTTCAAGAAAGGTGCTTATATGAAAGTACAACAAATATACGTCGGACCATTTCGAGTTGTCAAGAAAATAAACGATAACGCCTACGAACTAGATTTAAACTCTCACAAGAAAAAGCACAGAGTTATTAATGTACAATTCCTGAAAAAGTTTGTATACCGTCCAGACGCGTACCCAAAGAATAAACCAATCAGCTCCACTGAAAGAATTAAGAGAGCACACGAAGTTACTGCACTCATAGGAATAGATACTACACACAAAACTTACTTATGTCACATGCAAGATGTAGACCCAACACTTTCAGTAGAATACTCAGAAGCTGAATTTTGCCAAATTCCCGAAAGAACACGAAGATCAATATTAGCCAACTTTAGACAACTCTACGAAACACAAGACAACCCTGAGAGAGAGGAAGATGTTGTATCTCAAAATGAGATATGTCAGTATGACAATACGTCACCCTGAACGTTCATAAAACACATATGAAACAACCTTATAACAAATCGATAAGCTTGGCTGCAGCTTTAAATAATCGGTGTCACTACATAAGAACACCTTTGGTGGAGGGAACATCGTTGGTACCATTGGGCGAGGTGGCTTCTCTTATGGCAACCGCAAGAGCCTTGAACGCACTCTCACTACGGTGATGATCATTCTTGCCTCGCAGACAATCAACGTGGAGGGTAATTCTGCTAGCCTCTGCAAAGCTTTCAAGAAAATGCGGGATCATCTCGCAAGAGAGATCTCCTACTTTCTCCCTTTGCAAACCAAGTTCGACAACTGCGTACGGCCTGTTCGAAAGATCTACCACCGCTCTGGAAAGTGCCTCATCCAAAGGCGCAAATCCTGATCCAAACCTTTTTACTCCACGCACGGCCCCTAGGGCCTCTTTAAAAGCTTGACCGAGAGCAATCCCGCAGTCTTCAGTGGTGTGATGGTCGTCTATGTGTAAGTCACCAATGCACTCAACGATTAGCGACCAGCCGGAATGCTTGGGTATGTTAATATGGACTAAAGGAGGCTTTTCTGCAGGTCGACTCTAGAGGATCCCCGGGTACCGAGCTCGAATTTTTACTAACAAATGGTATTATTTATAACAGCCAGAGCATGTATCATATGGTCCAGAAACCCTATACCTGTGTGGACGTTAATCACTTGCGATTGTGTGGCCTGTTCTGCTACTGCTTCTGCCTCTTTTTCTGGGAAGATCGAGTGCTCTATCGCTAGGGGACCACCCTTTAAAGAGATCGCAATCTGAATCTTGGTTTCATTTGTAATACGCTTTACTAGGGCTTTCTGCTCTGTCATCTTTGCCTTCGTTTATCTTGCCTGCTCATTTTTTAGTATATTCTTCGAAGAAATCACATTACTTTATATAATGTATAATTCATTATGTGATAATGCCAATCGCTAAGAAAAAAAAAGAGTCATCCGCTAGGTGGAAAAAAAAAAATGAAAATCATTACCGAGGCATAAAAAAATATAGAGTGTACTAGAGGCTCCAAGAGTGTACTAGAGGATCCCCGGGCGAGCTCGAATTCCGAGCTTATCGATCAACATGAGACAAAACCCGACCTTCCCTAGCTGAACTACCCAAAGTATAAATGCCTGAACAATTAGTTTAGATCCGAGATTCCGCGCTTCCACCACTTAGTATGATTCATATTTTATATAATATATAAGATAAGTAACATTCCGTGAATTAATCTGATAAACTGTTTTGACAACTGGTTACTTCCCTAAGACTGTTTATATTAGGATTGTCAAGACACTCCGGTATTACTCGAGCCCGTAATACAACA**GAAAGTTCCATTTTGGATGCTCTATTTATGGGAATATGACTATACGAATGTCACCCACGATGGCCGGAGTAGCTGTGATGAGCATCTTTTTGTCCTGGGCGGCTTAAAACGTGAGCAAAATTGTACTGTAAATTACTTACGTTATCAATGGAAGTGTGTGAATTTCGTGATAAAATTATTGTCAGAATAGTGACTTCATTATTCTTTATAACTAACCATTAGGATCATATAAGTAATCAGATGCAGTTCGTCATTAATATGTCAGGATGGATAACCATAGGTAAGCACGTGATTATGGAAGGACAGTAAAGTTACCGATCTACTTTTTTGAATGATCAACTTGGTGTTTTCATCTATAAGGATATGGGTCGTTACATTGAAACTATAGTAAAAGATCTCGTTATTGGTATATCGGTCATAATGAAGATGAAATAGTCGGTATGTATTTTTATACCCCCCCCTATATGGTAAAAAGGTTAAGATTCATATTTAAATCTTCATTAATACCACCTCTAATTTCTAATTACCAACATTTTTAACTCCACTTTTATTATATGCTATATACAACATTATGTAAAAGGAGTATACCAAAAATTCTCCTTCATAATATAGGAATTCGGTCTCCCTATAGTGAGTCGTATTAATTCGATATCCGTAATCATGGTCATAGCTGTTTCCTGTGTGAAATTGTTATCCGCTCACAATTCCACACAACATACGAGCCGGAAGCATAAAGTGTAAAGCCTGGGGTGCCTAATGAGTGAGCTAACTCACATTAATTGCGTTGCGCTCACTGCCCGCTTTCCAGTCGGGAAACCTGTCGTGCCAGCTGCATTAATGAATCGGCCAACGCGCGGGGAGAGGCGGTTTGCGTATTGGGCGCTCTTCCGCTTCCTCGCTCACTGACTCGCTGCGCTCGGTCGTTCGGCTGCGGCGAGCGGTATCAGCTCACTCAAAGGCGGTAATACGGTTATCCACAGAATCAGGGGATAACGCAGGAAAGAACATGTGAGCAAAAGGCCAGCAAAAGGCCAGGAACCGTAAAAAGGCCGCGTTGCTGGCGTTTTTCCATAGGCTCCGCCCCCCTGACGAGCATCACAAAAATCGACGCTCAAGTCAGAGGTGGCGAAACCCGACAGGACTATAAAGATACCAGGCGTTTCCCCCTGGAAGCTCCCTCGTGCGCTCTCCTGTTCCGACCCTGCCGCTTACCGGATACCTGTCCGCCTTTCTCCCTTCGGGAAGCGTGGCGCTTTCTCATAGCTCACGCTGTAGGTATCTCAGTTCGGTGTAGGTCGTTCGCTCCAAGCTGGGCTGTGTGCACGAACCCCCCGTTCAGCCCGACCGCTGCGCCTTATCCGGTAACTATCGTCTTGAGTCCAACCCGGTAAGACACGACTTATCGCCACTGGCAGCAGCCACTGGTAACAGGATTAGCAGAGCGAGGTATGTAGGCGGTGCTACAGAGTTCTTGAAGTGGTGGCCTAACTACGGCTACACTAGAAGGACAGTATTTGGTATCTGCGCTCTGCTGAAGCCAGTTACCTTCGGAAAAAGAGTTGGTAGCTCTTGATCCGGCAAACAAACCACCGCTGGTAGCGGTGGTTTTTTTGTTTGCAAGCAGCAGATTACGCGCAGAAAAAAAGGATCTCAAGAAGATCCTTTGATCTTTTCTACGGGGTCTGACGCTCAGTGGAACGAAAACTCACGTTAAGGGATTTTGGTCATGAGATTATCAAAAAGGATCTTCACCTAGATCCTTTTAAATTAAAAATGAAGTTTTAAATCAATCTAAAGTATATATGAGTAAACTTGGTCTGACAGTTACCAATGCTTAATCAGTGAGGCACCTATCTCAGCGATCTGTCTATTTCGTTCATCCATAGTTGCCTGACTCCCCGTCGTGTAGATAACTACGATACGGGAGGGCTTACCATCTGGCCCCAGTGCTGCAATGATACCGCGAGACCCACGCTCACCGGCTCCAGATTTATCAGCAATAAACCAGCCAGCCGGAAGGGCCGAGCGCAGAAGTGGTCCTGCAACTTTATCCGCCTCCATCCAGTCTATTAATTGTTGCCGGGAAGCTAGAGTAAGTAGTTCGCCAGTTAATAGTTTGCGCAACGTTGTTGCCATTGCTACAGGCATCGTGGTGTCACGCTCGTCGTTTGGTATGGCTTCATTCAGCTCCGGTTCCCAACGATCAAGGCGAGTTACATGATCCCCCATGTTGTGCAAAAAAGCGGTTAGCTCCTTCGGTCCTCCGATCGTTGTCAGAAGTAAGTTGGCCGCAGTGTTATCACTCATGGTTATGGCAGCACTGCATAATCCTCTTACTGTCATGCCATCCGTAAGATGCTTTTCTGTGACTGGTGAGTACTCAACCAAGTCATTCTGAGAATAGTGTATGCGGCGACCGAGTTGGTCTTGCCCGGCGTCAATACGGGATAATACCGCGCCACATAGCAGAACTTTAAAAGTGCTCATCATTGGAAAACGTTCTTCGGGGCGAAAACTCTCAAGGATCTTACCGCTGTTTAGATCCAGTTCGATGTAACCCACTCGTGCACCCAACTGATCTTCAGCATCTTTTACTTTCACCAGCGTTTCTGGGTGAGCAAAAACAGGAAGGCAAAATGCCGCAAAAAAGGGAATAAGGGCGACACGGAAATGTTGAATACTCATACTCTTCCTTTTTCAATATTATTGAAGCATTTATCAGGGTTATTGTCTCATGAGCGGATACATATTTGAATGTATTTAGAAAAATAAACAAATAGGGGTTCCGCGCACATTTCCCCGAAAAGTGCCACCTGACGTCTAAGAAACCATTATTATCATGACATTAACCTATAAAAATAGGCGTATCACGAGGCCCTTTCGTCTCGCGCGTTTCGGTGATGACGGTGAAAACCTCTGACACATGCAGCTCCCGGAGACGGTCACAGCTTGTCTGTAAGCGGATGCCGGGAGCAGACAAGCCCGTCAGGGTGCGTCAGCGGGTGTTGGCGGGTGTCGGGGCTGGCTTAACTATGCGGCATCAGAGCAGATTGTACTGAGAGTGCACCATATGCGGTGTGAAATACCGCACAGATGCGTAAGGAGAAAATACCGCATCAGGAANTTCCAGACGATTGAGCGTCAAAATGTAGGTATTTCCATGAGCGTTTTTCCTGTTGCAATGGCTGGCGGTAATATTGTTCTGGATATTACCAGCAAGGCCGATAGTTTGAGTTCTTCTACTCAGGCAAGTGATGTTATTACTAATCAAAGAAGTATTGCGACAACGGTTAATTTGCGTGATGGACAGACTCTTTTACTCGGTGGCCTCACTGATTATAAAAACACTTCTCAGGATTCTGGCGTACCGTTCCTGTCTAAAATCCCTTTAATCGGCCTCCTGTTTAGCTCCCGCTCTGATTCTAACGAGGAAAGCACGTTATACGTGCTCGTCAAAGCAACCATAGTACGCGCCCTGTAGCGGCGCATTAAGCGCGGCGGGTGTGGTGGTTACGCGCAGCGTGACCGCTACACTTGCCAGCGCCCTAGCGCCCGCTCCTTTCGCTTTCTTCCCCTCCTTTGTCGCCACGTTCGCCGGCTTTCCCCGTCAAGCTCTAAATCGGGGGCTCCCTTTAGGGTTCCGATTTAGTGCTTTACGGCACCTCGACCCCAAAAAACTTGATTAGGGTGATGGTTCACGTAGTGGGCCATCGCCCTGATAGACGGTTTTTCGCCCTTTGACGTTGGAGTCCACGTTCTTTAATAGTGGACTCTTGTTCCAAACTGGAACAACACTCAACCCTATCTCGGTCTATTCTTTTGATTTATAAGGGATTTTGCCGATTTCGGCCTATTGGTTAAAAAATGAGCTGATTTAACAAAAATTTAACGCGAATTTTAACAAAATATTAACGTTTACAATTTAAATATTTGCTTATACAATCTTCCTGTTTTTGGGGCTTTTCTGATTATCAACCGGGGTACATATGATTGACATGCTAGTTTTACGATTACCGTTCATCGATTCTCTTGTTTGCTCCAGACTCTCAGGCAATGACCTGATAGCCTTTGTAGACCTCTCAAAAATAGCTACCCTCTCCGGCATGAATTTATCAGCTAGAACGGTTGAATATCATATTGATGGTGATTTGACTGTCTCCGGCCTTTCTCACCCGTTTGAATCTTTACCTACACATTACTCAGGCATTGCATTTAAAATATATGAGGGTTCTAAAAATTTTTATCCTTGCGTTGAAATAAAGGATTCTCCCGCAAAAGTATTACAGGGTCATAATGTTTTTGGTACAACCGATTTAGCTTTATGCTCTGAGGCTTTATTGCTTAATTTTGCTAATTCTTTGCCTTGCCTGTATGATTTATTGGATGTTGGAANTTCCATTCGCCCTTCAGGCTGCGCAACTGTTGGGAAGGGCGATCGGTGCGGGCCTCTTCGCTATTACGCCAGCTGGCGAAAGGGGGATGTGCTGCAAGGCGATTAAGTTGGGTAACGCCAGGGTTTTCCCAGTCACGACGTTGTAAAACGACGGCCAGTGCC

pTD3547

Contents in bold: Modified MCR in pRS316

Genetic markers: *URA3*, *CEN6/ARS4*, Amp^R^

Construction: *Sa*lI, *Xho*I and *Kpn*I deleted from the MCR of pRS316 (ATCC 77145) by standard techniques.

TCGCGCGTTTCGGTGATGACGGTGAAAACCTCTGACACATGCAGCTCCCGGAGACGGTCACAGCTTGTCTGTAAGCGGATGCCGGGAGCAGACAAGCCCGTCAGGGCGCGTCAGCGGGTGTTGGCGGGTGTCGGGGCTGGCTTAACTATGCGGCATCAGAGCAGATTGTACTGAGAGTGCACCACGCTTTTCAATTCAATTCATCATTTTTTTTTTATTCTTTTTTTTGATTTCGGTTTCTTTGAAATTTTTTTGATTCGGTAATCTCCGAACAGAAGGAAGAACGAAGGAAGGAGCACAGACTTAGATTGGTATATATACGCATATGTAGTGTTGAAGAAACATGAAATTGCCCAGTATTCTTAACCCAACTGCACAGAACAAAAACCTGCAGGAAACGAAGATAAATCATGTCGAAAGCTACATATAAGGAACGTGCTGCTACTCATCCTAGTCCTGTTGCTGCCAAGCTATTTAATATCATGCACGAAAAGCAAACAAACTTGTGTGCTTCATTGGATGTTCGTACCACCAAGGAATTACTGGAGTTAGTTGAAGCATTAGGTCCCAAAATTTGTTTACTAAAAACACATGTGGATATCTTGACTGATTTTTCCATGGAGGGCACAGTTAAGCCGCTAAAGGCATTATCCGCCAAGTACAATTTTTTACTCTTCGAAGACAGAAAATTTGCTGACATTGGTAATACAGTCAAATTGCAGTACTCTGCGGGTGTATACAGAATAGCAGAATGGGCAGACATTACGAATGCACACGGTGTGGTGGGCCCAGGTATTGTTAGCGGTTTGAAGCAGGCGGCAGAAGAAGTAACAAAGGAACCTAGAGGCCTTTTGATGTTAGCAGAATTGTCATGCAAGGGCTCCCTATCTACTGGAGAATATACTAAGGGTACTGTTGACATTGCGAAGAGCGACAAAGATTTTGTTATCGGCTTTATTGCTCAAAGAGACATGGGTGGAAGAGATGAAGGTTACGATTGGTTGATTATGACACCCGGTGTGGGTTTAGATGACAAGGGAGACGCATTGGGTCAACAGTATAGAACCGTGGATGATGTGGTCTCTACAGGATCTGACATTATTATTGTTGGAAGAGGACTATTTGCAAAGGGAAGGGATGCTAAGGTAGAGGGTGAACGTTACAGAAAAGCAGGCTGGGAAGCATATTTGAGAAGATGCGGCCAGCAAAACTAAAAAACTGTATTATAAGTAAATGCATGTATACTAAACTCACAAATTAGAGCTTCAATTTAATTATATCAGTTATTACCCTGCGGTGTGAAATACCGCACAGATGCGTAAGGAGAAAATACCGCATCAGGAAATTGTAAACGTTAATATTTTGTTAAAATTCGCGTTAAATTTTTGTTAAATCAGCTCATTTTTTAACCAATAGGCCGAAATCGGCAAAATCCCTTATAAATCAAAAGAATAGACCGAGATAGGGTTGAGTGTTGTTCCAGTTTGGAACAAGAGTCCACTATTAAAGAACGTGGACTCCAACGTCAAAGGGCGAAAAACCGTCTATCAGGGCGATGGCCCACTACGTGAACCATCACCCTAATCAAGTTTTTTGGGGTCGAGGTGCCGTAAAGCACTAAATCGGAACCCTAAAGGGAGCCCCCGATTTAGAGCTTGACGGGGAAAGCCGGCGAACGTGGCGAGAAAGGAAGGGAAGAAAGCGAAAGGAGCGGGCGCTAGGGCGCTGGCAAGTGTAGCGGTCACGCTGCGCGTAACCACCACACCCGCCGCGCTTAATGCGCCGCTACAGGGCGCGTCGCGCCATTCGCCATTCAGGCTGCGCAACTGTTGGGAAGGGCGATCGGTGCGGGCCTCTTCGCTATTACGCCAGCTGGCGAAGGGGGGATGTGCTGCAAGGCGATTAAGTTGGGTAACGCCAGGGTTTTCCCAGTCACGACGTTGTAAAACGACGGCCAGTGAATTGTAATACGACTCACTATAGGGCGAATTGGAGCTCCACC**GCGGTGGCGGCCGCTCTAGAACTAGTGGATCCCCCGGGCTGCAGGAATTCGATATCAAGCTTATCGATACCGTCTCGA**CAGCTTTTGTTCCCTTTAGTGAGGGTTAATTTCGAGCTTGGCGTAATCATGGTCATAGCTGTTTCCTGTGTGAAATTGTTATCCGCTCACAATTCCACACAACATACGAGCCGGAAGCATAAAGTGTAAAGCCTGGGGTGCCTAATGAGTGAGCTAACTCACATTAATTGCGTTGCGCTCACTGCCCGCTTTCCAGTCGGGAAACCTGTCGTGCCAGCTGCATTAATGAATCGGCCAACGCGCGGGGAGAGGCGGTTTGCGTATTGGGCGCTCTTCCGCTTCCTCGCTCACTGACTCGCTGCGCTCGGTCGTTCGGCTGCGGCGAGCGGTATCAGCTCACTCAAAGGCGGTAATACGGTTATCCACAGAATCAGGGGATAACGCAGGAAAGAACATGTGAGCAAAAGGCCAGCAAAAGGCCAGGAACCGTAAAAAGGCCGCGTTGCTGGCGTTTTTCCATAGGCTCGGCCCCCCTGACGAGCATCACAAAAATCGACGCTCAAGTCAGAGGTGGCGAAACCCGACAGGACTATAAAGATACCAGGCGTTCCCCCCTGGAAGCTCCCTCGTGCGCTCTCCTGTTCCGACCCTGCCGCTTACCGGATACCTGTCCGCCTTTCTCCCTTCGGGAAGCGTGGCGCTTTCTCAATGCTCACGCTGTAGGTATCTCAGTTCGGTGTAGGTCGTTCGCTCCAAGCTGGGCTGTGTGCACGAACCCCCCGTTCAGCCCGACCGCTGCGCCTTATCCGGTAACTATCGTCTTGAGTCCAACCCGGTAAGACACGACTTATCGCCACTGGCAGCAGCCACTGGTAACAGGATTAGCAGAGCGAGGTATGTAGGCGGTGCTACAGAGTTCTTGAAGTGGTGGCCTAACTACGGCTACACTAGAAGGACAGTATTTGGTATCTGCGCTCTGCTGAAGCCAGTTACCTTCGGAAAAAGAGTTGGTAGCTCTTGATCCGGCAAACAAACCACCGCTGGTAGCGGTGGTTTTTTTGTTTGCAAGCAGCAGATTACGCGCAGAAAAAAAGGATCTCAAGAAGATCCTTTGATCTTTTCTACGGGGTCTGACGCTCAGTGGAACGAAAACTCACGTTAAGGGATTTTGGTCATGAGATTATCAAAAAGGATCTTCACCTAGATCCTTTTAAATTAAAAATGAAGTTTTAAATCAATCTAAAGTATATATGAGTAAACTTGGTCTGACAGTTACCAATGCTTAATCAGTGAGGCACCTATCTCAGCGATCTGTCTATTTCGTTCATCCATAGTTGCCTGACTGCCCGTCGTGTAGATAACTACGATACGGGAGGGCTTACCATCTGGCCCCAGTGCTGCAATGATACCGCGAGACCCACGCTCACCGGCTCCAGATTTATCAGCAATAAACCAGCCAGCCGGAAGGGCCGAGCGCAGAAGTGGTCCTGCAACTTTATCCGCCTCCATCCAGTCTATTAATTGTTGCCGGGAAGCTAGAGTAAGTAGTTCGCCAGTTAATAGTTTGCGCAACGTTGTTGCCATTGCTACAGGCATCGTGGTGTCACGCTCGTCGTTTGGTATGGCTTCATTCAGCTCCGGTTCCCAACGATCAAGGCGAGTTACATGATCCCCCATGTTGTGAAAAAAAGCGGTTAGCTCCTTCGGTCCTCCGATCGTTGTCAGAAGTAAGTTGGCCGCAGTGTTATCACTCATGGTTATGGCAGCACTGCATAATTCTCTTACTGTCATGCCATCCGTAAGATGCTTTTCTGTGACTGGTGAGTACTCAACCAAGTCATTCTGAGAATAGTGTATGCGGCGACCGAGTTGCTCTTGCCCGGCGTCAATACGGGATAATACCGCGCCACATAGCAGAACTTTAAAAGTGCTCATCATTGGAAAACGTTCTTCGGGGCGAAAACTCTCAAGGATCTTACCGCTGTTGAGATCCAGTTCGATGTAACCCACTCGTGCACCCAACTGATCTTCAGCATCTTTTACTTTCACCAGCGTTTCTGGGTGAGCAAAAACAGGAAGGCAAAATGCCGCAAAAAAGGGAATAAGGGCGACACGGAAATGTTGAATACTCATACTCTTCCTTTTTCAATATTATTGAAGCATTTATCAGGGTTATTGTCTCATGAGCGGATACATATTTGAATGTATTTAGAAAAATAAACAAATAGGGGTTCCGCGCACATTTCCCCGAAAAGTGCCACCTGGGTCCTTTTCATCACGTGCTATAAAAATAATTATAATTTAAATTTTTTAATATAAATATATAAATTAAAAATAGAAAGTAAAAAAAGAAATTAAAGAAAAAATAGTTTTTGTTTTCCGAAGATGTAAAAGACTCTAGGGGGATCGCCAACAAATACTACCTTTTATCTTGCTCTTCCTGCTCTCAGGTATTAATGCCGAATTGTTTCATCTTGTCTGTGTAGAAGACCACACACGAAAATCCTGTGATTTTACATTTTACTTATCGTTAATCGAATGTATATCTATTTAATCTGCTTTTCTTGTCTAATAAATATATATGTAAAGTACGCTTTTTGTTGAAATTTTTTAAACCTTTGTTTATTTTTTTTTCTTCATTCCGTAACTCTTCTACCTTCTTTATTTACTTTCTAAAATCCAAATACAAAACATAAAAATAAATAAACACAGAGTAAATTCCCAAATTATTCCATCATTAAAAGATACGAGGCGCGTGTAAGTTACAGGCAAGCGATCCGTCCTAAGAAACCATTATTATCATGACATTAACCTATAAAAATAGGCGTATCACGAGGCCCTTTCGTC

pTD3548

Contents in bold: Ty3-IN-GFP (GFP in green)

Genetic markers: *URA3*, *CEN6/ARS4*, Amp^R^

Construction: The *Hind*III-*Not*I fragment encoding Ty3-IN-GFP fusion from pNB2176 (Beliakova-Bethell N, Beckham C, Giddings TH, Jr., Winey M, Parker R, et al. Virus-like particles of the Ty3 retrotransposon assemble in association with P-body components. RNA. 2006; 12: 94-101) was inserted into *Hind*III and *Not*I-digested pTD3547.

GACGAAAGGGCCTCGTGATACGCCTATTTTTATAGGTTAATGTCATGATAATAATGGTTTCTTAGGACGGATCGCTTGCCTGTAACTTACACGCGCCTCGTATCTTTTAATGATGGAATAATTTGGGAATTTACTCTGTGTTTATTTATTTTTATGTTTTGTATTTGGATTTTAGAAAGTAAATAAAGAAGGTAGAAGAGTTACGGAATGAAGAAAAAAAAATAAACAAAGGTTTAAAAAATTTCAACAAAAAGCGTACTTTACATATATATTTATTAGACAAGAAAAGCAGATTAAATAGATATACATTCGATTAACGATAAGTAAAATGTAAAATCACAGGATTTTCGTGTGTGGTCTTCTACACAGACAAGATGAAACAATTCGGCATTAATACCTGAGAGCAGGAAGAGCAAGATAAAAGGTAGTATTTGTTGGCGATCCCCCTAGAGTCTTTTACATCTTCGGAAAACAAAAACTATTTTTTCTTTAATTTCTTTTTTTACTTTCTATTTTTAATTTATATATTTATATTAAAAAATTTAAATTATAATTATTTTTATAGCACGTGATGAAAAGGACCCAGGTGGCACTTTTCGGGGAAATGTGCGCGGAACCCCTATTTGTTTATTTTTCTAAATACATTCAAATATGTATCCGCTCATGAGACAATAACCCTGATAAATGCTTCAATAATATTGAAAAAGGAAGAGTATGAGTATTCAACATTTCCGTGTCGCCCTTATTCCCTTTTTTGCGGCATTTTGCCTTCCTGTTTTTGCTCACCCAGAAACGCTGGTGAAAGTAAAAGATGCTGAAGATCAGTTGGGTGCACGAGTGGGTTACATCGAACTGGATCTCAACAGCGGTAAGATCCTTGAGAGTTTTCGCCCCGAAGAACGTTTTCCAATGATGAGCACTTTTAAAGTTCTGCTATGTGGCGCGGTATTATCCCGTATTGACGCCGGGCAAGAGCAACTCGGTCGCCGCATACACTATTCTCAGAATGACTTGGTTGAGTACTCACCAGTCACAGAAAAGCATCTTACGGATGGCATGACAGTAAGAGAATTATGCAGTGCTGCCATAACCATGAGTGATAACACTGCGGCCAACTTACTTCTGACAACGATCGGAGGACCGAAGGAGCTAACCGCTTTTTTTCACAACATGGGGGATCATGTAACTCGCCTTGATCGTTGGGAACCGGAGCTGAATGAAGCCATACCAAACGACGAGCGTGACACCACGATGCCTGTAGCAATGGCAACAACGTTGCGCAAACTATTAACTGGCGAACTACTTACTCTAGCTTCCCGGCAACAATTAATAGACTGGATGGAGGCGGATAAAGTTGCAGGACCACTTCTGCGCTCGGCCCTTCCGGCTGGCTGGTTTATTGCTGATAAATCTGGAGCCGGTGAGCGTGGGTCTCGCGGTATCATTGCAGCACTGGGGCCAGATGGTAAGCCCTCCCGTATCGTAGTTATCTACACGACGGGCAGTCAGGCAACTATGGATGAACGAAATAGACAGATCGCTGAGATAGGTGCCTCACTGATTAAGCATTGGTAACTGTCAGACCAAGTTTACTCATATATACTTTAGATTGATTTAAAACTTCATTTTTAATTTAAAAGGATCTAGGTGAAGATCCTTTTTGATAATCTCATGACCAAAATCCCTTAACGTGAGTTTTCGTTCCACTGAGCGTCAGACCCCGTAGAAAAGATCAAAGGATCTTCTTGAGATCCTTTTTTTCTGCGCGTAATCTGCTGCTTGCAAACAAAAAAACCACCGCTACCAGCGGTGGTTTGTTTGCCGGATCAAGAGCTACCAACTCTTTTTCCGAAGGTAACTGGCTTCAGCAGAGCGCAGATACCAAATACTGTCCTTCTAGTGTAGCCGTAGTTAGGCCACCACTTCAAGAACTCTGTAGCACCGCCTACATACCTCGCTCTGCTAATCCTGTTACCAGTGGCTGCTGCCAGTGGCGATAAGTCGTGTCTTACCGGGTTGGACTCAAGACGATAGTTACCGGATAAGGCGCAGCGGTCGGGCTGAACGGGGGGTTCGTGCACACAGCCCAGCTTGGAGCGAACGACCTACACCGAACTGAGATACCTACAGCGTGAGCATTGAGAAAGCGCCACGCTTCCCGAAGGGAGAAAGGCGGACAGGTATCCGGTAAGCGGCAGGGTCGGAACAGGAGAGCGCACGAGGGAGCTTCCAGGGGGGAACGCCTGGTATCTTTATAGTCCTGTCGGGTTTCGCCACCTCTGACTTGAGCGTCGATTTTTGTGATGCTCGTCAGGGGGGCCGAGCCTATGGAAAAACGCCAGCAACGCGGCCTTTTTACGGTTCCTGGCCTTTTGCTGGCCTTTTGCTCACATGTTCTTTCCTGCGTTATCCCCTGATTCTGTGGATAACCGTATTACCGCCTTTGAGTGAGCTGATACCGCTCGCCGCAGCCGAACGACCGAGCGCAGCGAGTCAGTGAGCGAGGAAGCGGAAGAGCGCCCAATACGCAAACCGCCTCTCCCCGCGCGTTGGCCGATTCATTAATGCAGCTGGCACGACAGGTTTCCCGACTGGAAAGCGGGCAGTGAGCGCAACGCAATTAATGTGAGTTAGCTCACTCATTAGGCACCCCAGGCTTTACACTTTATGCTTCCGCTATGTTGTGTGGAATTGTGAGCGGATAACAATTTCACACAGGAAACAGCTATGACCATGATTACGCCAAGCTCGAAATTAACCCTCACTAAAGGGAACAAAAGCTGTCGAGACGGTATCGATAAGCTT**TGTTGTATCTCAAAATGAGATATGTCAGTATGACAATACGTCACCCTGAACGTTCATAAAACACATATGAAACAACCTTATAACAAAACGAACAACATGAGACAAAACCCGACCTTCCCTAGCTGAACTACCCAAAGTATAAATGCCTGAACAATTAGTTTAGATCCGAGATTCCGCGCTTCCACCACTTAGTATGATTCATATTTTATATAATATATAAGATAAGTAACATTCCGTGAATTAATCTGATAAACTGTTTTGACAACTGGTTACTTCCCTAAGACTGTTTATATTAGGATTGTCAAGACACTCCGGTATTACTCGAGCCCGTAATACAACACCTGGTAGCGTTAAAGGTTACTAATTGTTCAAACGAACCATCGAAAAGCCGAACCTAGCTACACCACACCCCAGTATGAGCTTTATGGATCAAATCCCAGGAGGAGGAAATTATCCAAAACTCCCAGTAGAATGCCTTCCTAACTTCCCGATCCAACCATCTTTGACCTTCAGAGGTAGAAATGACTCGCATAAACTGAAAAACTTTATCTCCGAAATAATGTTAAACATGTCTATGATATCTTGGCCGAATGATGCCAGTCGTATTGTGTACTGCAGAAGACATTTATTAAACCCCGCTGCTCAGTGGGCTAATGACTTTGTACAAGAACAAGGTATACTTGAAATAACATTCGACACATTCATACAAGGATTATATCAGCATTTCTATAAGCCACCAGATATCAATAAAATCTTTAATGCAATCACGCAACTTTCCGAAGCTAAACTTGGTATTGAGCGTCTCAACCAACGATTCAGAAAGATTTGGGACAGAATGCCACCAGACTTCATGACCGAAAAAGCTGCCATAATGACATATACTAGGCTATTGACAAAGGAAACCTATAATATTGTCAGAATGCACAAACCAGAGACATTAAAAGACGCCATGGAAGAGGCTTACCAGACAACTGCACTAACTGAAAGATTCTTCCCAGGATTCGAACTTGATGCTGATGGAGACACTATCATCGGTGCCACAACCCACTTACAAGAAGAATACGACTCTGACTATGATTCAGAAGATAATCTGACCCAGAATGGATACGTCCATACCGTAAGGACAAGAAGATCTTACAATAAACCAATGTCAAATCATCGAAACAGGAGAAATAACAACCCATCTAGAGAAGAATGTATAAAAAATCGGCTATGCTTCTATTGTAAGAAAGAGGGACATCGCCTGAACGAATGTAGAGCACGTAAGGCGAGTTCTAACCGATCTTGAACTCGAATCAAAAGACCAACAAACTCCTTTTATCAAAACCTTACCAATTGTACACTATATCGCCATCCCCGAGATGGACAATACCGCCGAAAAAACCATAAAAATACAAAACACGAAAGTAAAAACCCTGTTTGACAGTGGATCACCCACGTCATTTATCCGAAGAGATATTGTAGAACTTCTCAAATACGAAATCTACGAGACCCCTCCACTCCGTTTTAGAGGATTCGTAGCCACCAAATCCGCCGTTACATCCGAAGCAGTCACCATTGACCTCAAAATCAATGACCTGCATATAACTTTAGCCGCGTACATACTGGATAACATGGACTACCAATTGTTAATTGGAAATCCAATCTTACGCCGCTACCCGAAAATCCTGCACACAGTACTGAATACCAGAGAGAGCCCCGACTCCTTAAAGCCCAAGACTTATCGCTCCGAAACCGTTAATAACGTTAGAACCTACTCCGCTGGTAATCGTGGTAACCCCAGAAACATAAAACTGTCTTTTGCCCCCACCATTCTCGAAGCAACTGACCCGAAATCCGCTGGTAATCGTGGTGACTCCAGAACCAAAACCCTGTCTCTTGCAACCACTACTCCTGCAGCAATTGACCCGCTTACGACCCTTGATAACCCAGGTAGTACTCAAAGTACATTTGCGCAATTCCCGATACCTGAAGAAGCGAGCATCCTAGAAGAGGATGGAAAATACTCCAACGTTGTCTCAACCATTCAGAGTGTAGAACCTAATGCTACTGATCACAGCAATAAGGACACCTTTTGCACTTTGCCAGTTTGGTTACAACAGAAGTATAGAGAGATCATACGTAATGATCTCCCACCAAGACCTGCCGACATTAATAACATCCCCGTAAAACATGATATTGAAATTAAACCTGGCGCAAGACTACCTCGACTACAGCCATACCATGTTACAGAAAAGAACGAACAAGAAATCAACAAAATAGTTCAAAAACTGCTCGATAACAAGTTCATTGTTCCCTCAAAGTCGCCTTGCAGCTCCCCTGTAGTCCTCGTCCCGAAGAAAGACGGTACCTTCCGACTCTGCGTCGATTACCGCACCCTGAACAAAGCTACCATCTCCGACCCATTCCCATTACCCAGAATCGACAACCTATTGAGCCGTATTGGAAATGCCCAGATATTTACCACGCTAGATTTGCATAGTGGTTACCACCAGATCCCGATGGAACCCAAAGACCGCTACAAAACCGCCTTTGTCACACCATCCGGTAAGTATGAATATACCGTCATGCCATTTGGCTTAGTCAATGCACCTAGTACATTCGCAAGATACATGGCTGATACATTTAGAGACCTGAGATTCGTCAATGTTTACCTTGATGATATATTAATATTCTCCGAATCTCCAGAAGAACATTGGAAACATTTAGACACGGTACTAGAAAGATTAAAGAACGAGAACCTCATTGTTAAGAAGAAAAAATGTAAATTTGCATCTGAAGAAACTGAGTTTTTAGGCTATAGTATTGGAATCCAGAAAATAGCTCCACTACAGCACAAATGTGCAGCAATCCGAGACTTTCCGACGCCTAAAACAGTAAAACAAGCACAGAGATTTTTAGGAATGATTAATTACTACAGACGATTCATTCCAAATTGCTCCAAGATTGCACAGCCAATCCAACTGTTTATTTGTGACAAAAGTCAATGGACAGAAAAACAAGACAAGGCAATTGATAAACTAAAAGACGCCTTGTGTAACTCCCCCGTCCTAGTACCATTCAACAACAAAGCAAACTACCGACTTACAACAGACGCCTCAAAAGACGGCATTGGTGCTGTTCTAGAAGAAGTCGACAACAAGAACAAACTTGTTGGTGTCGTCGGTTACTTCTCTAAATCCTTAGAGAGTGCCCAGAAAAACTATCCTGCTGGCGAATTAGAACTACTTGGAATTATCAAAGCACTCCACCACTTCCGATATATGCTTCACGGAAAGCATTTCACGTTAAGAACAGACCACATTAGTTTGTTATCATTACAAAACAAGAACGAACCCGACGAACGCGTGCAACGCTGGTTAGATGACCTAGCCACATATGACTTCACCTTAGAATACCTAGCTGGACCCAAGAACGTTGTCGCAGATGCCATATCCCGTGCCGTATATACTATAACCCCCGAAACATCCCGACCTATCGACACAGAAAGCTGGAAATCTTACTACAAATCAGACCCATTATGTAGTGCTGTCTTAATTCATATGAAAGAATTGACACAACACAACGTCACACCTGAAGATATGTCAGCCTTCCGTAGTTACCAGAAGAAACTCGAACTATCAGAGACCTTCCGAAAGAATTATTCCCTAGAAGACGAAATGATCTATTACCAAGACCGACTAGTAGTACCAATAAAACAACAGAACGCAGTTATGAGACTATATCATGACCATACCTTATTTGGAGGACATTTTGGTGTAACAGTGACCCTTGCGAAAATCAGCCCAATTTACTATTGGCCAAAATTACAACATTCGATCATACAATACATCAGGACCTGCGTACAATGTCAACTAATAAAATCACACCGACCACGCTTACATGGACTATTACAACCACTCCCTATAGCAGAAGGAAGATGGCTTGATATATCAATGGATTTTGTGACAGGATTACCCCCGACATCAAATAACTTGAATATGATCCTCGTCGTAGTTGATCGTTTTTCGAAACGCGCTCACTTCATAGCTACAAGGAAAACCTTAGACGCAACACAACTAATAGATCTACTCTTTCGATACATTTTTTCATATCATGGTTTTCCCAGGACAATAACCAGTGATAGAGATGTCCGTATGACCGCCGACAAATATCAAGAACTCACGAAAAGACTAGGAATAAAATCGACAATGTCTTCCGCGAACCACCCCCAAACAGATGGACAATCCGAACGAACGATACAGACATTAAACAGGTTACTAAGAGCCTATGCTTCAACCAATATTCAGAATTGGCATGTATATTTACCACAAATCGAATTTGTTTACAATTCTACACCTACTAGAACACTTGGAAAATCACCATTTGAAATTGATTTAGGATATTTACCGAATACCCCTGCTATTAAGTCAGATGACGAAGTCAACGCAAGAAGTTTTACTGCCGTAGAACTTGCCAAACACCTCAAAGCCCTTACCATCCAAACGAAGGAACAGCTAGAACACGCTCAAATCGAAATGGAAACTAATAACAATCAAAGACGTAAACCCTTATTGTTAAACATAGGAGATCACGTATTAGTGCATAGAGATGCATACTTCAAGAAAGGTGCTTATATGAAAGTACAACAAATATACGTCGGACCATTTCGAGTTGTCAAGAAAATAAACGATAACGCCTACGAACTAGATTTAAACTCTCACAAGAAAAAGCACAGAGTTATTAATGTACAATTCCTGAAAAAGTTTGTATACCGTCCAGACGCGTACCCAAAGAATAAACCAATCAGCTCCACTGAAAGAATTAAGAGAGCACACGAAGTTACTGCACTCATAGGAATAGATACTACACACAAAACTTACTTATGTCACATGCAAGATGTAGACCCAACACTTTCAGTAGAATACTCAGAAGCTGAATTTTGCCAAATTCCCGAAAGAACACGAAGATCAATATTAGCCAACTTTAGACAACTCTACGAAACACAAGACAACCCTGAGAGAGAGGAAGATGTTGTATCTCAAAATGAGATATGTCAGTATGACAATACGTCACCCGGGATGAGTAAAGGAGAAGAACTTTTCACTGGAGTTGTCCCAATTCTTGTTGAATTAGATGGTGATGTTAATGGGCACAAATTTTCTGTCAGTGGAGAGGGTGAAGGTGATGCAACATACGGAAAACTTACCCTTAAATTTATTTGCACTACTGGAAAACTACCTGTTCCATGGCCAACACTTGTCACTACTTTCACTTATGGTGTTCAATGCTTTTCAAGATACCCAGATCATATGAAACAGCATGACTTTTTCAAGAGTGCCATGCCCGAAGGTTATGTACAGGAAAGAACTATATTTTTCAAAGATGACGGGAACTACAAGACACGTGCTGAAGTCAAGTTTGAAGGTGATACCCTTGTTAATAGAATCGAGTTAAAAGGTATTGATTTTAAAGAAGATGGAAACATTCTTGGACACAAATTGGAATACAACTATAACTCACACAATGTATACATCATGGCAGACAAACAAAAGAATGGAATCAAAGTTAACTTCAAAATTAGACACAACATTGAAGATGGAAGCGTTCAACTAGCAGACCATTATCAACAAAATACTCCAATTGGCGATGGCCCTGTCCTTTTACCAGACAACCATTACCTGTCCACACAATCTGCCCTTTCGAAAGATCCCAACGAAAAGAGAGACCACATGGTCCTTCTTGAGTTTGTAACAGCTGCTGGGATTACACATGGCATGGATGAACTATACAAATAATAACCCGGGACGTTCATAAAACACATATGAAACAACCTTATAACAAAACGAACAACATGAGACAAAACCCGACCTTCCCTAGCTGAACTACCCAAAGTATAAATGCCTGAACAATTAGTTTAGATCCGAGATTCCGCGCTTCCACCACTTAGTATGATTCATATTTTATATAATATATAAGATAAGTAACATTCCGTGAATTAATCTGATAAACTGTTTTGACAACTGGTTACTTCCCTAAGACTGTTTATATTAGGATTGTCAAGACACTCCGGTATTACTCGAGCCCGTAATACAACA**GCGGCCGCCACCGCGGTGGAGCTCCAATTCGCCCTATAGTGAGTCGTATTACAATTCACTGGCCGTCGTTTTACAACGTCGTGACTGGGAAAACCCTGGCGTTACCCAACTTAATCGCCTTGCAGCACATCCCCCCTTCGCCAGCTGGCGTAATAGCGAAGAGGCCCGCACCGATCGCCCTTCCCAACAGTTGCGCAGCCTGAATGGCGAATGGCGCGACGCGCCCTGTAGCGGCGCATTAAGCGCGGCGGGTGTGGTGGTTACGCGCAGCGTGACCGCTACACTTGCCAGCGCCCTAGCGCCCGCTCCTTTCGCTTTCTTCCCTTCCTTTCTCGCCACGTTCGCCGGCTTTCCCCGTCAAGCTCTAAATCGGGGGCTCCCTTTAGGGTTCCGATTTAGTGCTTTACGGCACCTCGACCCCAAAAAACTTGATTAGGGTGATGGTTCACGTAGTGGGCCATCGCCCTGATAGACGGTTTTTCGCCCTTTGACGTTGGAGTCCACGTTCTTTAATAGTGGACTCTTGTTCCAAACTGGAACAACACTCAACCCTATCTCGGTCTATTCTTTTGATTTATAAGGGATTTTGCCGATTTCGGCCTATTGGTTAAAAAATGAGCTGATTTAACAAAAATTTAACGCGAATTTTAACAAAATATTAACGTTTACAATTTCCTGATGCGGTATTTTCTCCTTACGCATCTGTGCGGTATTTCACACCGCAGGGTAATAACTGATATAATTAAATTGAAGCTCTAATTTGTGAGTTTAGTATACATGCATTTACTTATAATACAGTTTTTTAGTTTTGCTGGCCGCATCTTCTCAAATATGCTTCCCAGCCTGCTTTTCTGTAACGTTCACCCTCTACCTTAGCATCCCTTCCCTTTGCAAATAGTCCTCTTCCAACAATAATAATGTCAGATCCTGTAGAGACCACATCATCCACGGTTCTATACTGTTGACCCAATGCGTCTCCCTTGTCATCTAAACCCACACCGGGTGTCATAATCAACCAATCGTAACCTTCATCTCTTCCACCCATGTCTCTTTGAGCAATAAAGCCGATAACAAAATCTTTGTCGCTCTTCGCAATGTCAACAGTACCCTTAGTATATTCTCCAGTAGATAGGGAGCCCTTGCATGACAATTCTGCTAACATCAAAAGGCCTCTAGGTTCCTTTGTTACTTCTTCTGCCGCCTGCTTCAAACCGCTAACAATACCTGGGCCCACCACACCGTGTGCATTCGTAATGTCTGCCCATTCTGCTATTCTGTATACACCCGCAGAGTACTGCAATTTGACTGTATTACCAATGTCAGCAAATTTTCTGTCTTCGAAGAGTAAAAAATTGTACTTGGCGGATAATGCCTTTAGCGGCTTAACTGTGCCCTCCATGGAAAAATCAGTCAAGATATCCACATGTGTTTTTAGTAAACAAATTTTGGGACCTAATGCTTCAACTAACTCCAGTAATTCCTTGGTGGTACGAACATCCAATGAAGCACACAAGTTTGTTTGCTTTTCGTGCATGATATTAAATAGCTTGGCAGCAACAGGACTAGGATGAGTAGCAGCACGTTCCTTATATGTAGCTTTCGACATGATTTATCTTCGTTTCCTGCAGGTTTTTGTTCTGTGCAGTTGGGTTAAGAATACTGGGCAATTTCATGTTTCTTCAACACTACATATGCGTATATATACCAATCTAAGTCTGTGCTCCTTCCTTCGTTCTTCCTTCTGTTCGGAGATTACCGAATCAAAAAAATTTCAAAGAAACCGAAATCAAAAAAAAGAATAAAAAAAAAATGATGAATTGAATTGAAAAGCGTGGTGCACTCTCAGTACAATCTGCTCTGATGCCGCATAGTTAAGCCAGCCCCGACACCCGCCAACACCCGCTGACGCGCCCTGACGGGCTTGTCTGCTCCCGGCATCCGCTTACAGACAAGCTGTGACCGTCTCCGGGAGCTGCATGTGTCAGAGGTTTTCACCGTCATCACCGAAACGCGCGA

pTD3655

Contents in bold: Ty3-IN-mCherry (mCherry in red)

Genetic markers: *URA3*, *CEN6/ARS4*, Amp^R^

Construction: GFP in pTD3548 was replaced with mCherry from pBS34 (Yeast Resource Center, University of Washington, Seattle, WA). DNA encoding mCherry was amplified from pBS34 using primers TD3459 (F5-GTCAGTATGACAATACGTCACCCGGGATGGTGAGCAAGGGCGA-3) and TD3460 (F5-CATATGTGTTTTATGAACGTCCCGGGCTACTTGTACAGCTCGTC-3) that introduced flanking *Xma*1 restriction sites (underlined). The PCR product was digested with *Xma*I and inserted into *Xma*I- digested pTD3548.

GACGAAAGGGCCTCGTGATACGCCTATTTTTATAGGTTAATGTCATGATAATAATGGTTTCTTAGGACGGATCGCTTGCCTGTAACTTACACGCGCCTCGTATCTTTTAATGATGGAATAATTTGGGAATTTACTCTGTGTTTATTTATTTTTATGTTTTGTATTTGGATTTTAGAAAGTAAATAAAGAAGGTAGAAGAGTTACGGAATGAAGAAAAAAAAATAAACAAAGGTTTAAAAAATTTCAACAAAAAGCGTACTTTACATATATATTTATTAGACAAGAAAAGCAGATTAAATAGATATACATTCGATTAACGATAAGTAAAATGTAAAATCACAGGATTTTCGTGTGTGGTCTTCTACACAGACAAGATGAAACAATTCGGCATTAATACCTGAGAGCAGGAAGAGCAAGATAAAAGGTAGTATTTGTTGGCGATCCCCCTAGAGTCTTTTACATCTTCGGAAAACAAAAACTATTTTTTCTTTAATTTCTTTTTTTACTTTCTATTTTTAATTTATATATTTATATTAAAAAATTTAAATTATAATTATTTTTATAGCACGTGATGAAAAGGACCCAGGTGGCACTTTTCGGGGAAATGTGCGCGGAACCCCTATTTGTTTATTTTTCTAAATACATTCAAATATGTATCCGCTCATGAGACAATAACCCTGATAAATGCTTCAATAATATTGAAAAAGGAAGAGTATGAGTATTCAACATTTCCGTGTCGCCCTTATTCCCTTTTTTGCGGCATTTTGCCTTCCTGTTTTTGCTCACCCAGAAACGCTGGTGAAAGTAAAAGATGCTGAAGATCAGTTGGGTGCACGAGTGGGTTACATCGAACTGGATCTCAACAGCGGTAAGATCCTTGAGAGTTTTCGCCCCGAAGAACGTTTTCCAATGATGAGCACTTTTAAAGTTCTGCTATGTGGCGCGGTATTATCCCGTATTGACGCCGGGCAAGAGCAACTCGGTCGCCGCATACACTATTCTCAGAATGACTTGGTTGAGTACTCACCAGTCACAGAAAAGCATCTTACGGATGGCATGACAGTAAGAGAATTATGCAGTGCTGCCATAACCATGAGTGATAACACTGCGGCCAACTTACTTCTGACAACGATCGGAGGACCGAAGGAGCTAACCGCTTTTTTTCACAACATGGGGGATCATGTAACTCGCCTTGATCGTTGGGAACCGGAGCTGAATGAAGCCATACCAAACGACGAGCGTGACACCACGATGCCTGTAGCAATGGCAACAACGTTGCGCAAACTATTAACTGGCGAACTACTTACTCTAGCTTCCCGGCAACAATTAATAGACTGGATGGAGGCGGATAAAGTTGCAGGACCACTTCTGCGCTCGGCCCTTCCGGCTGGCTGGTTTATTGCTGATAAATCTGGAGCCGGTGAGCGTGGGTCTCGCGGTATCATTGCAGCACTGGGGCCAGATGGTAAGCCCTCCCGTATCGTAGTTATCTACACGACGGGCAGTCAGGCAACTATGGATGAACGAAATAGACAGATCGCTGAGATAGGTGCCTCACTGATTAAGCATTGGTAACTGTCAGACCAAGTTTACTCATATATACTTTAGATTGATTTAAAACTTCATTTTTAATTTAAAAGGATCTAGGTGAAGATCCTTTTTGATAATCTCATGACCAAAATCCCTTAACGTGAGTTTTCGTTCCACTGAGCGTCAGACCCCGTAGAAAAGATCAAAGGATCTTCTTGAGATCCTTTTTTTCTGCGCGTAATCTGCTGCTTGCAAACAAAAAAACCACCGCTACCAGCGGTGGTTTGTTTGCCGGATCAAGAGCTACCAACTCTTTTTCCGAAGGTAACTGGCTTCAGCAGAGCGCAGATACCAAATACTGTCCTTCTAGTGTAGCCGTAGTTAGGCCACCACTTCAAGAACTCTGTAGCACCGCCTACATACCTCGCTCTGCTAATCCTGTTACCAGTGGCTGCTGCCAGTGGCGATAAGTCGTGTCTTACCGGGTTGGACTCAAGACGATAGTTACCGGATAAGGCGCAGCGGTCGGGCTGAACGGGGGGTTCGTGCACACAGCCCAGCTTGGAGCGAACGACCTACACCGAACTGAGATACCTACAGCGTGAGCATTGAGAAAGCGCCACGCTTCCCGAAGGGAGAAAGGCGGACAGGTATCCGGTAAGCGGCAGGGTCGGAACAGGAGAGCGCACGAGGGAGCTTCCAGGGGGGAACGCCTGGTATCTTTATAGTCCTGTCGGGTTTCGCCACCTCTGACTTGAGCGTCGATTTTTGTGATGCTCGTCAGGGGGGCCGAGCCTATGGAAAAACGCCAGCAACGCGGCCTTTTTACGGTTCCTGGCCTTTTGCTGGCCTTTTGCTCACATGTTCTTTCCTGCGTTATCCCCTGATTCTGTGGATAACCGTATTACCGCCTTTGAGTGAGCTGATACCGCTCGCCGCAGCCGAACGACCGAGCGCAGCGAGTCAGTGAGCGAGGAAGCGGAAGAGCGCCCAATACGCAAACCGCCTCTCCCCGCGCGTTGGCCGATTCATTAATGCAGCTGGCACGACAGGTTTCCCGACTGGAAAGCGGGCAGTGAGCGCAACGCAATTAATGTGAGTTACCTCACTCATTAGGCACCCCAGGCTTTACACTTTATGCTTCCGGCTCCTATGTTGTGTGGAATTGTGAGCGGATAACAATTTCACACAGGAAACAGCTATGACCATGATTACGCCAAGCTCGGAATTAACCCTCACTAAAGGGAACAAAAGCTGTCGAGACGGTATCGATAAGCTT**TGTTGTATCTCAAAATGAGATATGTCAGTATGACAATACGTCACCCTGAACGTTCATAAAACACATATGAAACAACCTTATAACAAAACGAACAACATGAGACAAAACCCGACCTTCCCTAGCTGAACTACCCAAAGTATAAATGCCTGAACAATTAGTTTAGATCCGAGATTCCGCGCTTCCACCACTTAGTATGATTCATATTTTATATAATATATAAGATAAGTAACATTCCGTGAATTAATCTGATAAACTGTTTTGACAACTGGTTACTTCCCTAAGACTGTTTATATTAGGATTGTCAAGACACTCCGGTATTACTCGAGCCCGTAATACAACACCTGGTAGCGTTAAAGGTTACTAATTGTTCAAACGAACCATCGAAAAGCCGAACCTAGCTACACCACACCCCAGTATGAGCTTTATGGATCAAATCCCAGGAGGAGGAAATTATCCAAAACTCCCAGTAGAATGCCTTCCTAACTTCCCGATCCAACCATCTTTGACCTTCAGAGGTAGAAATGACTCGCATAAACTGAAAAACTTTATCTCCGAAATAATGTTAAACATGTCTATGATATCTTGGCCGAATGATGCCAGTCGTATTGTGTACTGCAGAAGACATTTATTAAACCCCGCTGCTCAGTGGGCTAATGACTTTGTACAAGAACAAGGTATACTTGAAATAACATTCGACACATTCATACAAGGATTATATCAGCATTTCTATAAGCCACCAGATATCAATAAAATCTTTAATGCAATCACGCAACTTTCCGAAGCTAAACTTGGTATTGAGCGTCTCAACCAACGATTCAGAAAGATTTGGGACAGAATGCCACCAGACTTCATGACCGAAAAAGCTGCCATAATGACATATACTAGGCTATTGACAAAGGAAACCTATAATATTGTCAGAATGCACAAACCAGAGACATTAAAAGACGCCATGGAAGAGGCTTACCAGACAACTGCACTAACTGAAAGATTCTTCCCAGGATTCGAACTTGATGCTGATGGAGACACTATCATCGGTGCCACAACCCACTTACAAGAAGAATACGACTCTGACTATGATTCAGAAGATAATCTGACCCAGAATGGATACGTCCATACCGTAAGGACAAGAAGATCTTACAATAAACCAATGTCAAATCATCGAAACAGGAGAAATAACAACCCATCTAGAGAAGAATGTATAAAAAATCGGCTATGCTTCTATTGTAAGAAAGAGGGACATCGCCTGAACGAATGTAGAGCACGTAAGGCGAGTTCTAACCGATCTTGAACTCGAATCAAAAGACCAACAAACTCCTTTTATCAAAACCTTACCAATTGTACACTATATCGCCATCCCCGAGATGGACAATACCGCCGAAAAAACCATAAAAATACAAAACACGAAAGTAAAAACCCTGTTTGACAGTGGATCACCCACGTCATTTATCCGAAGAGATATTGTAGAACTTCTCAAATACGAAATCTACGAGACCCCTCCACTCCGTTTTAGAGGATTCGTAGCCACCAAATCCGCCGTTACATCCGAAGCAGTCACCATTGACCTCAAAATCAATGACCTGCATATAACTTTAGCCGCGTACATACTGGATAACATGGACTACCAATTGTTAATTGGAAATCCAATCTTACGCCGCTACCCGAAAATCCTGCACACAGTACTGAATACCAGAGAGAGCCCCGACTCCTTAAAGCCCAAGACTTATCGCTCCGAAACCGTTAATAACGTTAGAACCTACTCCGCTGGTAATCGTGGTAACCCCAGAAACATAAAACTGTCTTTTGCCCCCACCATTCTCGAAGCAACTGACCCGAAATCCGCTGGTAATCGTGGTGACTCCAGAACCAAAACCCTGTCTCTTGCAACCACTACTCCTGCAGCAATTGACCCGCTTACGACCCTTGATAACCCAGGTAGTACTCAAAGTACATTTGCGCAATTCCCGATACCTGAAGAAGCGAGCATCCTAGAAGAGGATGGAAAATACTCCAACGTTGTCTCAACCATTCAGAGTGTAGAACCTAATGCTACTGATCACAGCAATAAGGACACCTTTTGCACTTTGCCAGTTTGGTTACAACAGAAGTATAGAGAGATCATACGTAATGATCTCCCACCAAGACCTGCCGACATTAATAACATCCCCGTAAAACATGATATTGAAATTAAACCTGGCGCAAGACTACCTCGACTACAGCCATACCATGTTACAGAAAAGAACGAACAAGAAATCAACAAAATAGTTCAAAAACTGCTCGATAACAAGTTCATTGTTCCCTCAAAGTCGCCTTGCAGCTCCCCTGTAGTCCTCGTCCCGAAGAAAGACGGTACCTTCCGACTCTGCGTCGATTACCGCACCCTGAACAAAGCTACCATCTCCGACCCATTCCCATTACCCAGAATCGACAACCTATTGAGCCGTATTGGAAATGCCCAGATATTTACCACGCTAGATTTGCATAGTGGTTACCACCAGATCCCGATGGAACCCAAAGACCGCTACAAAACCGCCTTTGTCACACCATCCGGTAAGTATGAATATACCGTCATGCCATTTGGCTTAGTCAATGCACCTAGTACATTCGCAAGATACATGGCTGATACATTTAGAGACCTGAGATTCGTCAATGTTTACCTTGATGATATATTAATATTCTCCGAATCTCCAGAAGAACATTGGAAACATTTAGACACGGTACTAGAAAGATTAAAGAACGAGAACCTCATTGTTAAGAAGAAAAAATGTAAATTTGCATCTGAAGAAACTGAGTTTTTAGGCTATAGTATTGGAATCCAGAAAATAGCTCCACTACAGCACAAATGTGCAGCAATCCGAGACTTTCCGACGCCTAAAACAGTAAAACAAGCACAGAGATTTTTAGGAATGATTAATTACTACAGACGATTCATTCCAAATTGCTCCAAGATTGCACAGCCAATCCAACTGTTTATTTGTGACAAAAGTCAATGGACAGAAAAACAAGACAAGGCAATTGATAAACTAAAAGACGCCTTGTGTAACTCCCCCGTCCTAGTACCATTCAACAACAAAGCAAACTACCGACTTACAACAGACGCCTCAAAAGACGGCATTGGTGCTGTTCTAGAAGAAGTCGACAACAAGAACAAACTTGTTGGTGTCGTCGGTTACTTCTCTAAATCCTTAGAGAGTGCCCAGAAAAACTATCCTGCTGGCGAATTAGAACTACTTGGAATTATCAAAGCACTCCACCACTTCCGATATATGCTTCACGGAAAGCATTTCACGTTAAGAACAGACCACATTAGTTTGTTATCATTACAAAACAAGAACGAACCCGACGAACGCGTGCAACGCTGGTTAGATGACCTAGCCACATATGACTTCACCTTAGAATACCTAGCTGGACCCAAGAACGTTGTCGCAGATGCCATATCCCGTGCCGTATATACTATAACCCCCGAAACATCCCGACCTATCGACACAGAAAGCTGGAAATCTTACTACAAATCAGACCCATTATGTAGTGCTGTCTTAATTCATATGAAAGAATTGACACAACACAACGTCACACCTGAAGATATGTCAGCCTTCCGTAGTTACCAGAAGAAACTCGAACTATCAGAGACCTTCCGAAAGAATTATTCCCTAGAAGACGAAATGATCTATTACCAAGACCGACTAGTAGTACCAATAAAACAACAGAACGCAGTTATGAGACTATATCATGACCATACCTTATTTGGAGGACATTTTGGTGTAACAGTGACCCTTGCGAAAATCAGCCCAATTTACTATTGGCCAAAATTACAACATTCGATCATACAATACATCAGGACCTGCGTACAATGTCAACTAATAAAATCACACCGACCACGCTTACATGGACTATTACAACCACTCCCTATAGCAGAAGGAAGATGGCTTGATATATCAATGGATTTTGTGACAGGATTACCCCCGACATCAAATAACTTGAATATGATCCTCGTCGTAGTTGATCGTTTTTCGAAACGCGCTCACTTCATAGCTACAAGGAAAACCTTAGACGCAACACAACTAATAGATCTACTCTTTCGATACATTTTTTCATATCATGGTTTTCCCAGGACAATAACCAGTGATAGAGATGTCCGTATGACCGCCGACAAATATCAAGAACTCACGAAAAGACTAGGAATAAAATCGACAATGTCTTCCGCGAACCACCCCCAAACAGATGGACAATCCGAACGAACGATACAGACATTAAACAGGTTACTAAGAGCCTATGCTTCAACCAATATTCAGAATTGGCATGTATATTTACCACAAATCGAATTTGTTTACAATTCTACACCTACTAGAACACTTGGAAAATCACCATTTGAAATTGATTTAGGATATTTACCGAATACCCCTGCTATTAAGTCAGATGACGAAGTCAACGCAAGAAGTTTTACTGCCGTAGAACTTGCCAAACACCTCAAAGCCCTTACCATCCAAACGAAGGAACAGCTAGAACACGCTCAAATCGAAATGGAAACTAATAACAATCAAAGACGTAAACCCTTATTGTTAAACATAGGAGATCACGTATTAGTGCATAGAGATGCATACTTCAAGAAAGGTGCTTATATGAAAGTACAACAAATATACGTCGGACCATTTCGAGTTGTCAAGAAAATAAACGATAACGCCTACGAACTAGATTTAAACTCTCACAAGAAAAAGCACAGAGTTATTAATGTACAATTCCTGAAAAAGTTTGTATACCGTCCAGACGCGTACCCAAAGAATAAACCAATCAGCTCCACTGAAAGAATTAAGAGAGCACACGAAGTTACTGCACTCATAGGAATAGATACTACACACAAAACTTACTTATGTCACATGCAAGATGTAGACCCAACACTTTCAGTAGAATACTCAGAAGCTGAATTTTGCCAAATTCCCGAAAGAACACGAAGATCAATATTAGCCAACTTTAGACAACTCTACGAAACACAAGACAACCCTGAGAGAGAGGAAGATGTTGTATCTCAAAATGAGATATGTCAGTATGACAATACGTCACCCGGGATGGTGAGCAAGGGCGAGGAGGATAACATGGCCATCATCAAGGAGTTCATGCGCTTCAAGGTGCACATGGAGGGCTCCGTGAACGGCCACGAGTTCGAGATCGAGGGCGAGGGCGAGGGCCGCCCCTACGAGGGCACCCAGACCGCCAAGCTGAAGGTGACCAAGGGTGGCCCCCTGCCCTTCGCCTGGGACATCCTGTCCCCTCAGTTCATGTACGGCTCCAAGGCCTACGTGAAGCACCCCGCCGACATCCCCGACTACTTGAAGCTGTCCTTCCCCGAGGGCTTCAAGTGGGAGCGCGTGATGAACTTCGAGGACGGCGGCGTGGTGACCGTGACCCAGGACTCCTCCCTGCAGGACGGCGAGTTCATCTACAAGGTGAAGCTGCGCGGCACCAACTTCCCCTCCGACGGCCCCGTAATGCAGAAGAAGACCATGGGCTGGGAGGCCTCCTCCGAGCGGATGTACCCCGAGGACGGCGCCCTGAAGGGCGAGATCAAGCAGAGGCTGAAGCTGAAGGACGGCGGCCACTACGACGCTGAGGTCAAGACCACCTACAAGGCCAAGAAGCCCGTGCAGCTGCCCGGCGCCTACAACGTCAACATCAAGTTGGACATCACCTCCCACAACGAGGACTACACCATCGTGGAACAGTACGAACGCGCCGAGGGCCGCCACTCCACCGGCGGCATGGACGAGCTGTACAAGTAGCCCGGGACGTTCATAAAACACATATGAAACAACCTTATAACAAAACGAACAACATGAGACAAAACCCGACCTTCCCTAGCTGAACTACCCAAAGTATAAATGCCTGAACAATTAGTTTAGATCCGAGATTCCGCGCTTCCACCACTTAGTATGATTCATATTTTATATAATATATAAGATAAGTAACATTCCGTGAATTAATCTGATAAACTGTTTTGACAACTGGTTACTTCCCTAAGACTGTTTATATTAGGATGTCAAGACACTCCGGTATTTACTCGAGCCCGTAATACAACA**GCGGCCGCCACCGCGGTGGAGCTCCAATTCGCCCTATAGTGAGTCGTATTACAATTCACTGGCCGTCGTTTTACAACGTCGTGACTGGGAAAACCCTGGCGTTACCCAACTTAATCGCCTTGCAGCACATCCCCCCTTCGCCAGCTGGCGTAATAGCGAAGAGGCCCGCACCGATCGCCCTTCCCAACAGTTGCGCAGCCTGAATGGCGAATGGCGCGACGCGCCCTGTAGCGGCGCATTAAGCGCGGCGGGTGTGGTGGTTACGCGCAGCGTGACCGCTACACTTGCCAGCGCCCTAGCGCCCGCTCCTTTCGCTTTCTTCCCTTCCTTTCTCGCCACGTTCGCCGGCTTTCCCCGTCAAGCTCTAAATCGGGGGCTCCCTTTAGGGTTCCGATTTAGTGCTTTACGGCACCTCGACCCCAAAAAACTTGATTAGGGTGATGGTTCACGTAGTGGGCCATCGCCCTGATAGACGGTTTTTCGCCCTTTGACGTTGGAGTCCACGTTCTTTAATAGTGGACTCTTGTTCCAAACTGGAACAACACTCAACCCTATCTCGGTCTATTCTTTTGATTTATAAGGGATTTTGCCGATTTCGGCCTATTGGTTAAAAAATGAGCTGATTTAACAAAAATTTAACGCGAATTTTAACAAAATATTAACGTTTACAATTTCCTGATGCGGTATTTTCTCCTTACGCATCTGTGCGGTATTTCACACCGCAGGGTAATAACTGATATAATTAAATTGAAGCTCTAATTTGTGAGTTTAGTATACATGCATTTACTTATAATACAGTTTTTTAGTTTTGCTGGCCGCATCTTCTCAAATATGCTTCCCAGCCTGCTTTTCTGTAACGTTCACCCTCTACCTTAGCATCCCTTCCCTTTGCAAATAGTCCTCTTCCAACAATAATAATGTCAGATCCTGTAGAGACCACATCATCCACGGTTCTATACTGTTGACCCAATGCGTCTCCCTTGTCATCTAAACCCACACCGGGTGTCATAATCAACCAATCGTAACCTTCATCTCTTCCACCCATGTCTCTTTGAGCAATAAAGCCGATAACAAAATCTTTGTCGCTCTTCGCAATGTCAACAGTACCCTTAGTATATTCTCCAGTAGATAGGGAGCCCTTGCATGACAATTCTGCTAACATCAAAAGGCCTCTAGGTTCCTTTGTTACTTCTTCTGCCGCCTGCTTCAAACCGCTAACAATACCTGGGCCCACCACACCGTGTGCATTCGTAATGTCTGCCCATTCTGCTATTCTGTATACACCCGCAGAGTACTGCAATTTGACTGTATTACCAATGTCAGCAAATTTTCTGTCTTCGAAGAGTAAAAAATTGTACTTGGCGGATAATGCCTTTAGCGGCTTAACTGTGCCCTCCATGGAAAAATCAGTCAAGATATCCACATGTGTTTTTAGTAAACAAATTTTGGGACCTAATGCTTCAACTAACTCCAGTAATTCCTTGGTGGTACGAACATCCAATGAAGCACACAAGTTTGTTTGCTTTTCGTGCATGATATTAAATAGCTTGGCAGCAACAGGACTAGGATGAGTAGCAGCACGTTCCTTATATGTAGCTTTCGACATGATTTATCTTCGTTTCCTGCAGGTTTTTGTTCTGTGCAGTTGGGTTAAGAATACTGGGCAATTTCATGTTTCTTCAACACTACATATGCGTATATATACCAATCTAAGTCTGTGCTCCTTCCTTCGTTCTTCCTTCTGTTCGGAGATTACCGAATCAAAAAAATTTCAAAGAAACCGAAATCAAAAAAAAGAATAAAAAAAAAATGATGAATTGAATTGAAAAGCGTGGTGCACTCTCAGTACAATCTGCTCTGATGCCGCATAGTTAAGCCAGCCCCGACACCCGCCAACACCCGCTGACGCGCCCTGACGGGCTTGTCTGCTCCCGGCATCCGCTTACAGACAAGCTGTGACCGTCTCCGGGAGCTGCATGTGTCAGAGGTTTTCACCGTCATCACCGAAACGCGCGA

pTD3685

Contents in bold: Ty3

Genetic markers: *URA3*, *CEN6/ARS4*, Amp^R^

Construction: Ty3 *Sal*I-*Not*I fragment was amplified from pNB2176 (Beliakova-Bethell N, Beckham C, Giddings TH, Jr., Winey M, Parker R, et al. Virus-like particles of the Ty3 retrotransposon assemble in association with P-body components. RNA. 2006; 12: 94-101) using primers VB3465 (F5- CGCCTCAAAAGACGGCATTGGTGCTGTTCTAGAAGAAGTCGACAACAAGAACAAACTTGTTGG -3) and VB3466 (F5- ACGACTCACTATAGGGCGAATTGGAGCTCCACCGCGGTGGCGGCCGCTGTTGTATTACGGGCTCG-3; introduces *Not*I site, underlined). The fragment was inserted into *Sal*I and *Not*1-digested pTD3548.

GACGAAAGGGCCTCGTGATACGCCTATTTTTATAGGTTAATGTCATGATAATAATGGTTTCTTAGGACGGATCGCTTGCCTGTAACTTACACGCGCCTCGTATCTTTTAATGATGGAATAATTTGGGAATTTACTCTGTGTTTATTTATTTTTATGTTTTGTATTTGGATTTTAGAAAGTAAATAAAGAAGGTAGAAGAGTTACGGAATGAAGAAAAAAAAATAAACAAAGGTTTAAAAAATTTCAACAAAAAGCGTACTTTACATATATATTTATTAGACAAGAAAAGCAGATTAAATAGATATACATTCGATTAACGATAAGTAAAATGTAAAATCACAGGATTTTCGTGTGTGGTCTTCTACACAGACAAGATGAAACAATTCGGCATTAATACCTGAGAGCAGGAAGAGCAAGATAAAAGGTAGTATTTGTTGGCGATCCCCCTAGAGTCTTTTACATCTTCGGAAAACAAAAACTATTTTTTCTTTAATTTCTTTTTTTACTTTCTATTTTTAATTTATATATTTATATTAAAAAATTTAAATTATAATTATTTTTATAGCACGTGATGAAAAGGACCCAGGTGGCACTTTTCGGGGAAATGTGCGCGGAACCCCTATTTGTTTATTTTTCTAAATACATTCAAATATGTATCCGCTCATGAGACAATAACCCTGATAAATGCTTCAATAATATTGAAAAAGGAAGAGTATGAGTATTCAACATTTCCGTGTCGCCCTTATTCCCTTTTTTGCGGCATTTTGCCTTCCTGTTTTTGCTCACCCAGAAACGCTGGTGAAAGTAAAAGATGCTGAAGATCAGTTGGGTGCACGAGTGGGTTACATCGAACTGGATCTCAACAGCGGTAAGATCCTTGAGAGTTTTCGCCCCGAAGAACGTTTTCCAATGATGAGCACTTTTAAAGTTCTGCTATGTGGCGCGGTATTATCCCGTATTGACGCCGGGCAAGAGCAACTCGGTCGCCGCATACACTATTCTCAGAATGACTTGGTTGAGTACTCACCAGTCACAGAAAAGCATCTTACGGATGGCATGACAGTAAGAGAATTATGCAGTGCTGCCATAACCATGAGTGATAACACTGCGGCCAACTTACTTCTGACAACGATCGGAGGACCGAAGGAGCTAACCGCTTTTTTTCACAACATGGGGGATCATGTAACTCGCCTTGATCGTTGGGAACCGGAGCTGAATGAAGCCATACCAAACGACGAGCGTGACACCACGATGCCTGTAGCAATGGCAACAACGTTGCGCAAACTATTAACTGGCGAACTACTTACTCTAGCTTCCCGGCAACAATTAATAGACTGGATGGAGGCGGATAAAGTTGCAGGACCACTTCTGCGCTCGGCCCTTCCGGCTGGCTGGTTTATTGCTGATAAATCTGGAGCCGGTGAGCGTGGGTCTCGCGGTATCATTGCAGCACTGGGGCCAGATGGTAAGCCCTCCCGTATCGTAGTTATCTACACGACGGGCAGTCAGGCAACTATGGATGAACGAAATAGACAGATCGCTGAGATAGGTGCCTCACTGATTAAGCATTGGTAACTGTCAGACCAAGTTTACTCATATATACTTTAGATTGATTTAAAACTTCATTTTTAATTTAAAAGGATCTAGGTGAAGATCCTTTTTGATAATCTCATGACCAAAATCCCTTAACGTGAGTTTTCGTTCCACTGAGCGTCAGACCCCGTAGAAAAGATCAAAGGATCTTCTTGAGATCCTTTTTTTCTGCGCGTAATCTGCTGCTTGCAAACAAAAAAACCACCGCTACCAGCGGTGGTTTGTTTGCCGGATCAAGAGCTACCAACTCTTTTTCCGAAGGTAACTGGCTTCAGCAGAGCGCAGATACCAAATACTGTCCTTCTAGTGTAGCCGTAGTTAGGCCACCACTTCAAGAACTCTGTAGCACCGCCTACATACCTCGCTCTGCTAATCCTGTTACCAGTGGCTGCTGCCAGTGGCGATAAGTCGTGTCTTACCGGGTTGGACTCAAGACGATAGTTACCGGATAAGGCGCAGCGGTCGGGCTGAACGGGGGGTTCGTGCACACAGCCCAGCTTGGAGCGAACGACCTACACCGAACTGAGATACCTACAGCGTGAGCATTGAGAAAGCGCCACGCTTCCCGAAGGGAGAAAGGCGGACAGGTATCCGGTAAGCGGCAGGGTCGGAACAGGAGAGCGCACGAGGGAGCTTCCAGGGGGGAACGCCTGGTATCTTTATAGTCCTGTCGGGTTTCGCCACCTCTGACTTGAGCGTCGATTTTTGTGATGCTCGTCAGGGGGGCCGAGCCTATGGAAAAACGCCAGCAACGCGGCCTTTTTACGGTTCCTGGCCTTTTGCTGGCCTTTTGCTCACATGTTCTTTCCTGCGTTATCCCCTGATTCTGTGGATAACCGTATTACCGCCTTTGAGTGAGCTGATACCGCTCGCCGCAGCCGAACGACCGAGCGCAGCGAGTCAGTGAGCGAGGAAGCGGAAGAGCGCCCAATACGCAAACCGCCTCTCCCCGCGCGTTGGCCGATTCATTAATGCAGCTGGCACGACAGGTTTCCCGACTGGAAAGCGGGCAGTGAGCGCAACGCAATTAATGTGAGTTAGCTCACTCATTAGGCACCCCAGGCTTTACACTTTATGCTTCCGGCTCGTATGTTGTGTGGAATTGTGAGCGGATAACAATTTCACACAGGAAACAGCTATGACCATGATTACGCCAAGCTCGAAATTAACCCTCACTAAAGGGAACAAAAGCTGTCGAGACGGTATCGATAAGCTT**TGTTGTATCTCAAAATGAGATATGTCAGTATGACAATACGTCACCCTGAACGTTCATAAAACACATATGAAACAACCTTATAACAAAACGAACAACATGAGACAAAACCCGACCTTCCCTAGCTGAACTACCCAAAGTATAAATGCCTGAACAATTAGTTTAGATCCGAGATTCCGCGCTTCCACCACTTAGTATGATTCATATTTTATATAATATATAAGATAAGTAACATTCCGTGAATTAATCTGATAAACTGTTTTGACAACTGGTTACTTCCCTAAGACTGTTTATATTAGGATTGTCAAGACACTCCGGTATTACTCGAGCCCGTAATACAACACCTGGTAGCGTTAAAGGTTACTAATTGTTCAAACGAACCATCGAAAAGCCGAACCTAGCTACACCACACCCCAGTATGAGCTTTATGGATCAAATCCCAGGAGGAGGAAATTATCCAAAACTCCCAGTAGAATGCCTTCCTAACTTCCCGATCCAACCATCTTTGACCTTCAGAGGTAGAAATGACTCGCATAAACTGAAAAACTTTATCTCCGAAATAATGTTAAACATGTCTATGATATCTTGGCCGAATGATGCCAGTCGTATTGTGTACTGCAGAAGACATTTATTAAACCCCGCTGCTCAGTGGGCTAATGACTTTGTACAAGAACAAGGTATACTTGAAATAACATTCGACACATTCATACAAGGATTATATCAGCATTTCTATAAGCCACCAGATATCAATAAAATCTTTAATGCAATCACGCAACTTTCCGAAGCTAAACTTGGTATTGAGCGTCTCAACCAACGATTCAGAAAGATTTGGGACAGAATGCCACCAGACTTCATGACCGAAAAAGCTGCCATAATGACATATACTAGGCTATTGACAAAGGAAACCTATAATATTGTCAGAATGCACAAACCAGAGACATTAAAAGACGCCATGGAAGAGGCTTACCAGACAACTGCACTAACTGAAAGATTCTTCCCAGGATTCGAACTTGATGCTGATGGAGACACTATCATCGGTGCCACAACCCACTTACAAGAAGAATACGACTCTGACTATGATTCAGAAGATAATCTGACCCAGAATGGATACGTCCATACCGTAAGGACAAGAAGATCTTACAATAAACCAATGTCAAATCATCGAAACAGGAGAAATAACAACCCATCTAGAGAAGAATGTATAAAAAATCGGCTATGCTTCTATTGTAAGAAAGAGGGACATCGCCTGAACGAATGTAGAGCACGTAAGGCGAGTTCTAACCGATCTTGAACTCGAATCAAAAGACCAACAAACTCCTTTTATCAAAACCTTACCAATTGTACACTATATCGCCATCCCCGAGATGGACAATACCGCCGAAAAAACCATAAAAATACAAAACACGAAAGTAAAAACCCTGTTTGACAGTGGATCACCCACGTCATTTATCCGAAGAGATATTGTAGAACTTCTCAAATACGAAATCTACGAGACCCCTCCACTCCGTTTTAGAGGATTCGTAGCCACCAAATCCGCCGTTACATCCGAAGCAGTCACCATTGACCTCAAAATCAATGACCTGCATATAACTTTAGCCGCGTACATACTGGATAACATGGACTACCAATTGTTAATTGGAAATCCAATCTTACGCCGCTACCCGAAAATCCTGCACACAGTACTGAATACCAGAGAGAGCCCCGACTCCTTAAAGCCCAAGACTTATCGCTCCGAAACCGTTAATAACGTTAGAACCTACTCCGCTGGTAATCGTGGTAACCCCAGAAACATAAAACTGTCTTTTGCCCCCACCATTCTCGAAGCAACTGACCCGAAATCCGCTGGTAATCGTGGTGACTCCAGAACCAAAACCCTGTCTCTTGCAACCACTACTCCTGCAGCAATTGACCCGCTTACGACCCTTGATAACCCAGGTAGTACTCAAAGTACATTTGCGCAATTCCCGATACCTGAAGAAGCGAGCATCCTAGAAGAGGATGGAAAATACTCCAACGTTGTCTCAACCATTCAGAGTGTAGAACCTAATGCTACTGATCACAGCAATAAGGACACCTTTTGCACTTTGCCAGTTTGGTTACAACAGAAGTATAGAGAGATCATACGTAATGATCTCCCACCAAGACCTGCCGACATTAATAACATCCCCGTAAAACATGATATTGAAATTAAACCTGGCGCAAGACTACCTCGACTACAGCCATACCATGTTACAGAAAAGAACGAACAAGAAATCAACAAAATAGTTCAAAAACTGCTCGATAACAAGTTCATTGTTCCCTCAAAGTCGCCTTGCAGCTCCCCTGTAGTCCTCGTCCCGAAGAAAGACGGTACCTTCCGACTCTGCGTCGATTACCGCACCCTGAACAAAGCTACCATCTCCGACCCATTCCCATTACCCAGAATCGACAACCTATTGAGCCGTATTGGAAATGCCCAGATATTTACCACGCTAGATTTGCATAGTGGTTACCACCAGATCCCGATGGAACCCAAAGACCGCTACAAAACCGCCTTTGTCACACCATCCGGTAAGTATGAATATACCGTCATGCCATTTGGCTTAGTCAATGCACCTAGTACATTCGCAAGATACATGGCTGATACATTTAGAGACCTGAGATTCGTCAATGTTTACCTTGATGATATATTAATATTCTCCGAATCTCCAGAAGAACATTGGAAACATTTAGACACGGTACTAGAAAGATTAAAGAACGAGAACCTCATTGTTAAGAAGAAAAAATGTAAATTTGCATCTGAAGAAACTGAGTTTTTAGGCTATAGTATTGGAATCCAGAAAATAGCTCCACTACAGCACAAATGTGCAGCAATCCGAGACTTTCCGACGCCTAAAACAGTAAAACAAGCACAGAGATTTTTAGGAATGATTAATTACTACAGACGATTCATTCCAAATTGCTCCAAGATTGCACAGCCAATCCAACTGTTTATTTGTGACAAAAGTCAATGGACAGAAAAACAAGACAAGGCAATTGATAAACTAAAAGACGCCTTGTGTAACTCCCCCGTCCTAGTACCATTCAACAACAAAGCAAACTACCGACTTACAACAGACGCCTCAAAAGACGGCATTGGTGCTGTTCTAGAAGAAGTCGACAACAAGAACAAACTTGTTGGTGTCGTCGGTTACTTCTCTAAATCCTTAGAGAGTGCCCAGAAAAACTATCCTGCTGGCGAATTAGAACTACTTGGAATTATCAAAGCACTCCACCACTTCCGATATATGCTTCACGGAAAGCATTTCACGTTAAGAACAGACCACATTAGTTTGTTATCATTACAAAACAAGAACGAACCCGCACGACGCGTGCAACGCTGGTTAGATGACCTAGCCACATATGACTTCACCTTAGAATACCTAGCTGGACCCAAGAACGTTGTCGCAGATGCCATATCCCGTGCCGTATATACTATAACCCCCGAAACATCCCGACCTATCGACACAGAAAGCTGGAAATCTTACTACAAATCAGACCCATTATGTAGTGCTGTCTTAATTCATATGAAAGAATTGACACAACACAACGTCACACCTGAAGATATGTCAGCCTTCCGTAGTTACCAGAAGAAACTCGAACTATCAGAGACCTTCCGAAAGAATTATTCCCTAGAAGACGAAATGATCTATTACCAAGACCGACTAGTAGTACCAATAAAACAACAGAACGCAGTTATGAGACTATATCATGACCATACCTTATTTGGAGGACATTTTGGTGTAACAGTGACCCTTGCGAAAATCAGCCCAATTTACTATTGGCCAAAATTACAACATTCGATCATACAATACATCAGGACCTGCGTACAATGTCAACTAATAAAATCACACCGACCACGCTTACATGGACTATTACAACCACTCCCTATAGCAGAAGGAAGATGGCTTGATATATCAATGGATTTTGTGACAGGATTACCCCCGACATCAAATAACTTGAATATGATCCTCGTCGTAGTTGATCGTTTTTCGAAACGCGCTCACTTCATAGCTACAAGGAAAACCTTAGACGCAACACAACTAATAGATCTACTCTTTCGATACATTTTTTCATATCATGGTTTTCCCAGGACAATAACCAGTGATAGAGATGTCCGTATGACCGCCGACAAATATCAAGAACTCACGAAAAGACTAGGAATAAAATCGACAATGTCTTCCGCGAACCACCCCCAAACAGATGGACAATCCGAACGAACGATACAGACATTAAACAGGTTACTAAGAGCCTATGCTTCAACCAATATTCAGAATTGGCATGTATATTTACCACAAATCGAATTTGTTTACAATTCTACACCTACTAGAACACTTGGAAAATCACCATTTGAAATTGATTTAGGATATTTACCGAATACCCCTGCTATTAAGTCAGATGACGAAGTCAACGCAAGAAGTTTTACTGCCGTAGAACTTGCCAAACACCTCAAAGCCCTTACCATCCAAACGAAGGAACAGCTAGAACACGCTCAAATCGAAATGGAAACTAATAACAATCAAAGACGTAAACCCTTATTGTTAAACATAGGAGATCACGTATTAGTGCATAGAGATGCATACTTCAAGAAAGGTGCTTATATGAAAGTACAACAAATATACGTCGGACCATTTCGAGTTGTCAAGAAAATAAACGATAACGCCTACGAACTAGATTTAAACTCTCACAAGAAAAAGCACAGAGTTATTAATGTACAATTCCTGAAAAAGTTTGTATACCGTCCAGACGCGTACCCAAAGAATAAACCAATCAGCTCCACTGAAAGAATTAAGAGAGCACACGAAGTTACTGCACTCATAGGAATAGATACTACACACAAAACTTACTTATGTCACATGCAAGATGTAGACCCAACACTTTCAGTAGAATACTCAGAAGCTGAATTTTGCCAAATTCCCGAAAGAACACGAAGATCAATATTAGCCAACTTTAGACAACTCTACGAAACACAAGACAACCCTGAGAGAGAGGAAGATGTTGTATCTCAAAATGAGATATGTCAGTATGACAATACGTCACCCTGAACGTTCATAAAACACATATGAAACAACCTTATAACAAAACGAACAACATGAGACAAAACCCGACCTTCCCTAGCTGAACTACCCAAAGTATAAATGCCTGAACAATTAGTTTAGATCCGAGATTCCGCGCTTCCACCACTTAGTATGATTCATATTTTATATAATATATAAGATAAGTAACATTCCGTGAATTAATCTGATAAACTGTTTTGACAACTGGTTACTTCCCTAAGACTGTTTATATTAGGATTGTCAAGACACTCCGGTATTACTCGAGCCCGTAATACAACA**GCGGCCGCCACCGCGGTGGAGCTCCAATTCGCCCTATAGTGAGTCGTATTACAATTCACTGGCCGTCGTTTTACAACGTCGTGACTGGGAAAACCCTGGCGTTACCCAACTTAATCGCCTTGCAGCACATCCCCCCTTCGCCAGCTGGCGTAATAGCGAAGAGGCCCGCACCGATCGCCCTTCCCAACAGTTGCGCAGCCTGAATGGCGAATGGCGCGACGCGCCCTGTAGCGGCGCATTAAGCGCGGCGGGTGTGGTGGTTACGCGCAGCGTGACCGCTACACTTGCCAGCGCCCTAGCGCCCGCTCCTTTCGCTTTCTTCCCTTCCTTTCTCGCCACGTTCGCCGGCTTTCCCCGTCAAGCTCTAAATCGGGGGCTCCCTTTAGGGTTCCGATTTAGTGCTTTACGGCACCTCGACCCCAAAAAACTTGATTAGGGTGATGGTTCACGTAGTGGGCCATCGCCCTGATAGACGGTTTTTCGCCCTTTGACGTTGGAGTCCACGTTCTTTAATAGTGGACTCTTGTTCCAAACTGGAACAACACTCAACCCTATCTCGGTCTATTCTTTTGATTTATAAGGGATTTTGCCGATTTCGGCCTATTGGTTAAAAAATGAGCTGATTTAACAAAAATTTAACGCGAATTTTAACAAAATATTAACGTTTACAATTTCCTGATGCGGTATTTTCTCCTTACGCATCTGTGCGGTATTTCACACCGCAGGGTAATAACTGATATAATTAAATTGAAGCTCTAATTTGTGAGTTTAGTATACATGCATTTACTTATAATACAGTTTTTTAGTTTTGCTGGCCGCATCTTCTCAAATATGCTTCCCAGCCTGCTTTTCTGTAACGTTCACCCTCTACCTTAGCATCCCTTCCCTTTGCAAATAGTCCTCTTCCAACAATAATAATGTCAGATCCTGTAGAGACCACATCATCCACGGTTCTATACTGTTGACCCAATGCGTCTCCCTTGTCATCTAAACCCACACCGGGTGTCATAATCAACCAATCGTAACCTTCATCTCTTCCACCCATGTCTCTTTGAGCAATAAAGCCGATAACAAAATCTTTGTCGCTCTTCGCAATGTCAACAGTACCCTTAGTATATTCTCCAGTAGATAGGGAGCCCTTGCATGACAATTCTGCTAACATCAAAAGGCCTCTAGGTTCCTTTGTTACTTCTTCTGCCGCCTGCTTCAAACCGCTAACAATACCTGGGCCCACCACACCGTGTGCATTCGTAATGTCTGCCCATTCTGCTATTCTGTATACACCCGCAGAGTACTGCAATTTGACTGTATTACCAATGTCAGCAAATTTTCTGTCTTCGAAGAGTAAAAAATTGTACTTGGCGGATAATGCCTTTAGCGGCTTAACTGTGCCCTCCATGGAAAAATCAGTCAAGATATCCACATGTGTTTTTAGTAAACAAATTTTGGGACCTAATGCTTCAACTAACTCCAGTAATTCCTTGGTGGTACGAACATCCAATGAAGCACACAAGTTTGTTTGCTTTTCGTGCATGATATTAAATAGCTTGGCAGCAACAGGACTAGGATGAGTAGCAGCACGTTCCTTATATGTAGCTTTCGACATGATTTATCTTCGTTTCCTGCAGGTTTTTGTTCTGTGCAGTTGGGTTAAGAATACTGGGCAATTTCATGTTTCTTCAACACTACATATGCGTATATATACCAATCTAAGTCTGTGCTCCTTCCTTCGTTCTTCCTTCTGTTCGGAGATTACCGAATCAAAAAAATTTCAAAGAAACCGAAATCAAAAAAAAGAATAAAAAAAAAATGATGAATTGAATTGAAAAGCGTGGTGCACTCTCAGTACAATCTGCTCTGATGCCGCATAGTTAAGCCAGCCCCGACACCCGCCAACACCCGCTGACGCGCCCTGACGGGCTTGTCTGCTCCCGGCATCCGCTTACAGACAAGCTGTGACCGTCTCCGGGAGCTGCATGTGTCAGAGGTTTTCACCGTCATCACCGAAACGCGCGA

pPS3705

Contents in bold: Ty3 (NCΔ)

Genetic markers: *URA3*, *CEN6/ARS4*, Amp^R^

Construction: Ty3 *Sex*AI-*Kpn*I fragment from pNB2622 with deletion of Ty3 sequence encoding aa 237-281 (Larsen LS, Beliakova-Bethell N, Bilanchone V, Zhang M, Lamsa A, et al. Ty3 nucleocapsid controls localization of particle assembly. J Virol. 2008; 82: 2501-2514) was inserted into *Sex*AI and *Kpn*I-digested pTD3685.

GACGAAAGGGCCTCGTGATACGCCTATTTTTATAGGTTAATGTCATGATAATAATGGTTTCTTAGGACGGATCGCTTGCCTGTAACTTACACGCGCCTCGTATCTTTTAATGATGGAATAATTTGGGAATTTACTCTGTGTTTATTTATTTTTATGTTTTGTATTTGGATTTTAGAAAGTAAATAAAGAAGGTAGAAGAGTTACGGAATGAAGAAAAAAAAATAAACAAAGGTTTAAAAAATTTCAACAAAAAGCGTACTTTACATATATATTTATTAGACAAGAAAAGCAGATTAAATAGATATACATTCGATTAACGATAAGTAAAATGTAAAATCACAGGATTTTCGTGTGTGGTCTTCTACACAGACAAGATGAAACAATTCGGCATTAATACCTGAGAGCAGGAAGAGCAAGATAAAAGGTAGTATTTGTTGGCGATCCCCCTAGAGTCTTTTACATCTTCGGAAAACAAAAACTATTTTTTCTTTAATTTCTTTTTTTACTTTCTATTTTTAATTTATATATTTATATTAAAAAATTTAAATTATAATTATTTTTATAGCACGTGATGAAAAGGACCCAGGTGGCACTTTTCGGGGAAATGTGCGCGGAACCCCTATTTGTTTATTTTTCTAAATACATTCAAATATGTATCCGCTCATGAGACAATAACCCTGATAAATGCTTCAATAATATTGAAAAAGGAAGAGTATGAGTATTCAACATTTCCGTGTCGCCCTTATTCCCTTTTTTGCGGCATTTTGCCTTCCTGTTTTTGCTCACCCAGAAACGCTGGTGAAAGTAAAAGATGCTGAAGATCAGTTGGGTGCACGAGTGGGTTACATCGAACTGGATCTCAACAGCGGTAAGATCCTTGAGAGTTTTCGCCCCGAAGAACGTTTTCCAATGATGAGCACTTTTAAAGTTCTGCTATGTGGCGCGGTATTATCCCGTATTGACGCCGGGCAAGAGCAACTCGGTCGCCGCATACACTATTCTCAGAATGACTTGGTTGAGTACTCACCAGTCACAGAAAAGCATCTTACGGATGGCATGACAGTAAGAGAATTATGCAGTGCTGCCATAACCATGAGTGATAACACTGCGGCCAACTTACTTCTGACAACGATCGGAGGACCGAAGGAGCTAACCGCTTTTTTTCACAACATGGGGGATCATGTAACTCGCCTTGATCGTTGGGAACCGGAGCTGAATGAAGCCATACCAAACGACGAGCGTGACACCACGATGCCTGTAGCAATGGCAACAACGTTGCGCAAACTATTAACTGGCGAACTACTTACTCTAGCTTCCCGGCAACAATTAATAGACTGGATGGAGGCGGATAAAGTTGCAGGACCACTTCTGCGCTCGGCCCTTCCGGCTGGCTGGTTTATTGCTGATAAATCTGGAGCCGGTGAGCGTGGGTCTCGCGGTATCATTGCAGCACTGGGGCCAGATGGTAAGCCCTCCCGTATCGTAGTTATCTACACGACGGGCAGTCAGGCAACTATGGATGAACGAAATAGACAGATCGCTGAGATAGGTGCCTCACTGATTAAGCATTGGTAACTGTCAGACCAAGTTTACTCATATATACTTTAGATTGATTTAAAACTTCATTTTTAATTTAAAAGGATCTAGGTGAAGATCCTTTTTGATAATCTCATGACCAAAATCCCTTAACGTGAGTTTTCGTTCCACTGAGCGTCAGACCCCGTAGAAAAGATCAAAGGATCTTCTTGAGATCCTTTTTTTCTGCGCGTAATCTGCTGCTTGCAAACAAAAAAACCACCGCTACCAGCGGTGGTTTGTTTGCCGGATCAAGAGCTACCAACTCTTTTTCCGAAGGTAACTGGCTTCAGCAGAGCGCAGATACCAAATACTGTCCTTCTAGTGTAGCCGTAGTTAGGCCACCACTTCAAGAACTCTGTAGCACCGCCTACATACCTCGCTCTGCTAATCCTGTTACCAGTGGCTGCTGCCAGTGGCGATAAGTCGTGTCTTACCGGGTTGGACTCAAGACGATAGTTACCGGATAAGGCGCAGCGGTCGGGCTGAACGGGGGGTTCGTGCACACAGCCCAGCTTGGAGCGAACGACCTACACCGAACTGAGATACCTACAGCGTGAGCATTGAGAAAGCGCCACGCTTCCCGAAGGGAGAAAGGCGGACAGGTATCCGGTAAGCGGCAGGGTCGGAACAGGAGAGCGCACGAGGGAGCTTCCAGGGGGGAACGCCTGGTATCTTTATAGTCCTGTCGGGTTTCGCCACCTCTGACTTGAGCGTCGATTTTTGTGATGCTCGTCAGGGGGGCCGAGCCTATGGAAAAACGCCAGCAACGCGGCCTTTTTACGGTTCCTGGCCTTTTGCTGGCCTTTTGCTCACATGTTCTTTCCTGCGTTATCCCCTGATTCTGTGGATAACCGTATTACCGCCTTTGAGTGAGCTGATACCGCTCGCCGCAGCCGAACGACCGAGCGCAGCGAGTCAGTGAGCGAGGAAGCGGAAGAGCGCCCAATACGCAAACCGCCTCTCCCCGCGCGTTGGCCGATTCATTAATGCAGCTGGCACGACAGGTTTCCCGACTGGAAAGCGGGCAGTGAGCGCAACGCAATTAATGTGAGTTAGCTCACTCATTAGGCACCCCAGGCTTTACACTTTATGCTTCCGGCTCGTATGTTGTGTGGAATTGTGAGCGGATAACAATTTCACACAGGAAACAGCTATGACCATGATTACGCCAAGCTCGAAATTAACCCTCACTAAAGGGAACAAAAGCTGTCGAGACGGTATCGATAAGCTT**TGTTGTATCTCAAAATGAGATATGTCAGTATGACAATACGTCACCCTGAACGTTCATAAAACACATATGAAACAACCTTATAACAAAACGAACAACATGAGACAAAACCCGACCTTCCCTAGCTGAACTACCCAAAGTATAAATGCCTGAACAATTAGTTTAGATCCGAGATTCCGCGCTTCCACCACTTAGTATGATTCATATTTTATATAATATATAAGATAAGTAACATTCCGTGAATTAATCTGATAAACTGTTTTGACAACTGGTTACTTCCCTAAGACTGTTTATATTAGGATTGTCAAGACACTCCGGTATTACTCGAGCCCGTAATACAACACCTGGTAGCGTTAAAGGTTACTAATTGTTCAAACGAACCATCGAAAAGCCGAACCTAGCTACACCACACCCCAGTATGAGCTTTATGGATCAAATCCCAGGAGGAGGAAATTATCCAAAACTCCCAGTAGAATGCCTTCCTAACTTCCCGATCCAACCATCTTTGACCTTCAGAGGTAGAAATGACTCGCATAAACTGAAAAACTTTATCTCCGAAATAATGTTAAACATGTCTATGATATCTTGGCCGAATGATGCCAGTCGTATTGTGTACTGCAGAAGACATTTATTAAACCCCGCTGCTCAGTGGGCTAATGACTTTGTACAAGAACAAGGTATACTTGAAATAACATTCGACACATTCATACAAGGATTATATCAGCATTTCTATAAGCCACCAGATATCAATAAAATCTTTAATGCAATCACGCAACTTTCCGAAGCTAAACTTGGTATTGAGCGTCTCAACCAACGATTCAGAAAGATTTGGGACAGAATGCCACCAGACTTCATGACCGAAAAAGCTGCCATAATGACATATACTAGGCTATTGACAAAGGAAACCTATAATATTGTCAGAATGCACAAACCAGAGACATTAAAAGACGCCATGGAAGAGGCTTACCAGACAACTGCACTAACTGAAAGATTCTTCCCAGGATTCGAACTTGATGCTGATGGAGACACTATCATCGGTGCCACAACCCACTTACAAGAAGAATACGACTCTGACTATGATTCAGAAGATAATCTGACCCAGAATGGATACGTCCATACCGTAAGAGCACGTAAGGCGAGTTCTAACCGATCTTGAACTCGAATCAAAAGACCAACAAACTCCTTTTATCAAAACCTTACCAATTGTACACTATATCGCCATCCCCGAGATGGACAATACCGCCGAAAAAACCATAAAAATACAAAACACGAAAGTAAAAACCCTGTTTGACAGTGGATCACCCACGTCATTTATCCGAAGAGATATTGTAGAACTTCTCAAATACGAAATCTACGAGACCCCTCCACTCCGTTTTAGAGGATTCGTAGCCACCAAATCCGCCGTTACATCCGAAGCAGTCACCATTGACCTCAAAATCAATGACCTGCATATAACTTTAGCCGCGTACATACTGGATAACATGGACTACCAATTGTTAATTGGAAATCCAATCTTACGCCGCTACCCGAAAATCCTGCACACAGTACTGAATACCAGAGAGAGCCCCGACTCCTTAAAGCCCAAGACTTATCGCTCCGAAACCGTTAATAACGTTAGAACCTACTCCGCTGGTAATCGTGGTAACCCCAGAAACATAAAACTGTCTTTTGCCCCCACCATTCTCGAAGCAACTGACCCGAAATCCGCTGGTAATCGTGGTGACTCCAGAACCAAAACCCTGTCTCTTGCAACCACTACTCCTGCAGCAATTGACCCGCTTACGACCCTTGATAACCCAGGTAGTACTCAAAGTACATTTGCGCAATTCCCGATACCTGAAGAAGCGAGCATCCTAGAAGAGGATGGAAAATACTCCAACGTTGTCTCAACCATTCAGAGTGTAGAACCTAATGCTACTGATCACAGCAATAAGGACACCTTTTGCACTTTGCCAGTTTGGTTACAACAGAAGTATAGAGAGATCATACGTAATGATCTCCCACCAAGACCTGCCGACATTAATAACATCCCCGTAAAACATGATATTGAAATTAAACCTGGCGCAAGACTACCTCGACTACAGCCATACCATGTTACAGAAAAGAACGAACAAGAAATCAACAAAATAGTTCAAAAACTGCTCGATAACAAGTTCATTGTTCCCTCAAAGTCGCCTTGCAGCTCCCCTGTAGTCCTCGTCCCGAAGAAAGACGGTACCTTCCGACTCTGCGTCGATTACCGCACCCTGAACAAAGCTACCATCTCCGACCCATTCCCATTACCCAGAATCGACAACCTATTGAGCCGTATTGGAAATGCCCAGATATTTACCACGCTAGATTTGCATAGTGGTTACCACCAGATCCCGATGGAACCCAAAGACCGCTACAAAACCGCCTTTGTCACACCATCCGGTAAGTATGAATATACCGTCATGCCATTTGGCTTAGTCAATGCACCTAGTACATTCGCAAGATACATGGCTGATACATTTAGAGACCTGAGATTCGTCAATGTTTACCTTGATGATATATTAATATTCTCCGAATCTCCAGAAGAACATTGGAAACATTTAGACACGGTACTAGAAAGATTAAAGAACGAGAACCTCATTGTTAAGAAGAAAAAATGTAAATTTGCATCTGAAGAAACTGAGTTTTTAGGCTATAGTATTGGAATCCAGAAAATAGCTCCACTACAGCACAAATGTGCAGCAATCCGAGACTTTCCGACGCCTAAAACAGTAAAACAAGCACAGAGATTTTTAGGAATGATTAATTACTACAGACGATTCATTCCAAATTGCTCCAAGATTGCACAGCCAATCCAACTGTTTATTTGTGACAAAAGTCAATGGACAGAAAAACAAGACAAGGCAATTGATAAACTAAAAGACGCCTTGTGTAACTCCCCCGTCCTAGTACCATTCAACAACAAAGCAAACTACCGACTTACAACAGACGCCTCAAAAGACGGCATTGGTGCTGTTCTAGAAGAAGTCGACAACAAGAACAAACTTGTTGGTGTCGTCGGTTACTTCTCTAAATCCTTAGAGAGTGCCCAGAAAAACTATCCTGCTGGCGAATTAGAACTACTTGGAATTATCAAAGCACTCCACCACTTCCGATATATGCTTCACGGAAAGCATTTCACGTTAAGAACAGACCACATTAGTTTGTTATCATTACAAAACAAGAACGAACCCGCACGACGCGTGCAACGCTGGTTAGATGACCTAGCCACATATGACTTCACCTTAGAATACCTAGCTGGACCCAAGAACGTTGTCGCAGATGCCATATCCCGTGCCGTATATACTATAACCCCCGAAACATCCCGACCTATCGACACAGAAAGCTGGAAATCTTACTACAAATCAGACCCATTATGTAGTGCTGTCTTAATTCATATGAAAGAATTGACACAACACAACGTCACACCTGAAGATATGTCAGCCTTCCGTAGTTACCAGAAGAAACTCGAACTATCAGAGACCTTCCGAAAGAATTATTCCCTAGAAGACGAAATGATCTATTACCAAGACCGACTAGTAGTACCAATAAAACAACAGAACGCAGTTATGAGACTATATCATGACCATACCTTATTTGGAGGACATTTTGGTGTAACAGTGACCCTTGCGAAAATCAGCCCAATTTACTATTGGCCAAAATTACAACATTCGATCATACAATACATCAGGACCTGCGTACAATGTCAACTAATAAAATCACACCGACCACGCTTACATGGACTATTACAACCACTCCCTATAGCAGAAGGAAGATGGCTTGATATATCAATGGATTTTGTGACAGGATTACCCCCGACATCAAATAACTTGAATATGATCCTCGTCGTAGTTGATCGTTTTTCGAAACGCGCTCACTTCATAGCTACAAGGAAAACCTTAGACGCAACACAACTAATAGATCTACTCTTTCGATACATTTTTTCATATCATGGTTTTCCCAGGACAATAACCAGTGATAGAGATGTCCGTATGACCGCCGACAAATATCAAGAACTCACGAAAAGACTAGGAATAAAATCGACAATGTCTTCCGCGAACCACCCCCAAACAGATGGACAATCCGAACGAACGATACAGACATTAAACAGGTTACTAAGAGCCTATGCTTCAACCAATATTCAGAATTGGCATGTATATTTACCACAAATCGAATTTGTTTACAATTCTACACCTACTAGAACACTTGGAAAATCACCATTTGAAATTGATTTAGGATATTTACCGAATACCCCTGCTATTAAGTCAGATGACGAAGTCAACGCAAGAAGTTTTACTGCCGTAGAACTTGCCAAACACCTCAAAGCCCTTACCATCCAAACGAAGGAACAGCTAGAACACGCTCAAATCGAAATGGAAACTAATAACAATCAAAGACGTAAACCCTTATTGTTAAACATAGGAGATCACGTATTAGTGCATAGAGATGCATACTTCAAGAAAGGTGCTTATATGAAAGTACAACAAATATACGTCGGACCATTTCGAGTTGTCAAGAAAATAAACGATAACGCCTACGAACTAGATTTAAACTCTCACAAGAAAAAGCACAGAGTTATTAATGTACAATTCCTGAAAAAGTTTGTATACCGTCCAGACGCGTACCCAAAGAATAAACCAATCAGCTCCACTGAAAGAATTAAGAGAGCACACGAAGTTACTGCACTCATAGGAATAGATACTACACACAAAACTTACTTATGTCACATGCAAGATGTAGACCCAACACTTTCAGTAGAATACTCAGAAGCTGAATTTTGCCAAATTCCCGAAAGAACACGAAGATCAATATTAGCCAACTTTAGACAACTCTACGAAACACAAGACAACCCTGAGAGAGAGGAAGATGTTGTATCTCAAAATGAGATATGTCAGTATGACAATACGTCACCCTGAACGTTCATAAAACACATATGAAACAACCTTATAACAAAACGAACAACATGAGACAAAACCCGACCTTCCCTAGCTGAACTACCCAAAGTATAAATGCCTGAACAATTAGTTTAGATCCGAGATTCCGCGCTTCCACCACTTAGTATGATTCATATTTTATATAATATATAAGATAAGTAACATTCCGTGAATTAATCTGATAAACTGTTTTGACAACTGGTTACTTCCCTAAGACTGTTTATATTAGGATTGTCAAGACACTCCGGTATTACTCGAGCCCGTAATACAACA**GCGGCCGCCACCGCGGTGGAGCTCCAATTCGCCCTATAGTGAGTCGTATTACAATTCACTGGCCGTCGTTTTACAACGTCGTGACTGGGAAAACCCTGGCGTTACCCAACTTAATCGCCTTGCAGCACATCCCCCCTTCGCCAGCTGGCGTAATAGCGAAGAGGCCCGCACCGATCGCCCTTCCCAACAGTTGCGCAGCCTGAATGGCGAATGGCGCGACGCGCCCTGTAGCGGCGCATTAAGCGCGGCGGGTGTGGTGGTTACGCGCAGCGTGACCGCTACACTTGCCAGCGCCCTAGCGCCCGCTCCTTTCGCTTTCTTCCCTTCCTTTCTCGCCACGTTCGCCGGCTTTCCCCGTCAAGCTCTAAATCGGGGGCTCCCTTTAGGGTTCCGATTTAGTGCTTTACGGCACCTCGACCCCAAAAAACTTGATTAGGGTGATGGTTCACGTAGTGGGCCATCGCCCTGATAGACGGTTTTTCGCCCTTTGACGTTGGAGTCCACGTTCTTTAATAGTGGACTCTTGTTCCAAACTGGAACAACACTCAACCCTATCTCGGTCTATTCTTTTGATTTATAAGGGATTTTGCCGATTTCGGCCTATTGGTTAAAAAATGAGCTGATTTAACAAAAATTTAACGCGAATTTTAACAAAATATTAACGTTTACAATTTCCTGATGCGGTATTTTCTCCTTACGCATCTGTGCGGTATTTCACACCGCAGGGTAATAACTGATATAATTAAATTGAAGCTCTAATTTGTGAGTTTAGTATACATGCATTTACTTATAATACAGTTTTTTAGTTTTGCTGGCCGCATCTTCTCAAATATGCTTCCCAGCCTGCTTTTCTGTAACGTTCACCCTCTACCTTAGCATCCCTTCCCTTTGCAAATAGTCCTCTTCCAACAATAATAATGTCAGATCCTGTAGAGACCACATCATCCACGGTTCTATACTGTTGACCCAATGCGTCTCCCTTGTCATCTAAACCCACACCGGGTGTCATAATCAACCAATCGTAACCTTCATCTCTTCCACCCATGTCTCTTTGAGCAATAAAGCCGATAACAAAATCTTTGTCGCTCTTCGCAATGTCAACAGTACCCTTAGTATATTCTCCAGTAGATAGGGAGCCCTTGCATGACAATTCTGCTAACATCAAAAGGCCTCTAGGTTCCTTTGTTACTTCTTCTGCCGCCTGCTTCAAACCGCTAACAATACCTGGGCCCACCACACCGTGTGCATTCGTAATGTCTGCCCATTCTGCTATTCTGTATACACCCGCAGAGTACTGCAATTTGACTGTATTACCAATGTCAGCAAATTTTCTGTCTTCGAAGAGTAAAAAATTGTACTTGGCGGATAATGCCTTTAGCGGCTTAACTGTGCCCTCCATGGAAAAATCAGTCAAGATATCCACATGTGTTTTTAGTAAACAAATTTTGGGACCTAATGCTTCAACTAACTCCAGTAATTCCTTGGTGGTACGAACATCCAATGAAGCACACAAGTTTGTTTGCTTTTCGTGCATGATATTAAATAGCTTGGCAGCAACAGGACTAGGATGAGTAGCAGCACGTTCCTTATATGTAGCTTTCGACATGATTTATCTTCGTTTCCTGCAGGTTTTTGTTCTGTGCAGTTGGGTTAAGAATACTGGGCAATTTCATGTTTCTTCAACACTACATATGCGTATATATACCAATCTAAGTCTGTGCTCCTTCCTTCGTTCTTCCTTCTGTTCGGAGATTACCGAATCAAAAAAATTTCAAAGAAACCGAAATCAAAAAAAAGAATAAAAAAAAAATGATGAATTGAATTGAAAAGCGTGGTGCACTCTCAGTACAATCTGCTCTGATGCCGCATAGTTAAGCCAGCCCCGACACCCGCCAACACCCGCTGACGCGCCCTGACGGGCTTGTCTGCTCCCGGCATCCGCTTACAGACAAGCTGTGACCGTCTCCGGGAGCTGCATGTGTCAGAGGTTTTCACCGTCATCACCGAAACGCGCGA

pVB3734

Contents in bold: Ty3-*GAG3*-mCherry (mCherry in red)

Genetic markers: *URA3*, *CEN6/ARS4*, Amp^R^

Construction: The sequence encoding mCherry was fused in frame to the 3’ end of *GAG3*. This region is followed by a partial Ty3 LTR consisting of the U3 region (*POL3* is deleted). The plasmid backbone is pTD3547.

GACGAAAGGGCCTCGTGATACGCCTATTTTTATAGGTTAATGTCATGATAATAATGGTTTCTTAGGACGGATCGCTTGCCTGTAACTTACACGCGCCTCGTATCTTTTAATGATGGAATAATTTGGGAATTTACTCTGTGTTTATTTATTTTTATGTTTTGTATTTGGATTTTAGAAAGTAAATAAAGAAGGTAGAAGAGTTACGGAATGAAGAAAAAAAAATAAACAAAGGTTTAAAAAATTTCAACAAAAAGCGTACTTTACATATATATTTATTAGACAAGAAAAGCAGATTAAATAGATATACATTCGATTAACGATAAGTAAAATGTAAAATCACAGGATTTTCGTGTGTGGTCTTCTACACAGACAAGATGAAACAATTCGGCATTAATACCTGAGAGCAGGAAGAGCAAGATAAAAGGTAGTATTTGTTGGCGATCCCCCTAGAGTCTTTTACATCTTCGGAAAACAAAAACTATTTTTTCTTTAATTTCTTTTTTTACTTTCTATTTTTAATTTATATATTTATATTAAAAAATTTAAATTATAATTATTTTTATAGCACGTGATGAAAAGGACCCAGGTGGCACTTTTCGGGGAAATGTGCGCGGAACCCCTATTTGTTTATTTTTCTAAATACATTCAAATATGTATCCGCTCATGAGACAATAACCCTGATAAATGCTTCAATAATATTGAAAAAGGAAGAGTATGAGTATTCAACATTTCCGTGTCGCCCTTATTCCCTTTTTTGCGGCATTTTGCCTTCCTGTTTTTGCTCACCCAGAAACGCTGGTGAAAGTAAAAGATGCTGAAGATCAGTTGGGTGCACGAGTGGGTTACATCGAACTGGATCTCAACAGCGGTAAGATCCTTGAGAGTTTTCGCCCCGAAGAACGTTTTCCAATGATGAGCACTTTTAAAGTTCTGCTATGTGGCGCGGTATTATCCCGTATTGACGCCGGGCAAGAGCAACTCGGTCGCCGCATACACTATTCTCAGAATGACTTGGTTGAGTACTCACCAGTCACAGAAAAGCATCTTACGGATGGCATGACAGTAAGAGAATTATGCAGTGCTGCCATAACCATGAGTGATAACACTGCGGCCAACTTACTTCTGACAACGATCGGAGGACCGAAGGAGCTAACCGCTTTTTTTCACAACATGGGGGATCATGTAACTCGCCTTGATCGTTGGGAACCGGAGCTGAATGAAGCCATACCAAACGACGAGCGTGACACCACGATGCCTGTAGCAATGGCAACAACGTTGCGCAAACTATTAACTGGCGAACTACTTACTCTAGCTTCCCGGCAACAATTAATAGACTGGATGGAGGCGGATAAAGTTGCAGGACCACTTCTGCGCTCGGCCCTTCCGGCTGGCTGGTTTATTGCTGATAAATCTGGAGCCGGTGAGCGTGGGTCTCGCGGTATCATTGCAGCACTGGGGCCAGATGGTAAGCCCTCCCGTATCGTAGTTATCTACACGACGGGCAGTCAGGCAACTATGGATGAACGAAATAGACAGATCGCTGAGATAGGTGCCTCACTGATTAAGCATTGGTAACTGTCAGACCAAGTTTACTCATATATACTTTAGATTGATTTAAAACTTCATTTTTAATTTAAAAGGATCTAGGTGAAGATCCTTTTTGATAATCTCATGACCAAAATCCCTTAACGTGAGTTTTCGTTCCACTGAGCGTCAGACCCCGTAGAAAAGATCAAAGGATCTTCTTGAGATCCTTTTTTTCTGCGCGTAATCTGCTGCTTGCAAACAAAAAAACCACCGCTACCAGCGGTGGTTTGTTTGCCGGATCAAGAGCTACCAACTCTTTTTCCGAAGGTAACTGGCTTCAGCAGAGCGCAGATACCAAATACTGTCCTTCTAGTGTAGCCGTAGTTAGGCCACCACTTCAAGAACTCTGTAGCACCGCCTACATACCTCGCTCTGCTAATCCTGTTACCAGTGGCTGCTGCCAGTGGCGATAAGTCGTGTCTTACCGGGTTGGACTCAAGACGATAGTTACCGGATAAGGCGCAGCGGTCGGGCTGAACGGGGGGTTCGTGCACACAGCCCAGCTTGGAGCGAACGACCTACACCGAACTGAGATACCTACAGCGTGAGCATTGAGAAAGCGCCACGCTTCCCGAAGGGAGAAAGGCGGACAGGTATCCGGTAAGCGGCAGGGTCGGAACAGGAGAGCGCACGAGGGAGCTTCCAGGGGGGAACGCCTGGTATCTTTATAGTCCTGTCGGGTTTCGCCACCTCTGACTTGAGCGTCGATTTTTGTGATGCTCGTCAGGGGGGCCGAGCCTATGGAAAAACGCCAGCAACGCGGCCTTTTTACGGTTCCTGGCCTTTTGCTGGCCTTTTGCTCACATGTTCTTTCCTGCGTTATCCCCTGATTCTGTGGATAACCGTATTACCGCCTTTGAGTGAGCTGATACCGCTCGCCGCAGCCGAACGACCGAGCGCAGCGAGTCAGTGAGCGAGGAAGCGGAAGAGCGCCCAATACGCAAACCGCCTCTCCCCGCGCGTTGGCCGATTCATTAATGCAGCTGGCACGACAGGTTTCCCGACTGGAAAGCGGGCAGTGAGCGCAACGCAATTAATGTGAGTTAGCTCACTCATTAGGCACCCCAGGCTTTACACTTTATGCTTCCGGCTCGTATGTTGTGTGGAATTGTGAGCGGATAACAATTTCACACAGGAAACAGCTATGACCATGATTACGCCAAGCTCGAAATTAACCCTCACTAAAGGGAACAAAAGCTGTCGAGACGGTATCGATAAGCTT**TGTTGTATCTCAAAATGAGATATGTCAGTATGACAATACGTCACCCTGAACGTTCATAAAACACATATGAAACAACCTTATAACAAAACGAACAACATGAGACAAAACCCGACCTTCCCTAGCTGAACTACCCAAAGTATAAATGCCTGAACAATTAGTTTAGATCCGAGATTCCGCGCTTCCACCACTTAGTATGATTCATATTTTATATAATATATAAGATAAGTAACATTCCGTGAATTAATCTGATAAACTGTTTTGACAACTGGTTACTTCCCTAAGACTGTTTATATTAGGATTGTCAAGACACTCCGGTATTCCTGGTAGCGTTAAAGGTTACTAATTGTTCAAACGAACCATCGAAAAGCCGAACCTAGCTACACCACACCCCAGTATGAGCTTTATGGATCAAATCCCAGGAGGAGGAAATTATCCAAAACTCCCAGTAGAATGCCTTCCTAACTTCCCGATCCAACCATCTTTGACCTTCAGAGGTAGAAATGACTCGCATAAACTGAAAAACTTTATCTCCGAAATAATGTTAAACATGTCTATGATATCTTGGCCGAATGATGCCAGTCGTATTGTGTACTGCAGAAGACATTTATTAAACCCCGCTGCTCAGTGGGCTAATGACTTTGTACAAGAACAAGGTATACTTGAAATAACATTCGACACATTCATACAAGGATTATATCAGCATTTCTATAAGCCACCAGATATCAATAAAATCTTTAATGCAATCACGCAACTTTCCGAAGCTAAACTTGGTATTGAGCGTCTCAACCAACGATTCAGAAAGATTTGGGACAGAATGCCACCAGACTTCATGACCGAAAAAGCTGCCATAATGACATATACTAGGCTATTGACAAAGGAAACCTATAATATTGTCAGAATGCACAAACCAGAGACATTAAAAGACGCCATGGAAGAGGCTTACCAGACAACTGCACTAACTGAAAGATTCTTCCCAGGATTCGAACTTGATGCTGATGGAGACACTATCATCGGTGCCACAACCCACTTACAAGAAGAATACGACTCTGACTATGATTCAGAAGATAATCTGACCCAGAATGGATACGTCCATACCGTAAGGACAAGAAGATCTTACAATAAACCAATGTCAAATCATCGAAACAGGAGAAATAACAACCCATCTAGAGAAGAATGTATAAAAAATCGGCTATGCTTCTATTGTAAGAAAGAGGGACATCGCCTGAACGAATGTAGAGCACGTAAGGCGAGTTCTAACCGATCTCCCGGGATGGTGAGCAAGGGCGAGGAGGATAACATGGCCATCATCAAGGAGTTCATGCGCTTCAAGGTGCACATGGAGGGCTCCGTGAACGGCCACGAGTTCGAGATCGAGGGCGAGGGCGAGGGCCGCCCCTACGAGGGCACCCAGACCGCCAAGCTGAAGGTGACCAAGGGTGGCCCCCTGCCCTTCGCCTGGGACATCCTGTCCCCTCAGTTCATGTACGGCTCCAAGGCCTACGTGAAGCACCCCGCCGACATCCCCGACTACTTGAAGCTGTCCTTCCCCGAGGGCTTCAAGTGGGAGCGCGTGATGAACTTCGAGGACGGCGGCGTGGTGACCGTGACCCAGGACTCCTCCCTGCAGGACGGCGAGTTCATCTACAAGGTGAAGCTGCGCGGCACCAACTTCCCCTCCGACGGCCCCGTAATGCAGAAGAAGACCATGGGCTGGGAGGCCTCCTCCGAGCGGATGTACCCCGAGGACGGCGCCCTGAAGGGCGAGATCAAGCAGAGGCTGAAGCTGAAGGACGGCGGCCACTACGACGCTGAGGTCAAGACCACCTACAAGGCCAAGAAGCCCGTGCAGCTGCCCGGCGCCTACAACGTCAACATCAAGTTGGACATCACCTCCCACAACGAGGACTACACCATCGTGGAACAGTACGAACGCGCCGAGGGCCGCCACTCCACCGGCGGCATGGACGAGCTGTACAAGTAGCCCGGGACGTTCATAAAACACATATGAAACAACCTTATAACAAAACGAACAACATGAGACAAAACCCGACCTTCCCTAGCTGAACTACCCAAAGTATAAATGCCTGAACAATTAGTTTAGATCCGAGATTCCGCGCTTCCACCACTTAGTATGATTCATATTTTATATAATATATAAGATAAGTAACATTCCGTGAATTAATCTGATAAACTGTTTTGACAACTGGTTACTTCCCTAAGACTGTTTATATTAGGATTGTCAAGACACTCCGGTATTACTCGAGCCCGTA**ATACAACAGCGGCCGCCACCGCGGTGGAGCTCCAATTCGCCCTATAGTGAGTCGTATTACAATTCACTGGCCGTCGTTTTACAACGTCGTGACTGGGAAAACCCTGGCGTTACCCAACTTAATCGCCTTGCAGCACATCCCCCCTTCGCCAGCTGGCGTAATAGCGAAGAGGCCCGCACCGATCGCCCTTCCCAACAGTTGCGCAGCCTGAATGGCGAATGGCGCGACGCGCCCTGTAGCGGCGCATTAAGCGCGGCGGGTGTGGTGGTTACGCGCAGCGTGACCGCTACACTTGCCAGCGCCCTAGCGCCCGCTCCTTTCGCTTTCTTCCCTTCCTTTCTCGCCACGTTCGCCGGCTTTCCCCGTCAAGCTCTAAATCGGGGGCTCCCTTTAGGGTTCCGATTTAGTGCTTTACGGCACCTCGACCCCAAAAAACTTGATTAGGGTGATGGTTCACGTAGTGGGCCATCGCCCTGATAGACGGTTTTTCGCCCTTTGACGTTGGAGTCCACGTTCTTTAATAGTGGACTCTTGTTCCAAACTGGAACAACACTCAACCCTATCTCGGTCTATTCTTTTGATTTATAAGGGATTTTGCCGATTTCGGCCTATTGGTTAAAAAATGAGCTGATTTAACAAAAATTTAACGCGAATTTTAACAAAATATTAACGTTTACAATTTCCTGATGCGGTATTTTCTCCTTACGCATCTGTGCGGTATTTCACACCGCAGGGTAATAACTGATATAATTAAATTGAAGCTCTAATTTGTGAGTTTAGTATACATGCATTTACTTATAATACAGTTTTTTAGTTTTGCTGGCCGCATCTTCTCAAATATGCTTCCCAGCCTGCTTTTCTGTAACGTTCACCCTCTACCTTAGCATCCCTTCCCTTTGCAAATAGTCCTCTTCCAACAATAATAATGTCAGATCCTGTAGAGACCACATCATCCACGGTTCTATACTGTTGACCCAATGCGTCTCCCTTGTCATCTAAACCCACACCGGGTGTCATAATCAACCAATCGTAACCTTCATCTCTTCCACCCATGTCTCTTTGAGCAATAAAGCCGATAACAAAATCTTTGTCGCTCTTCGCAATGTCAACAGTACCCTTAGTATATTCTCCAGTAGATAGGGAGCCCTTGCATGACAATTCTGCTAACATCAAAAGGCCTCTAGGTTCCTTTGTTACTTCTTCTGCCGCCTGCTTCAAACCGCTAACAATACCTGGGCCCACCACACCGTGTGCATTCGTAATGTCTGCCCATTCTGCTATTCTGTATACACCCGCAGAGTACTGCAATTTGACTGTATTACCAATGTCAGCAAATTTTCTGTCTTCGAAGAGTAAAAAATTGTACTTGGCGGATAATGCCTTTAGCGGCTTAACTGTGCCCTCCATGGAAAAATCAGTCAAGATATCCACATGTGTTTTTAGTAAACAAATTTTGGGACCTAATGCTTCAACTAACTCCAGTAATTCCTTGGTGGTACGAACATCCAATGAAGCACACAAGTTTGTTTGCTTTTCGTGCATGATATTAAATAGCTTGGCAGCAACAGGACTAGGATGAGTAGCAGCACGTTCCTTATATGTAGCTTTCGACATGATTTATCTTCGTTTCCTGCAGGTTTTTGTTCTGTGCAGTTGGGTTAAGAATACTGGGCAATTTCATGTTTCTTCAACACTACATATGCGTATATATACCAATCTAAGTCTGTGCTCCTTCCTTCGTTCTTCCTTCTGTTCGGAGATTACCGAATCAAAAAAATTTCAAAGAAACCGAAATCAAAAAAAAGAATAAAAAAAAAATGATGAATTGAATTGAAAAGCGTGGTGCACTCTCAGTACAATCTGCTCTGATGCCGCATAGTTAAGCCAGCCCCGACACCCGCCAACACCCGCTGACGCGCCCTGACGGGCTTGTCTGCTCCCGGCATCCGCTTACAGACAAGCTGTGACCGTCTCCGGGAGCTGCATGTGTCAGAGGTTTTCACCGTCATCACCGAAACGCGCGA
